# Diagnosis of pediatric central nervous system tumors using methylation profiling of cfDNA from cerebrospinal fluid

**DOI:** 10.1186/s13148-024-01696-w

**Published:** 2024-07-05

**Authors:** Lotte Cornelli, Ruben Van Paemel, Maísa R. Ferro dos Santos, Sofie Roelandt, Leen Willems, Jelle Vandersteene, Edward Baert, Liselot M. Mus, Nadine Van Roy, Bram De Wilde, Katleen De Preter

**Affiliations:** 1 Department of Biomolecular Medicine, Ghent University, Ghent, Belgium; 2 Center for Medical Biotechnology, VIB-UGent, Ghent, Belgium; 3 Cancer Research Institute Ghent, Ghent, Belgium; 4 Department of Pediatric Hematology, Oncology and Stem Cell Transplantation, Ghent University Hospital, Ghent, Belgium; 5 Department of Internal Medicine and Pediatrics, Ghent University, Ghent, Belgium; 6 Department of Neurosurgery, Ghent University Hospital, Ghent, Belgium

**Keywords:** Pediatric oncology, DNA methylation, Liquid biopsy, Central nervous system tumor, Precision medicine, Cerebrospinal fluid

## Abstract

Pediatric central nervous system tumors remain challenging to diagnose. Imaging approaches do not provide sufficient detail to discriminate between different tumor types, while the histopathological examination of tumor tissue shows high inter-observer variability. Recent studies have demonstrated the accurate classification of central nervous system tumors based on the DNA methylation profile of a tumor biopsy. However, a brain biopsy holds significant risk of bleeding and damaging the surrounding tissues. Liquid biopsy approaches analyzing circulating tumor DNA show high potential as an alternative and less invasive tool to study the DNA methylation pattern of tumors. Here, we explore the potential of classifying pediatric brain tumors based on methylation profiling of the circulating cell-free DNA (cfDNA) in cerebrospinal fluid (CSF). For this proof-of-concept study, we collected cerebrospinal fluid samples from 19 pediatric brain cancer patients via a ventricular drain placed for reasons of increased intracranial pressure. Analyses on the cfDNA showed high variability of cfDNA quantities across patients ranging from levels below the limit of quantification to 40 ng cfDNA per milliliter of CSF. Classification based on methylation profiling of cfDNA from CSF was correct for 7 out of 20 samples in our cohort. Accurate results were mostly observed in samples of high quality, more specifically those with limited high molecular weight DNA contamination. Interestingly, we show that centrifugation of the CSF prior to processing increases the fraction of fragmented cfDNA to high molecular weight DNA. In addition, classification was mostly correct for samples with high tumoral cfDNA fraction as estimated by computational deconvolution (> 40%). In summary, analysis of cfDNA in the CSF shows potential as a tool for diagnosing pediatric nervous system tumors especially in patients with high levels of tumoral cfDNA in the CSF. Further optimization of the collection procedure, experimental workflow and bioinformatic approach is required to also allow classification for patients with low tumoral fractions in the CSF.

**Supplementary Information:**

The online version contains supplementary material available at 10.1186/s13148-024-01696-w.

## Introduction

Intracranial central nervous system (CNS) tumors are one of the leading causes of cancer-related death in children after leukemia [1, 2]. Diagnosis of these brain tumors is complex as they consist of a heterogeneous group of tumors, from slow-growing, low-grade lesions to high-grade cancers [3]. An accurate and detailed diagnosis is vital for treatment decisions and determines patient outcome [4, 5]. The current diagnostic procedure of (pediatric) brain tumors requires a tumor tissue biopsy for histopathological investigation [6–8]. In this evaluation, inter-observatory variability occasionally results in misdiagnosis [6–8]. Diagnostic procedures are evolving from purely histology-based methods [9], to a combined approach where assays that interrogate molecular markers are becoming increasingly important [4]. Indeed, the latest 2021 World Health Organization (WHO) classification entails tumor types and subtypes that can only be distinguished by combinations of new molecular profiling methods [5, 10]. Genomic and epigenomic analyses have improved the diagnostic process for many cancer entities. More specifically, the tumor DNA methylation profile is shown to be tissue-specific and therefore is a powerful tool for tumor classification [11–14], as convincingly shown for brain tumor (sub-)classification [11].

For brain cancer patients with delicate tumor location, performing a biopsy or resection can hold disproportional risks [15–18]. In the last decade, the potential of the use of liquid biopsies has become evident, emerging as a novel and valuable approach to molecularly investigate the tumor in a minimally invasive manner [19, 20]. Tumoral biomolecules including circulating cell-free DNA (cfDNA), RNA and proteins are released from different locations within the tumor and therefore contain molecular information while avoiding sampling bias often seen in tissue biopsies [15, 18, 20]. These molecules are found in biofluids surrounding the tumor, including blood, urine, cerebrospinal fluid and others [20]. Several studies have shown that the amount of circulating tumor DNA (ctDNA) in the blood is limited in patients with intracranial tumors due to retention by the blood–brain barrier [18, 21–23]. In these studies, cerebrospinal fluid is suggested as a superior source for ctDNA. The extraction of CSF is often an uncomfortable and invasive procedure, requiring a painful lumbar puncture to acquire fluid from the space surrounding the spinal cord. However, pediatric patients often present with symptoms of increased intracranial pressure due to CSF flow obstruction, requiring emergency placement of an external ventricular drain to remove the excess fluids [24–26]. In some cases, an additional lumbar puncture is performed after removal of the tumor, to examine the CSF for the presence of circulating tumor cells in cases where there is risk for cerebrospinal fluid metastasis [24, 27–29]. For several diagnoses, CSF is collected during standard diagnostic procedures and collection for cfDNA would not require additional procedures.

Published cfDNA studies on CSF have mostly focused on tumor follow-up rather than classification. For example, cfDNA is used for mutation detection where tumors are detected based on the previously defined patient-/tumor-specific mutations [30–35]. Since mutations frequently fail to differentiate between tumor subtypes, Li and colleagues instead used whole-genome bisulfite sequencing (WGBS)-based DNA methylation profiling and hydroxymethylation profiling through anti-CMS immunoprecipitation sequencing to subtype and monitor medulloblastoma tumors [36]. A recent study utilized nanopore technology to investigate tumor classification [37]. Here, Afflerbach et al. conducted nanopore sequencing on cerebrospinal fluid cfDNA (CSF-cfDNA) to classify tumors based on copy number variation (CNV) profiles and methylation patterns. Classification based on CNV profiling led to an accurate diagnosis for 44 out of 129 sequenced samples. Although these CNV data give valuable information for diagnoses of CNS tumors, DNA methylation profiling can identify tumor types and subtypes with more nuanced differences [11]. DNA methylation profiling using nanopore sequencing, was successful in detecting tumoral DNA based on methylation profiling in 24 out of 129 sequenced samples. Out of these 24 samples, accurate classification was achieved for 22 of them [37]. However, the proposed workflow requires a minimal input 1 ml CSF and 5 ng cfDNA, which can be challenging in CSF collections. Additionally, samples in this study were lost after failing the technical pass of 100 000 reads, or were unsuited for methylation profiling when they did not cover a minimal of 1000 CpGs [37].

Considering the significant expense associated with the use of WGBS and the fact that nanopore sequencing of cfDNA has yielded successful results only in a subset of samples, we have generated a proof-of-concept study for the use of an alternative technology that allows accurate and minimally invasive classification of pediatric brain tumors. More specifically, we use cell-free reduced representation bisulfite sequencing (cfRRBS) that allows methylation profiling of low amounts of fragmented DNA, such as cfDNA [38]. The method involves a step to enrich the more relevant methylated regions, i.e., the CpG-rich regions, resulting in a reduction of sequencing cost per sample compared to WGBS. Before, we (Van Paemel et al.) have demonstrated the potential of cfRRBS for accurate cfDNA methylation-based diagnosis of pediatric solid tumors [39], by achieving a correct classification rate of 94% in high-quality samples, mostly cfDNA from blood plasma. In this pilot study, we explore the diagnostic potential of cfRRBS followed by DNA methylation signal deconvolution for tumor fraction estimation on cfDNA isolated from cerebrospinal fluids in pediatric brain tumor patients.

## Material and methods

### Patients and samples

This study was approved by the ethical committee and informed consent was obtained from all patients and/or their representatives. Pediatric patients presenting with a central nervous system tumor at Ghent University Hospital from February 2020 to July 2023 (*n* = 19; Age range 3 months to 16 years old) that required ventricular drainage were included. Samples were collected from patients that were pathologically diagnosed with medulloblastoma (*n* = 6), pilocytic astrocytoma (*n* = 6), ependymoma (*n* = 3; two samples were collected from the same patient), choroid plexus papilloma (*n* = 2), diffuse midline glioma (*n* = 1), adamantinomatous craniopharyngioma (*n* = 1) and atypical teratoid/rhabdoid tumor (*n* = 1). Complete patient and sample data are available in the supplemental tables 1 and 2.

### CSF collection

Determined by the amount that could safely be sampled, 1.5 to 20 ml of cerebrospinal fluid was collected. For the first 12 patients, samples were separated into two aliquots. One aliquot was centrifuged for 10 min at 1900 g and the supernatant transferred to a clean 15 ml falcon tube. The other aliquot was processed without additional interventions. For the following patients, the complete CSF sample was centrifuged. Both the unprocessed CSF and the supernatant after centrifugation were stored at − 80 °C CSF. For 13 patients centrifugation was performed immediately and samples were stored within four hours after collection. For the remaining patients (*n* = 7/20), samples were temporarily frozen at the operation room at − 20 °C before any processing. These samples were subsequently stored at − 80 °C and centrifuged on the day of further processing.

### Circulating cell-free DNA extraction and quality control

CSF samples were thawed to room temperature and cfDNA was extracted using a Maxwell RSC LV ccfDNA kit (Promega). Depending on sample availability, between 1 and 7.5 ml of CSF was used as input for cfDNA extraction. In order to reach the minimal input for this protocol, one sample with a volume below 2 ml was supplemented with PBS (1X, Gibco) to a total volume of 2 ml, prior to addition of binding buffer. The extraction was performed according to the manufacturer’s guidelines and the resulting cfDNA was eluted in 75 µl of elution buffer supplied in the kit (Promega).

The cfDNA concentration was measured using Fluoroskan™ Microplate Fluorometer (Thermo Fisher Scientific) according to the manufacturer’s instructions. Size distribution profiles were obtained using Agilent Tapestation with the Cell-Free DNA ScreenTape kit. CfDNA was defined as DNA fragments with lengths ranging between 70 and 700 bp and high molecular weight DNA (HMW-DNA) as DNA fragments with lengths exceeding 700 bp. Isolated DNA was stored at − 20 °C until further processing.

### DNA isolation from surgical tumor biopsy samples

An aliquot of isolated genomic DNA from a tumor tissue biopsy was obtained for 11 of the 19 patients. DNA was extracted starting from 3 to 15 formalin-fixed paraffin-embedded (FFPE) tissue slides according to the manufacturer’s instructions using the Qiamp DNA FFPE Tissue kit (QIAGEN). DNA was stored at 4 °C until further processing.

### cfRRBS library preparation

Isolated DNA was processed using cell-free reduced representation bisulfite sequencing as previously described [40]. For the initial 12 patients, cfDNA from whole CSF as well as the centrifuged aliquot was used as input for cfRRBS. When available (*n* = 27/32), 10 ng of DNA was used as input as described in the protocol. For samples with a yield lower than 10 ng, we used inputs ranging from 0.5 to 7.2 ng depending on the availability of material. Samples with a DNA concentration below 0.2 ng/µL were concentrated via vacuum centrifugation (SpeedVac, Thermo Fischer Scientific) at 45 °C. According to the protocol, 0.01 ng lambda spike-in was added to the samples. After library amplification, DNA was purified using SPRI bead size selection (AMPure XT beads—NEB), with 2.5 × proportion of bead to sample volume. The libraries were quantified and checked for the presence of adapter dimers via the Kapa library quantification kit for Illumina platforms (Kapa Biosystems). The length profile was visualized via Fragment Analyzer (Advanced Analytical Technologies). Samples were pooled equimolarly to a total concentration of 4 nM. Final concentration of the pooled samples was verified using the Kapa library quantification kit for Illumina platforms (Kapa Biosystems).

### Sequencing quality control and mapping

Samples were sequenced on a NovaSeq 6000 instrument using a NovaSeq SP or S1 kit (paired-end, 2 × 50 cycles), supplemented with 3% phiX and a loading concentration between 750 and 800 pM. Samples from different donors were mixed to avoid sequencing batch effects. BCL files were demultiplexed and quality checked as previously described by Van Paemel et al. [39]. Updated modules were used for demultiplexing (bcl2fastq v2.20), adapter removal (Trim Galore v0.6.6 and CutAdapt), mapping (SAMtools v.1.14 and Bismark v.0.23.1), read counting (Picard tools v.2.21.6). Visualization of the results was done with R version 4.3.2 and ggplot2 v 3.4.4. We obtained on average 21.7 M reads and a minimum of 7 M reads per sample. Mapping efficiency was on average 52%, as to be expected for cfRRBS data; bisulfite conversion was at least 95.9% for all samples and exceeded 98% for 25/32 samples. Full QC report of the samples is shared in supplementary Table 3.

### Development of a reference set for computational deconvolution

The methylation profiles of 2801 brain tumors generated on Illumina array 450 K platform, encompassing 81 different brain tumor entities, was obtained from Capper et al. [11]. The reference set was adjusted to allow deconvolution of cfRRBS data by only considering the CpGs in regions that overlap in the cfRRBS and array data as described by Van Paemel et al. [39]. In-house data of healthy plasma cfDNA were also included in the reference dataset, as well as published methylation data of prepuberal white blood cells (WBC, *n* = 52) [41] as some samples with red discoloration are assumed to contain low volumes of contaminating blood due to placement of the ventricular drain.

### Computational deconvolution of cellular fractions

Reference and test samples were grouped in 14.103 clusters, defined by the overlapping regions analyzed by cfRRBS (CSF samples) and 450 K arrays (reference data). A beta value was calculated for each cluster, defined as a median value for the methylation status. These beta values of all clusters were used for deconvolution. Tumoral fractions were estimated using Methatlas [42], a nonlinear least square-based deconvolution method. (https://github.com/rmvpaeme/cfRRBS_classifier) Annotation of the samples was done based on the tumor entity with the highest estimated fraction (excluding non-tumoral signals). Full deconvolution results from both CSF-cfDNA and FFPE samples can be found in supplementary Table 4.

### Copy number profiles

Copy number profiles were inferred from the cfRRBS data of both CSF liquids and tissue biopsies. We used WisecondorX (https://github.com/CenterForMedicalGeneticsGhent/WisecondorX) to detect CNVs after mapping to the bisulfite converted genome. The binsize was set at 400 kb [43]. Samples were normalized with an in-house dataset of cfRRBS data from healthy volunteers.

## Results

### Reference dataset for computational deconvolution of pediatric brain tumor fractions

**Fig. 1:**
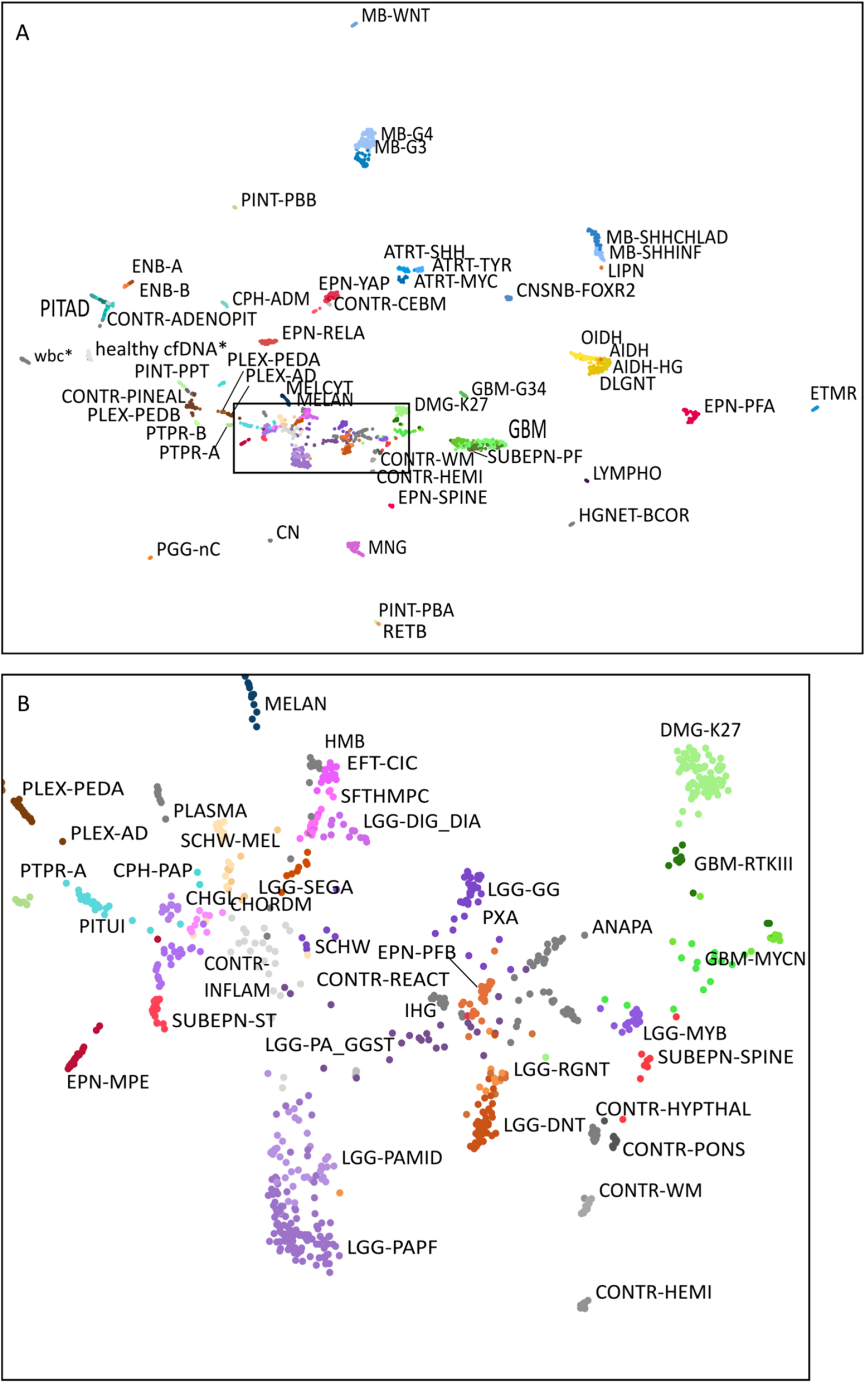
**A** UMAP visualization of the DNA methylation reference dataset that is built for computational deconvolution of pediatric brain tumor classification. Only data from regions covered by both cfRRBS and Illumina 450 k arrays are included. The two sample groups supplemented to the published dataset of Capper et al. are indicated with an *. **B** Zoom-in on the low-grade glioma (LGG) clusters that overlap with other tumor types

For our reference dataset, we modified a published Illumina array dataset to align with the genomic regions covered in cfRRBS data (details in M&M). In the reference set, we also included cfRRBS data of blood plasma cfDNA from non-cancerous volunteers, as well as array data from prepubescent white blood cells [41] because some CSF samples present with contaminating blood cells. We performed UMAP dimensionality reduction on the cfRRBS reference dataset to visualize the grouping/clustering of the tumor entities based on their methylation profile (Fig. 1A). While most tumor entities can be clearly distinguished in this plot, similar as the published visualization by Capper et al. [11] we observed that the low-grade glioma clusters overlap with other tumor entities (Fig. 1B). This optimized dataset is used as reference for computational deconvolution of pediatric brain tumor fractions in the next paragraphs.

### Correct tumor classification using cfRRBS on pediatric brain tumor tissue DNA

**Fig. 2:**
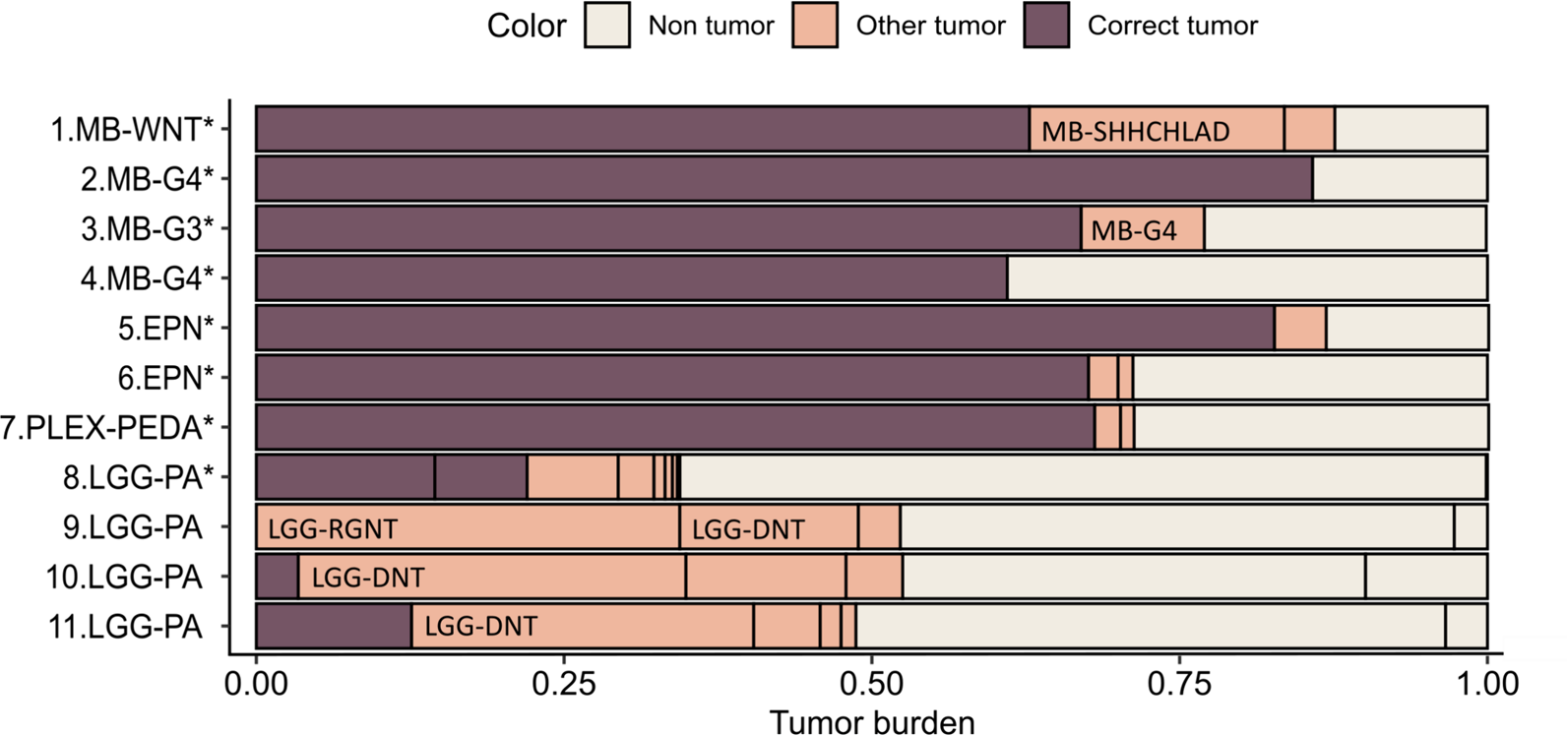
Visualization of the estimated tumor fraction according to computational deconvolution based on DNA methylation profiles of the tumor tissue samples. The fraction of the histopathological diagnosis is indicated in brown; the other estimated tumor fractions that do not correspondent with the histopathological diagnosis are indicated in orange and the non-tumoral fraction in beige. Samples are classified correctly when the highest estimated tumoral fraction corresponds to the diagnosis, indicated with an asterisk. Table with full classification results for each sample is available in supplemental Table 4

To validate our deconvolution-based classification pipeline, we first applied it on cfRRBS profiles generated on genomic DNA isolated from pediatric brain tumor tissue of 11 patients. For 8 out of 11 tumors, the highest estimated tumor fraction corresponds to the histopathological diagnosis. The highest tumor fraction estimated using deconvolution for all samples diagnosed with medulloblastoma tumor (MB; *n* = 4), ependymoma tumor (EPN; *n* = 2) and choroid plexus papilloma tumor (PLEX; *n* = 1) corresponds to the histopathological diagnosis and assigned subclass (Fig. 2). However, for pilocytic astrocytoma (LGG-PA; *n* = 4) only one out of 4 tumors is classified correctly. Given the inter-observer variability that is reported for histopathological diagnoses, a pathologist re-examined these 3 cases but excluded any misdiagnosis. The incorrect classification can be explained by the fact that the DNA methylation profile of LGG-PA cases is not distinct enough as observed in Fig. 1B.

### cfDNA in cerebrospinal fluid

We collected CSF samples of 19 pediatric patients presenting with CNS tumors. For one patient, two CSF samples were collected at the moment of two consecutive relapses. In this cohort, we considered these two samples as independent due to our primary emphasis on the sample quality features. All samples were collected in plastic containers without preservatives. When possible, collections were processed immediately (*n* = 13), if not they were frozen at − 20 °C in the operation room (*n* = 7). Previous studies have shown that high molecular weight DNA (HMW-DNA) originating from white blood cells can interfere with cfRRBS and deconvolution by diluting the tumoral signal [39]. For that reason, CSF was centrifuged with the aim of removing cell debris that could contribute to HMW-DNA contamination. For 12 patients, we also stored an aliquot of uncentrifuged material to evaluate the effect of the centrifugation.

**Fig. 3:**
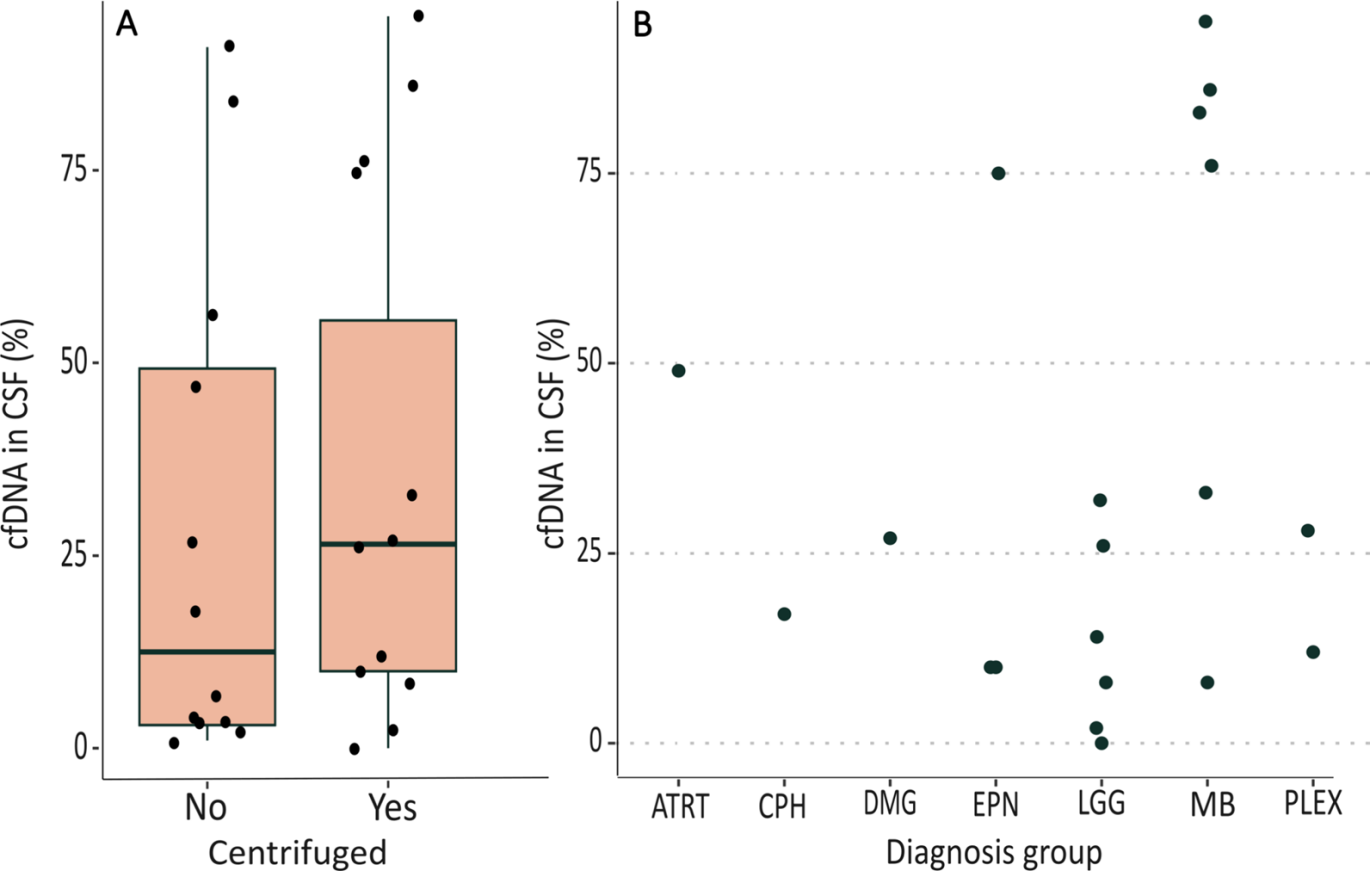
Percentage of cfDNA (70–700 bp) over total DNA isolated from CSF. **A** cfDNA fraction over total DNA in whole CSF (*n* = 12) versus the matched centrifuged CSF samples (*n* = 12). **B** cfDNA fraction over total DNA per tumor type for centrifuged samples. Included tumor types are atypical teratoid rhabdoid tumor (ATRT), adamantinomatous craniopharyngioma (CPH), diffuse midline glioma (DMG), ependymoma (EPN), low-grade glioma tumors (LGG), medulloblastoma (MB), choroid plexus papilloma (PLEX)

Next, cfDNA was isolated, concentration quantified and fragment length analyzed. We observed a high degree of variability in the total amount of cfDNA and fragment profile between patients. Samples from three patients showed a cfDNA (70–700 bp length) concentration that was below the limit of detection by Tapestation visualization. Compared to centrifuged CSF, whole CSF samples showed a significantly lower cfDNA concentration on total DNA concentration (Fig. 3, *p* value 0.04814) pointing at more HMW-DNA contamination. The total yield of cfDNA is not significantly different between whole and centrifuged CSF (*p* value = 0.4962; figure in supplemental information), indicating that centrifugation primarily decreases the presence of HMW-DNA by removing cells and thus preventing cell lysis in the sample, and has minimal effect on the fragmented cfDNA. Based on these results, we decided to centrifuge the CSF before processing for the other 8 samples (sample 13 to sample 20). Figure 3 shows the percentage of cfDNA over total DNA that was isolated from CSF after centrifugation of each patient.

### Deconvolution of tumor fractions in cfDNA from CSF

Following quality control, all samples were processed with cfRRBS library preparation, sequencing and deconvolution to estimate the tumoral and healthy cfDNA fractions. The estimated tumor type (i.e., entity with the highest predicted tumor fraction after using deconvolution) corresponded with the histopathological diagnosis for 7 out of 20 cases: medulloblastoma (4/5), ependymoma (1/3), choroid plexus papilloma (1/2), atypical teratoid rhabdoid tumor (1/1).

**Fig. 4:**
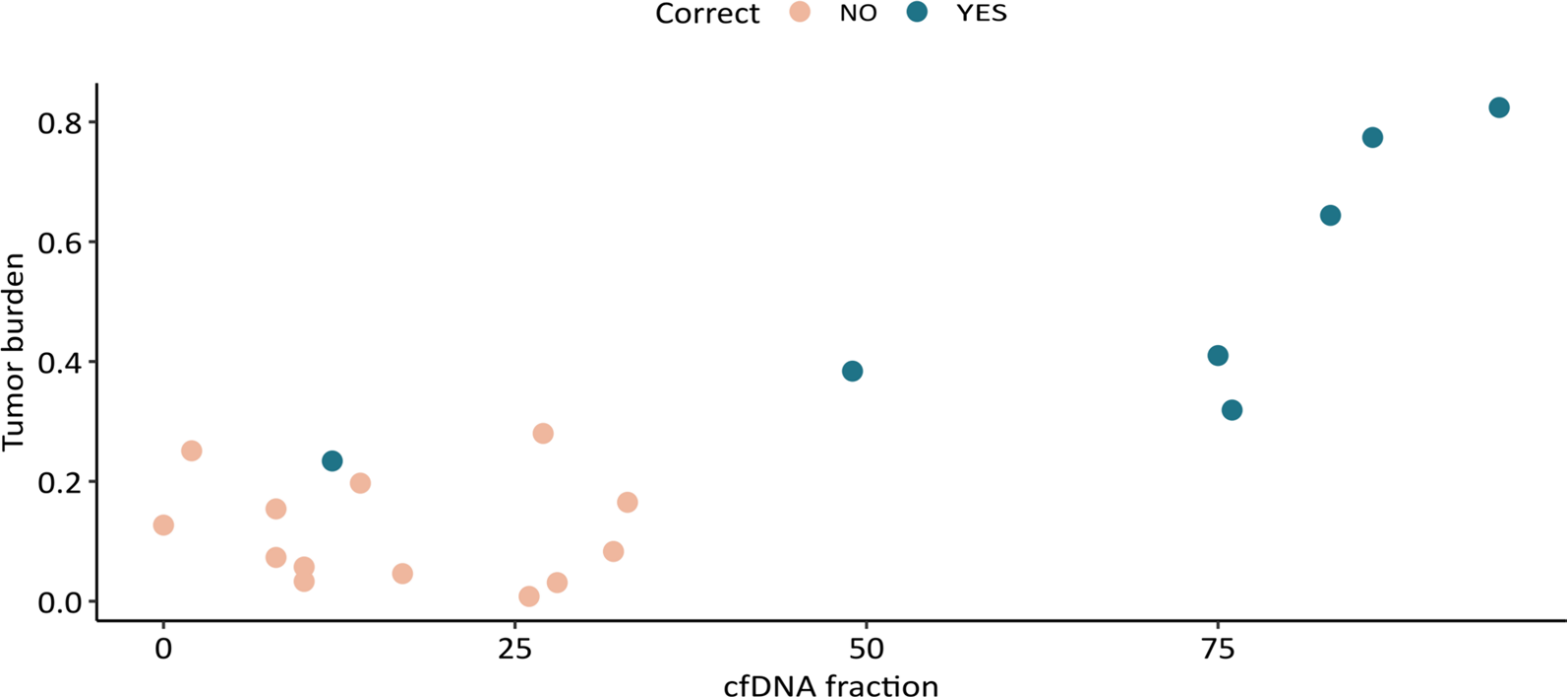
Plot of the cfDNA fraction based on length profiling before library preparation and the estimated tumor burden after deconvolution. Samples that score high on both these variables show a classification that corresponds with the pathological diagnosis. Correct classification in blue and incorrect classification in orange

We noted higher fractions of fragmented cfDNA on total DNA and a higher estimated tumor fraction according to deconvolution in samples that were correctly classified. Although the relatively small patient cohort does withhold us from defining validated cutoff values, we see that most samples with a cfDNA/total DNA fraction below 40% and/or an estimated tumor burden below 30% are classified incorrectly (13/14). Samples with cfDNA/total DNA fraction of at least 40% and tumor burden of at least 30% are all classified correctly (6/6), as illustrated in Fig. 4.

### Copy number profiling from cfRRBS data

**Fig. 5:**
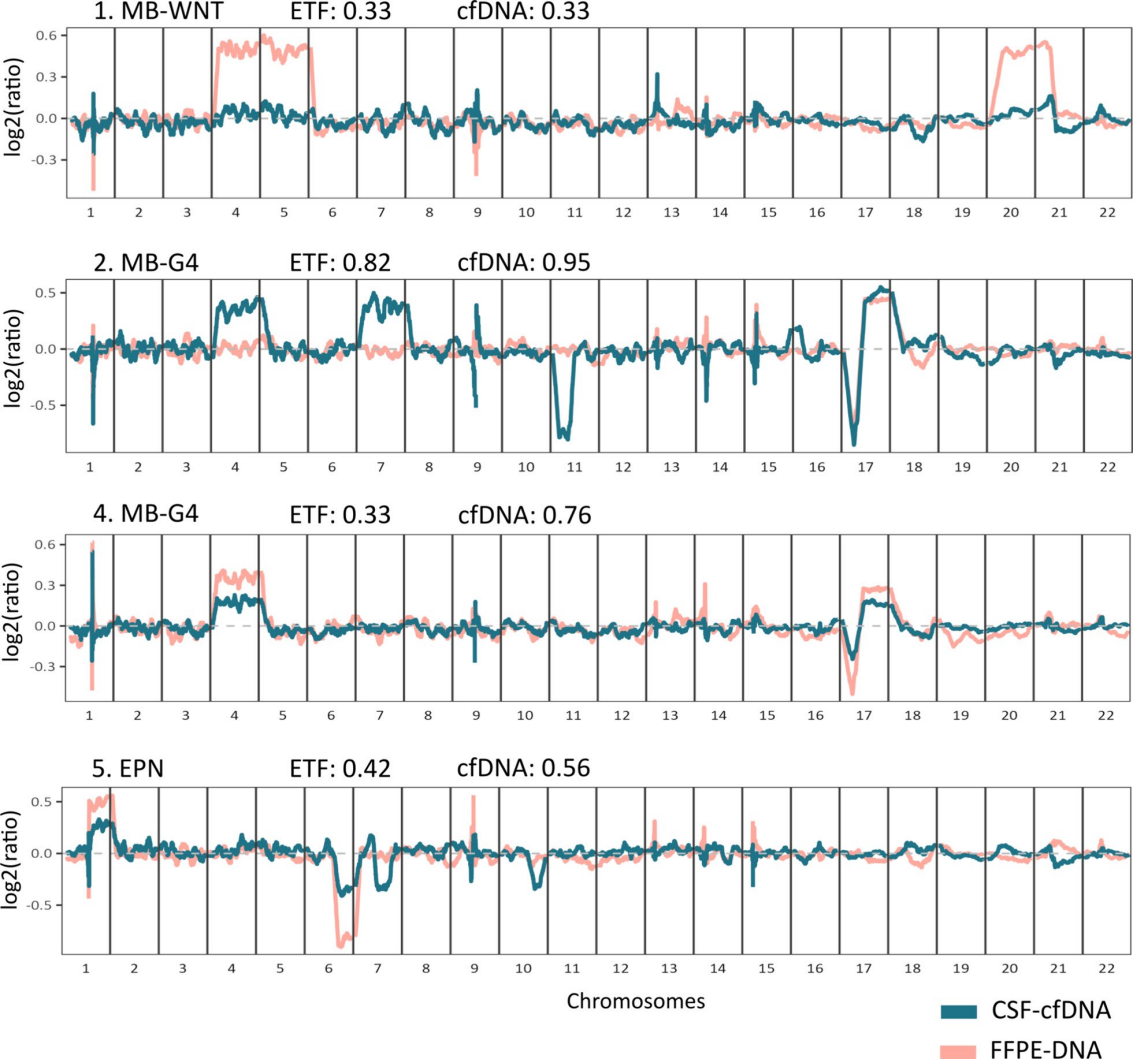
DNA copy number profiles of four included patients, with corresponding estimated tumor fractions (ETF) and cfDNA fraction (cfDNA) of the CSF-cfDNA. Overlapping profiles of the tumor formalin-fixed paraffin-embedded (FFPE) material in blue and CSF material in orange show both corresponding aberrations as well as some heterogeneity

Copy number profiles can be extracted from cfRRBS data. For 11 donors, we were able to compare copy number profiles from tumor DNA and cfDNA. Although data are more noisy compared to whole-genome sequencing methods, we observe copy number variations (CNVs) in 3 of these patients. For samples with lower estimated tumoral fractions, such as case 1 illustrated in Fig. 5, we could not identify any aberrations in the CSF-cfDNA. Case 5 shows aberrations that correlate well with the ones in the tumor tissue. Interestingly, for 2 of these patients (case 2 and case 5) we observed additional  CNVs in the liquid samples compared to the tumor suggestive for intratumoral heterogeneity.

## Discussion

In this proof-of-concept study, we present a workflow to classify pediatric CNS tumors using a DNA methylation assay that was developed for fragmented cfDNA isolated from liquid biopsies, and that works on both DNA from fresh frozen and paraffin-embedded tissues. Tumor type and tumor fraction are estimated using computational deconvolution based on a reference dataset containing published methylation data of brain tumor tissues complemented with healthy cfDNA profiles. We found that the tumor classification correlates well with histopathological diagnosis for good quality cfDNA samples. We identified several pitfalls of our approach related to CSF collection and CSF characteristics, as well as opportunities for improvement which require further validation on larger patient cohorts before clinical implementation.

In summary, 7 out of 20 samples from pediatric CNS cancer patients are classified correctly using cfDNA from CSF. All samples with high cfDNA/total DNA fraction were classified correctly (6/20). In most misclassified samples, we observe increased fractions of HMW-DNA (length over 700 bp) among the isolated DNA in CSF, with 14 samples showing a cfDNA fraction lower than 0.5. Additionally, more than half of the samples exhibit a cfDNA yield below 5 ng. The scarcity of cfDNA in CSF is not emphasized in similar studies, yet it is notable that published articles working on cfDNA from CSF for tumor classification and follow-up almost exclusively focus on higher grade tumors [36, 37, 44–50]. Although ctDNA levels are not defined by tumor grade alone, it is an important variable in the release of fragmented DNA [51]. Next to the more urgent clinical need for those more aggressive tumors, lack of studies on lower grade tumors most probably indicates the challenges in obtaining sufficient ctDNA (circulating tumoral DNA) material. The Afflerbach study [37], in contrast, does include low- and high-grade tumors, and indeed underscores the low abundance of ctDNA in the CSF. Due to minimal input requirements of 1 ml CSF and 5 ng of DNA, only 72% samples were suitable for nanopore sequencing. Of these samples 17% passed the minimal technical requirements for methylation profiling and correct classification. Although our cohort size prevents direct comparison, the cfRRBS approach confirms these results with successfully generated methylation profiles on all included samples and correctly estimated tumor diagnosis in 30% of the samples. Still, obtaining sufficient ctDNA appears challenging for lower grade tumors.

In the paragraphs below, we discuss the different factors that impact the performance of our classification approach, including HMW-DNA contamination, tumoral cfDNA fractions and reference dataset.

In previous cfRRBS studies, we have already highlighted the importance of assessing the cfDNA/HMW-DNA fraction in each sample [39]. HMW-DNA that is also processed in the cfRRBS library preparation will dilute the signal of the cfDNA and might lead to misclassification of samples in case of high fractions of HMW-DNA. The HMW-DNA is likely derived from cells that are damaged during ventricular drain placement or white blood cells in blood-contaminated samples. In this study, we show that centrifugation of a fresh CSF sample before DNA isolation improves sample quality in many cases. Freezing the CSF prior to centrifugation results in lysis of the cells and thus release of HMW-DNA into the fluid. Thus, centrifugation within four hours after collection and before freezing is ideal, but more challenging to implement into clinical practice. In addition, avoiding blood cell contamination will result in better quality samples. Secondly, although yield of ctDNA relates to variables such as tumor size and aggression [51], sampling in close proximity of the tumor through a ventricular drain might collect more ctDNA compared to sampling via lumbar puncture. These observations underscore the need for more dedicated studies investigating the pre-analytical variables that can improve the sample quality, as well as more standardized protocols for CSF collection ensuring the standard collection of high-quality samples allowing robust tumor classification. The significance of this is highlighted by the wide variation in published collection protocols, encompassing collection through lumbar puncture, ventricular drainage, or mixed cohorts, with volumes ranging from 200 µl to 10 ml. While centrifugation is commonly included, there are studies that omit this step [30, 31, 33–35, 37, 45].

The absence of a well-defined profile characterizing the non-tumoral background in cerebrospinal fluid (CSF) poses a limitation in accurately estimating the fraction of a specific tumor type. This challenge becomes particularly pronounced in cases where CSF samples contain lower tumor fractions and higher non-tumoral cfDNA. We observe that in the samples with cfDNA fractions below 50%, most samples are classified as central neurocytoma or infantile hemispheric glioma. We observed a better classification accuracy in cfDNA samples with higher estimated tumor fractions. In blood samples with lower tumor fraction, the non-tumoral cfDNA mostly originate from the white blood cells [42]. For CSF, however, the origin of the non-tumoral cfDNA is not clearly defined and might originate from WBC, but also from brain tissue damaged due to increased pressure in patients presenting with hydrocephalus. In the CSF of 4 low-grade cancer patients, central neurocytoma (CN) is the highest estimated tumor signal. This might be explained by ventricular cells that are damaged due to the increased intracranial pressure, that and resemble the methylation profile of the ventricular CN tumor. Similar effects might be present for infantile hemispheric glioma (IHG), highest estimated tumor fraction in 7/20 patients. To investigate this hypothesis, we need CSF samples of non-tumoral pediatric patients with increased intracranial pressure which is very difficult to obtain.

Although the Capper reference data include various healthy brain tissues, pediatric profiles often differ from adult. Additionally, the increased intracranial pressure does not match a physiological state, and hydrocephalus background profiles might be interpreted as tumor entities. To allow proper deconvolution of all the contributing cell types in the CSF-cfDNA samples, a reference dataset encompassing all those cell types is necessary. However, pediatric CSF collection is only performed in patients with a (suspected) brain-related pathology, thus obtaining a reference sample and DNA methylation profile from pediatric hydrocephalus patients without brain pathologies is almost impossible. One option would be the inclusion of CSF from patients with hydrocephalus caused by a traumatic brain injury.

The performance of deconvolution algorithms heavily relies on the choice of reference data. The DNA methylation-based assay for CNS brain tumor diagnosis is utilized in an increasing number of pathological departments and employs the Infinium HumanMethylation450 BeadChip array [11]. This array encompasses 450,000 methylation sites and shows good performance to distinguish between different tumor entities. However, a drawback is the recommended input of 500 ng DNA [52], a quantity significantly surpassing the average cfDNA yield from liquid biopsies. To address this challenge, we successfully employed cell-free reduced representation bisulfite sequencing (cfRRBS), an approach tailored for low quantities of highly fragmented DNA, requiring only 10 ng or even less input DNA to generate high-quality DNA methylation profiles [38]. We formatted the published 450 K array methylation data [11] of CNS tumors to align with the cfRRBS workflow and used it as a reference dataset for deconvolution. A limitation of this approach is that we only use the sites that are covered by both the 450 K array and the cfRRBS assay, which is only 13.7% of methylation sites that are covered by the cfRRBS assay. By restricting the number of sites, we noticed that discriminating low-grade glioma tumors became more challenging as visualized in the UMAP plot (Fig. 1) compared to the published UMAP [11]. Building a (cf)RRBS-based reference dataset would enable the utilization of all cfRRBS regions in the deconvolution model and thus increase the available information to discriminate different tumor entities; however, this will come with additional effort and costs. This problem highlights the trade-off between maximizing data inclusivity and managing data availability or associated financial constraints. In addition to the restricted number of sites, the published version of the classifier shows challenges in discriminating low-grade glioma tumors, resulting in less accurate predictions for this particular subtype [53]. Newer versions of the classifier can improve classification for several challenging tumor types including low-grade gliomas; however, the reference data of newer versions are not publicly available [53].

Interestingly, the data produced via cfRRBS can also be used for CNV profiling. Although these data are more noisy compared to dedicated CNV profiling assays such as shallow whole-genome sequencing (shWGS), extraction of multiple data layers from cfRRBS reads without requiring new input material is an important asset. Compared to most cfDNA shWGS approaches for CNV analysis, cfRRBS lacks a size separation step, and thus, also HMW-DNA will be processed [38] resulting in a dilution of the tumoral signal. Indeed, for samples with tumoral fraction below 30% we could not observe any tumor associated aberrations. For the patients with matched tumor and CSF material and higher estimated tumor fractions, we observed some CSF-specific aberrations suggesting intratumoral heterogeneity, similar to the results described by Chicard et al. [44]. However, it is notable that the lower quality of the CNV profile data limits the number of patients for which the CNV profile can accurately be analyzed (Figs. 5, supplementary Fig. 2).

An important factor to consider for clinical implementation of an assay is the time between sampling and reporting of results. For the proposed assay (cfRRBS followed by computational analysis), the turnaround time is roughly 5 days in an optimized setting where samples are processed immediately after collection. This is a reasonable turnaround time for molecular diagnostics and falls perfectly within the median turnaround time of 21 days that are presented for most targeted NGS and DNA methylation profiling assays [54]. Another important advantage is that the proposed workflow is designed almost completely as a single tube reaction which facilitates clinical implementation through a fully automated liquid handling system [38]. Additionally, the cfRRBS protocol is cost-effective compared to other sequencing methods. By targeting particular subsections of the genome, sufficient sequencing coverage is achieved using only 20–25 M reads per sample [38].

## Conclusion

Although the presented cfRRBS approach on CSF has several limitations that need further optimization, we believe that this approach can become a valuable alternative for cancer patients with tumors located in regions that are too delicate for a surgical biopsy. Validation on larger cohorts is required, still we observed accurate classification for patients with cfDNA fractions higher than 50%. We expect that methylation profiling of cfDNA isolated from liquid biopsies could take an important and complementary position next to standard diagnostic approaches, for example by giving an early diagnosis that can inform oncologists and surgeons in their choice for a treatment strategy.

### Supplementary Information

Additional file 1.

Additional file 2.

Additional file 3.

Additional file 4.

Additional file 5.

Additional file 6.

Additional file 7.

## Data Availability

The fastq files of the cfRRBS data of the CSF and tumor samples within this study are available on EGA (reference ID EGAD50000000554). Fastq files of the control cfDNA, isolated from plasma from healthy adults is available on EGA (reference ID EGAD50000000553).

## Abbreviations

CSF: Cerebrospinal fluids
cfDNA: Circulating cell-free DNA
CNS: Central nervous system
WHO: World Health Organization
cfRNA: Circulating cell-free RNA
ctDNA: Circulating tumor DNA
WGBS: Whole-genome bisulfite sequencing
CSF-cfDNA: Cerebrospinal fluid cell-free DNA
cfRRBS: Cell-free reduced representation bisulfite sequencing
HMW-DNA: High molecular weight DNA
WBC: White blood cells
LGG: Low-grade glioma
MB: Medulloblastoma
EPN: Ependymoma
PLEX: Choroid plexus papilloma
LGG-PA: Pilocytic astrocytoma
ATRT: Atypical teratoid rhabdoid tumor
CPH: Adamantinomatous craniopharyngioma
DMG: Diffuse midline glioma
FFPE: Formalin-fixed paraffin-embedded
CNV: Copy number variation
shWGS: Shallow whole-genome sequencing
ETF: Estimated tumor fraction
