# Supplementary material for: Diagnosis of pediatric central nervous system tumors using methylation profiling of cfDNA from cerebrospinal fluid
Source: Clin Epigenetics. 2024 Jul 5;16:87. doi: 10.1186/s13148-024-01696-w (PMC11225235; doi:10.1186/s13148-024-01696-w)
Supplement: Supplementary file 1 — Additional file 1. [file 13148_2024_1696_MOESM1_ESM.pdf]

A1: Ladder

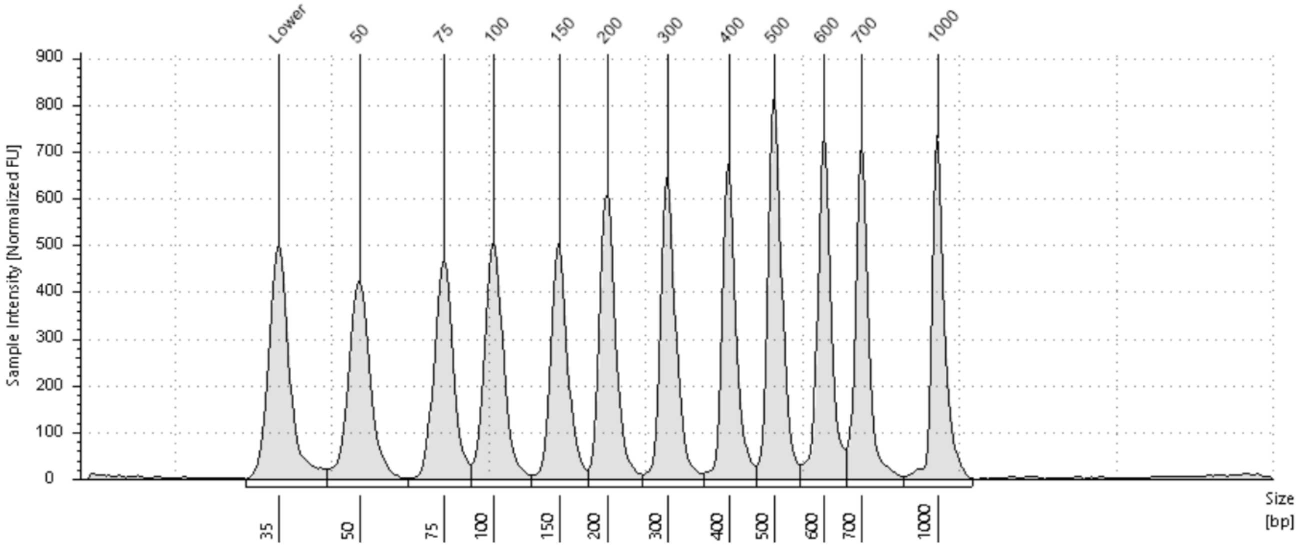

Sample Table

Well	%cfDNA	Conc. [pg/µl]	Sample Description	Alert	Observations
A1	-	2800	Ladder		Ladder

Peak Table

Size [bp]	Calibrated Conc. [pg/µl]	Assigned Conc. [pg/µl]	Peak Molarity [pmol/l]	% Integrated Area	Height	Peak Comment	Observations
35	275	275	12100	-	76.345		Lower Marker
50	239	-	7350	8.57	64.295		
75	240	-	4920	8.61	71.576		
100	251	-	3860	9.02	77.214		
150	226	-	2320	8.11	76.874		
200	253	-	1950	9.09	93.472		
300	260	-	1330	9.33	98.713		
400	254	-	975	9.10	103.100		
500	300	-	924	10.78	125.306		
600	265	-	680	9.53	113.059		
700	249	-	548	8.95	109.958		
1000	248	-	382	8.92	112.277		

© Agilent Technologies, Inc. 2019

B1: DNA054191 plastic_nocentrifugation_D1

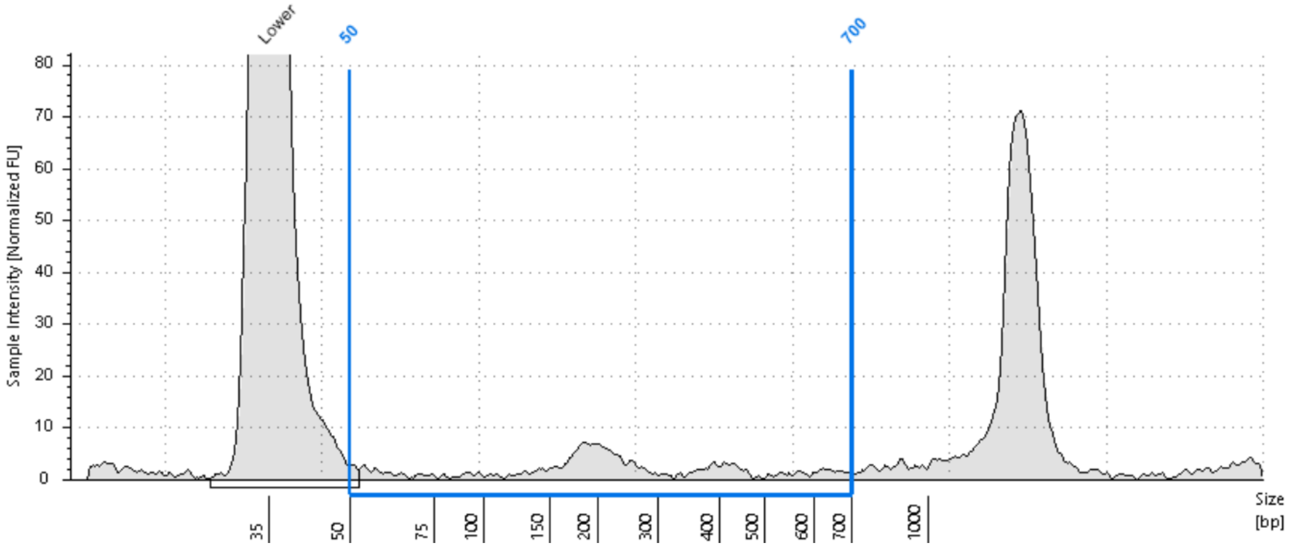

Sample Table

Well	%cfDNA	Conc. [pg/µl]	Sample Description	Alert	Observations
B1	19	78.1	DNA054191	⚠	Sample concentration outside functional range for %cfDNA and the assay

Region Table

From [bp]	To [bp]	Average Size [bp]	Conc. [pg/µl]	Region Molarity [pmol/l]	% of Total	Region Comment	Color
50	700	265	14.6	171	18.71	%cfDNA	■

© Agilent Technologies, Inc. 2019

C1: DNA054192 plastic_centrifugation_supernatant_D1

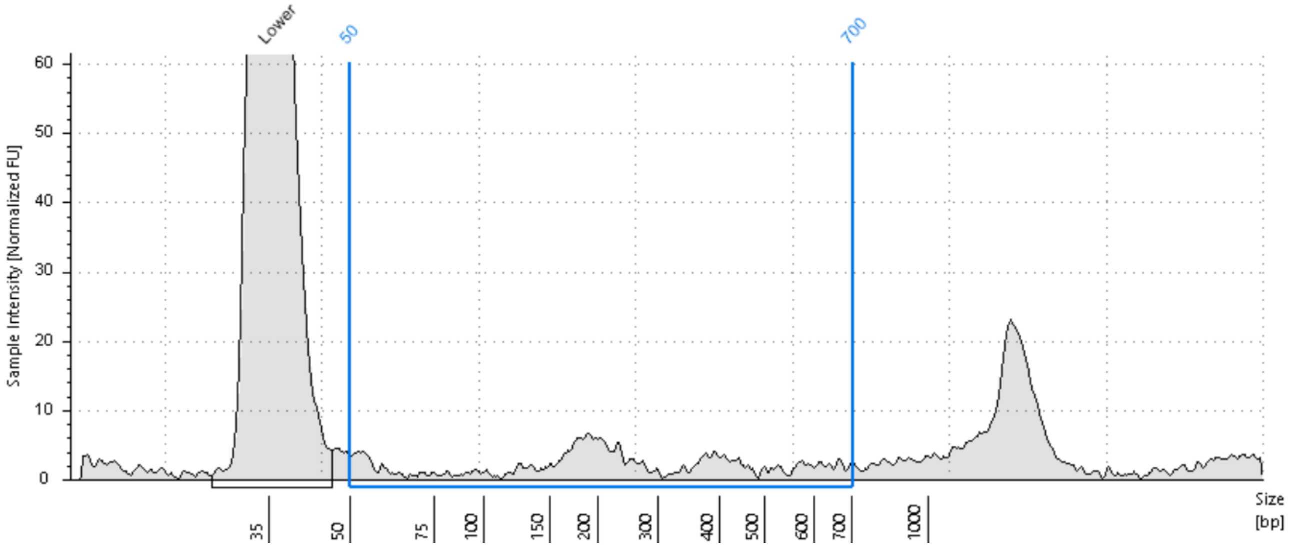

Sample Table

Well	%cfDNA	Conc. [pg/µl]	Sample Description	Alert	Observations
C1	-	47.8	DNA054192	⚠	Sample concentration outside functional range for %cfDNA and the assay

Region Table

From [bp]	To [bp]	Average Size [bp]	Conc. [pg/µl]	Region Molarity [pmol/l]	% of Total	Region Comment	Color
50	700	276	15.9	196	33.38	%cfDNA	

© Agilent Technologies, Inc. 2019

F1: DNA054197 plastic_nocentrifugation_D2

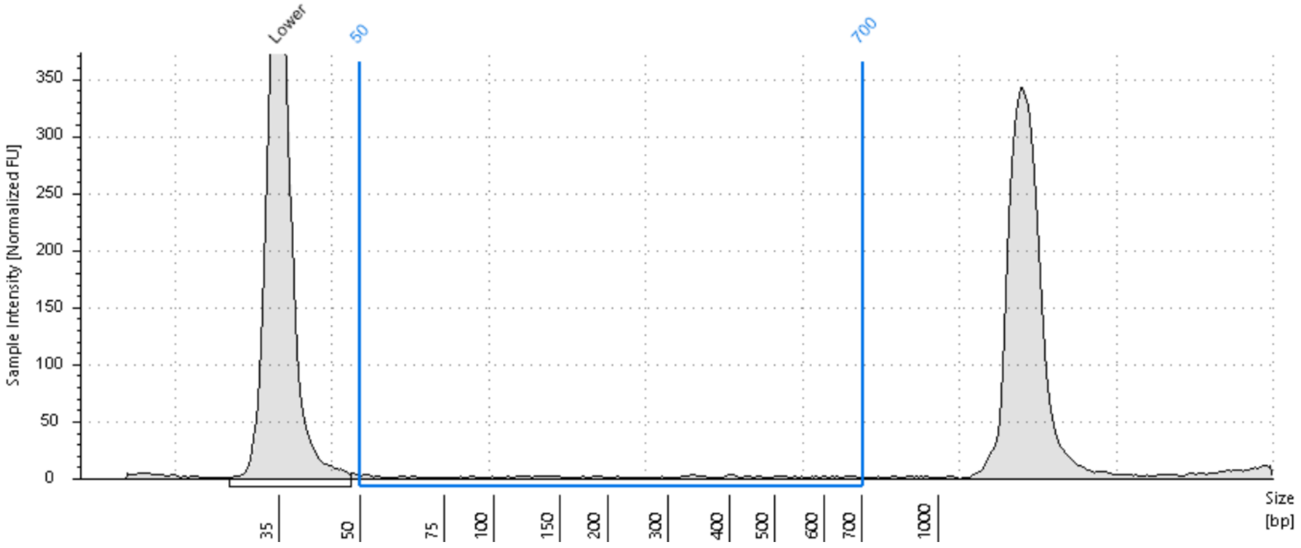

Sample Table

Well	%cfDNA	Conc. [pg/µl]	Sample Description	Alert	Observations
F1	2	275	DNA054197		

Region Table

From [bp]	To [bp]	Average Size [bp]	Conc. [pg/µl]	Region Molarity [pmol/l]	% of Total	Region Comment	Color
50	700	277	5.11	123	1.86	%cfDNA	

© Agilent Technologies, Inc. 2019

G1: DNA054198 plastic_centrifugation_supernatant_D2

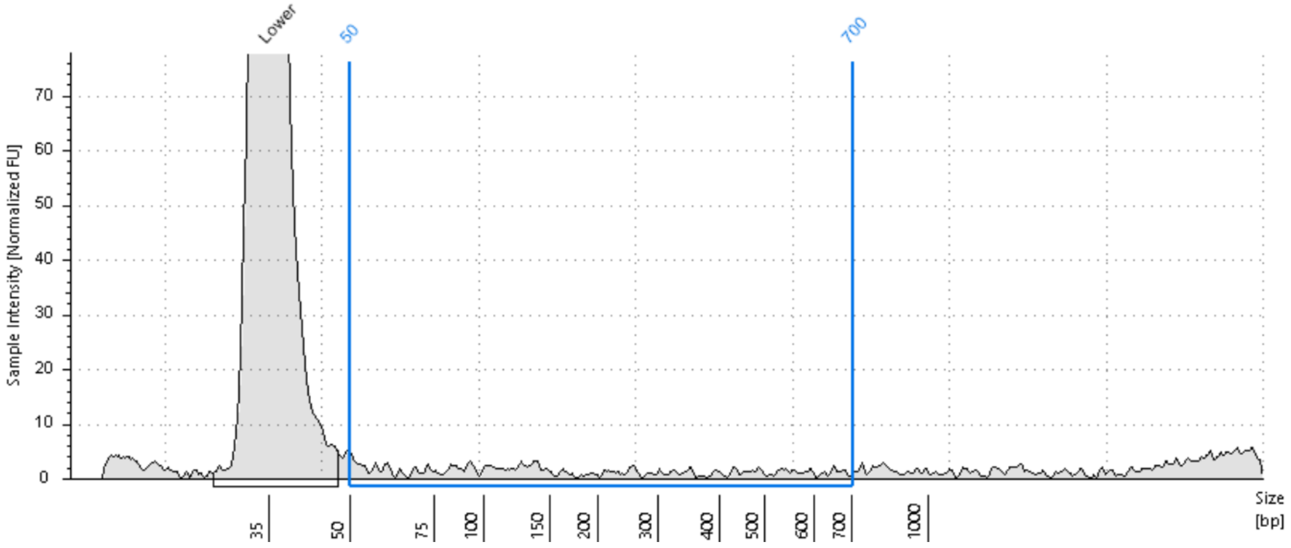

Sample Table

Well	%cfDNA	Conc. [pg/µl]	Sample Description	Alert	Observations
G1	-	16.1	DNA054198	⚠	Sample concentration outside functional range for %cfDNA and the assay

Region Table

From [bp]	To [bp]	Average Size [bp]	Conc. [pg/µl]	Region Molarity [pmol/l]	% of Total	Region Comment	Color
50	700	235	5.84	169	36.19	%cfDNA	

© Agilent Technologies, Inc. 2019

B2: DNA054203 plastic_nocentrifugation_D3

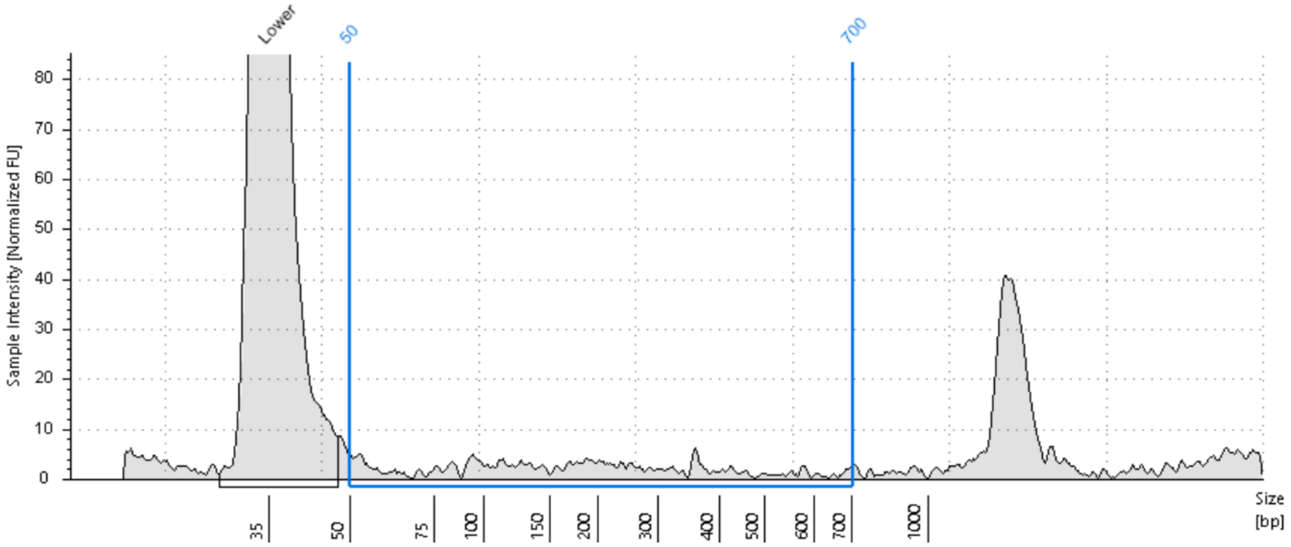

Sample Table

Well	%cfDNA	Conc. [pg/µl]	Sample Description	Alert	Observations
B2	27	56.6	DNA054203	⚠	Sample concentration outside functional range for %cfDNA and the assay

Region Table

From [bp]	To [bp]	Average Size [bp]	Conc. [pg/µl]	Region Molarity [pmol/l]	% of Total	Region Comment	Color
50	700	221	15.3	234	27.02	%cfDNA	

© Agilent Technologies, Inc. 2019

C2: DNA054204 plastic_centrifugation_supernatant_D3

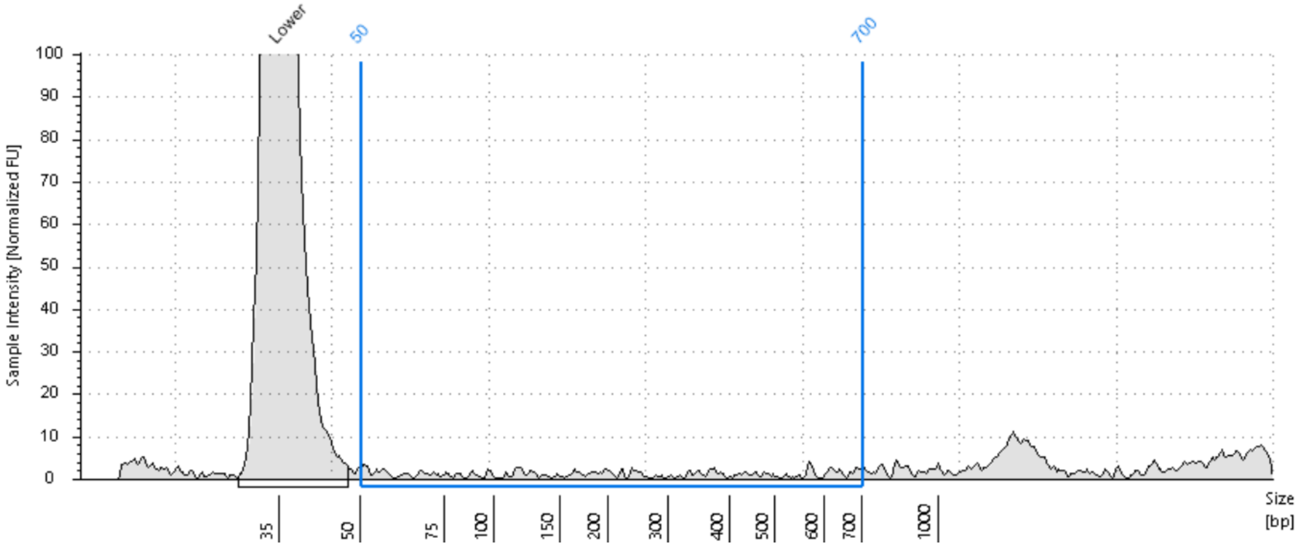

Sample Table

Well	%cfDNA	Conc. [pg/µl]	Sample Description	Alert	Observations
C2	-	28.7	DNA054204	⚠	Sample concentration outside functional range for %cfDNA and the assay

Region Table

From [bp]	To [bp]	Average Size [bp]	Conc. [pg/µl]	Region Molarity [pmol/l]	% of Total	Region Comment	Color
50	700	277	5.61	127	19.50	%cfDNA	■

© Agilent Technologies, Inc. 2019

F2: DNA054209 plastic_nocentrifugation_D4

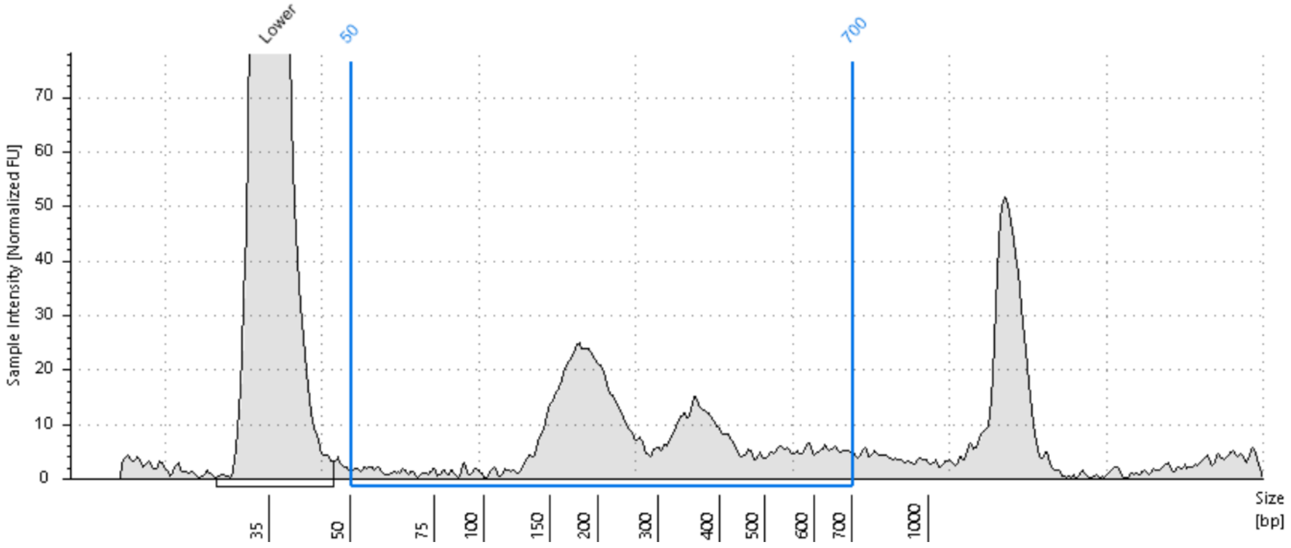

Sample Table

Well	%cfDNA	Conc. [pg/µl]	Sample Description	Alert	Observations
F2	56	116	DNA054209		

Region Table

From [bp]	To [bp]	Average Size [bp]	Conc. [pg/µl]	Region Molarity [pmol/l]	% of Total	Region Comment	Color
50	700	288	65.5	491	56.45	%cfDNA	

© Agilent Technologies, Inc. 2019

G2: DNA054210 plastic_centrifugation_supernatant_D4

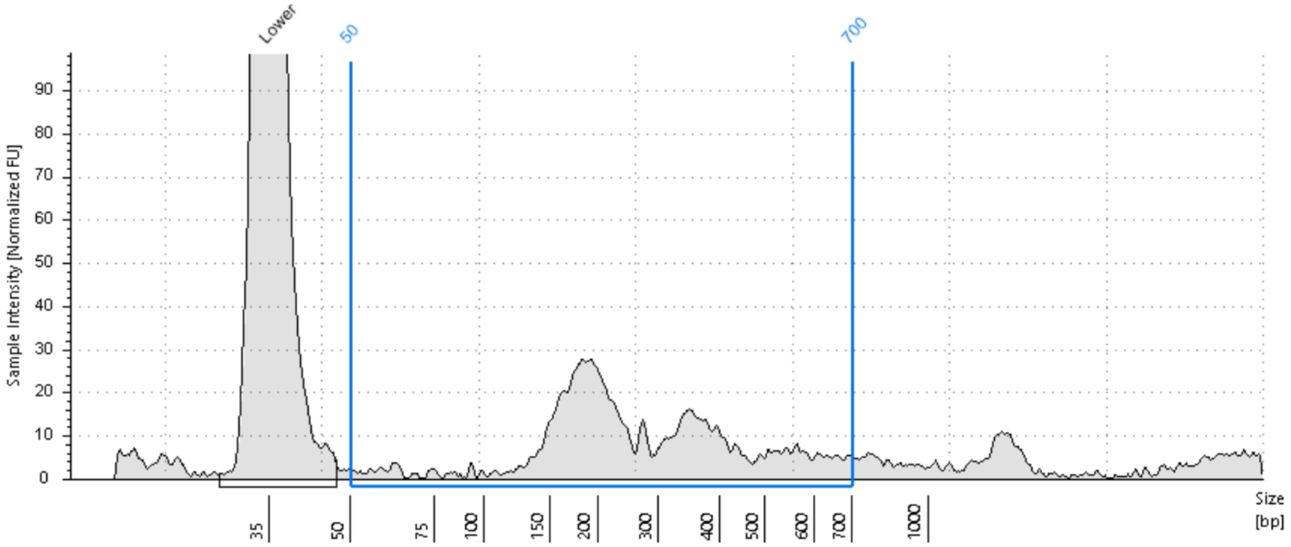

Sample Table

Well	%cfDNA	Conc. [pg/µl]	Sample Description	Alert	Observations
G2	75	97.2	DNA054210	⚠	Sample concentration outside functional range for %cfDNA and the assay

Region Table

From [bp]	To [bp]	Average Size [bp]	Conc. [pg/µl]	Region Molarity [pmol/l]	% of Total	Region Comment	Color
50	700	288	72.9	571	74.99	%cfDNA	

© Agilent Technologies, Inc. 2019

B1: DNA055007 D5_plastic_no centrifugation

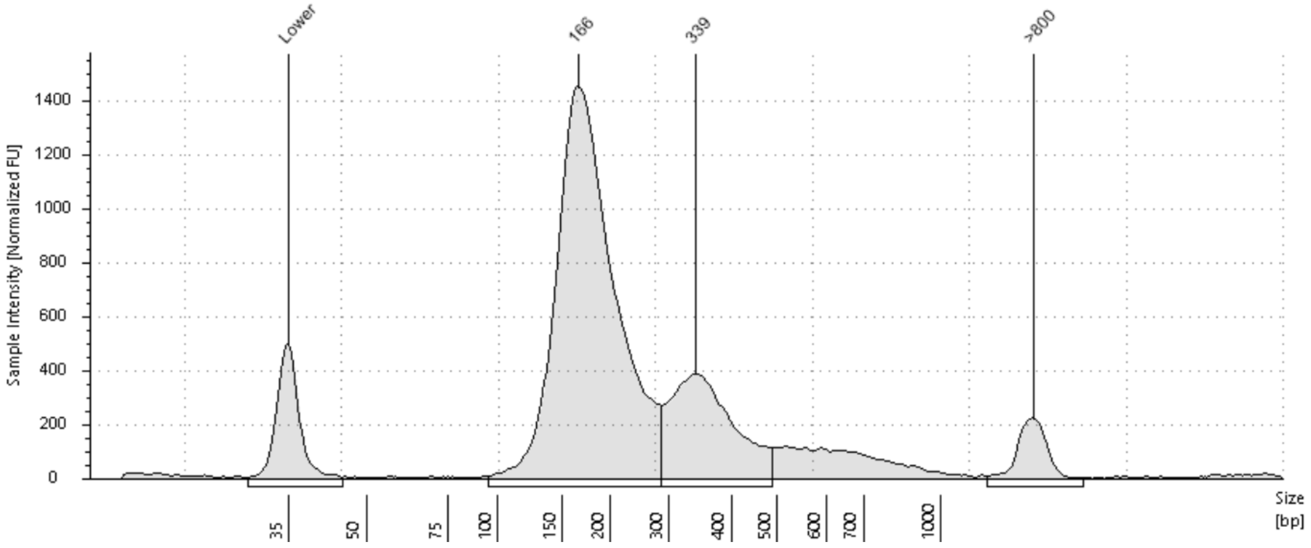

Sample Table

Well	%cfDNA	Conc. [pg/µl]	Sample Description	Alert	Observations
B1	91	3130	DNA055007	⚠	Caution! Expired ScreenTape device (used after two weeks of first use)

Peak Table

Size [bp]	Calibrated Conc. [pg/µl]	Assigned Conc. [pg/µl]	Peak Molarity [pmol/l]	% Integrated Area	Height	Peak Comment	Observations
35	275	275	12100	-	40.283		Lower Marker
166	2060	-	19100	73.17	116.852		
339	602	-	2730	21.35	31.300		
>800	154	-	-	5.48	17.914		

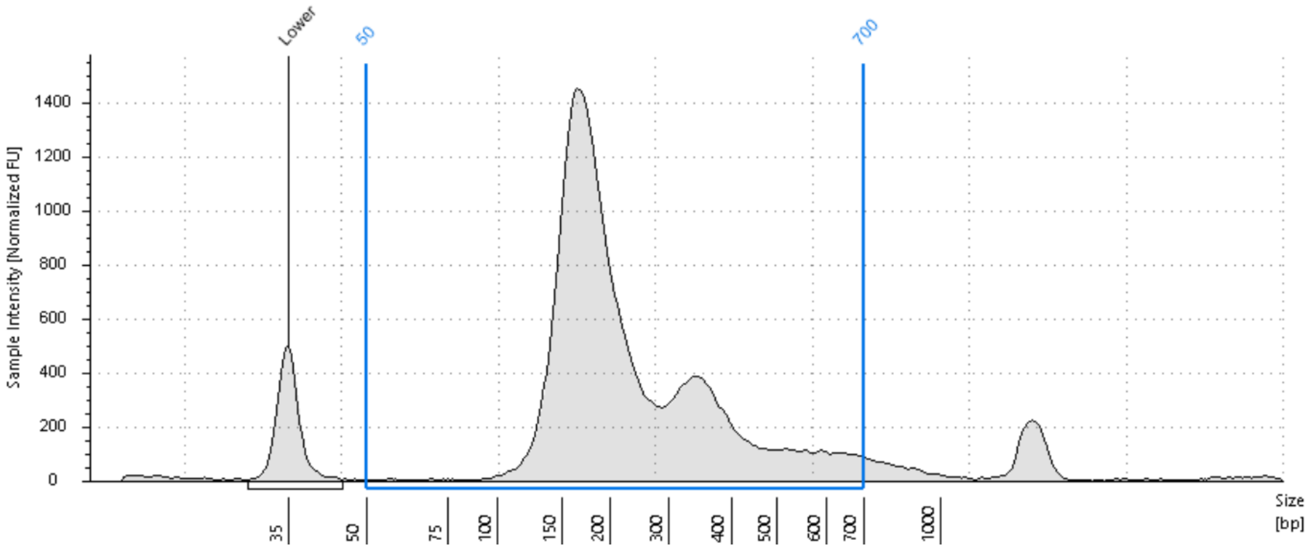

Region Table

From [bp]	To [bp]	Average Size [bp]	Conc. [pg/µl]	Region Molarity [pmol/l]	% of Total	Region Comment	Color
50	700	247	2860	21500	91.37	%cfDNA	■

© Agilent Technologies, Inc. 2019

C1: DNA055008 D5_plastic_centrifugation

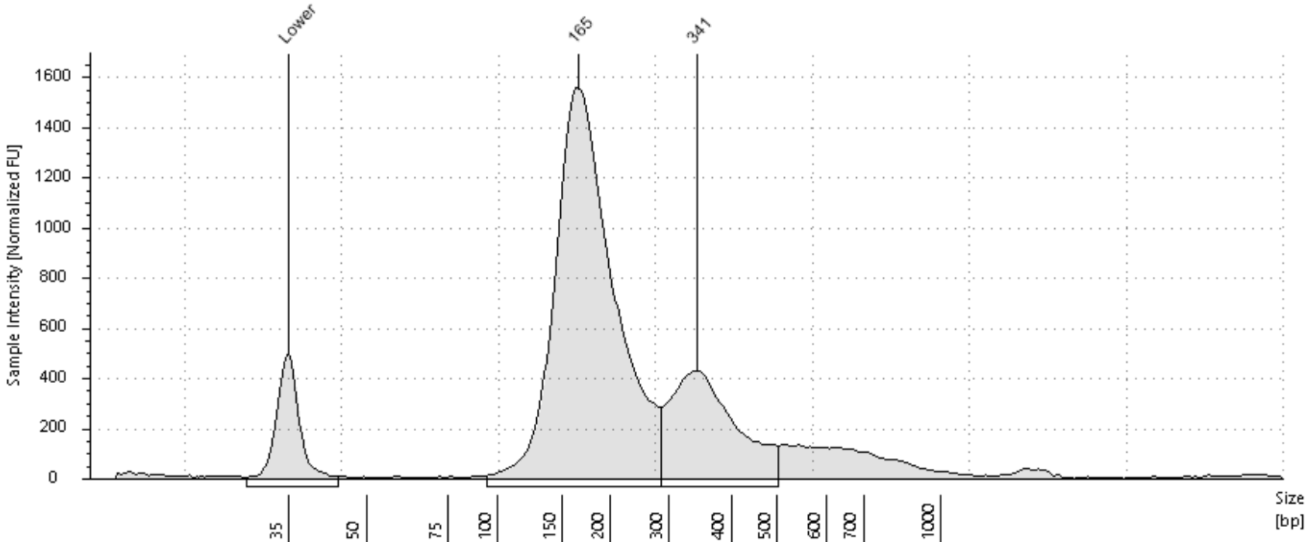

Sample Table

Well	%cfDNA	Conc. [pg/µl]	Sample Description	Alert	Observations
C1	95	3350	DNA055008	⚠	Caution! Expired ScreenTape device (used after two weeks of first use)

Peak Table

Size [bp]	Calibrated Conc. [pg/µl]	Assigned Conc. [pg/µl]	Peak Molarity [pmol/l]	% Integrated Area	Height	Peak Comment	Observations
35	275	275	12100	-	41.122		Lower Marker
165	2260	-	21000	76.31	128.291		
341	701	-	3160	23.69	35.379		

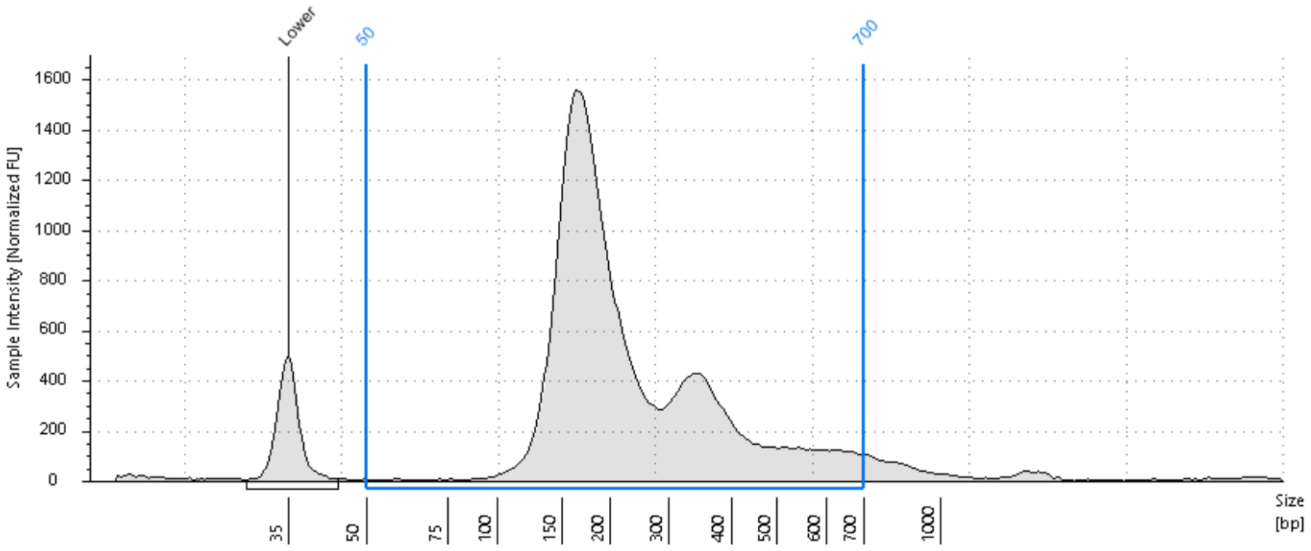

Region Table

From [bp]	To [bp]	Average Size [bp]	Conc. [pg/µl]	Region Molarity [pmol/l]	% of Total	Region Comment	Color
50	700	249	3180	23900	94.94	%cfDNA	■

© Agilent Technologies, Inc. 2019

F1: DNA055013 - D6_plastic_no centrifugation

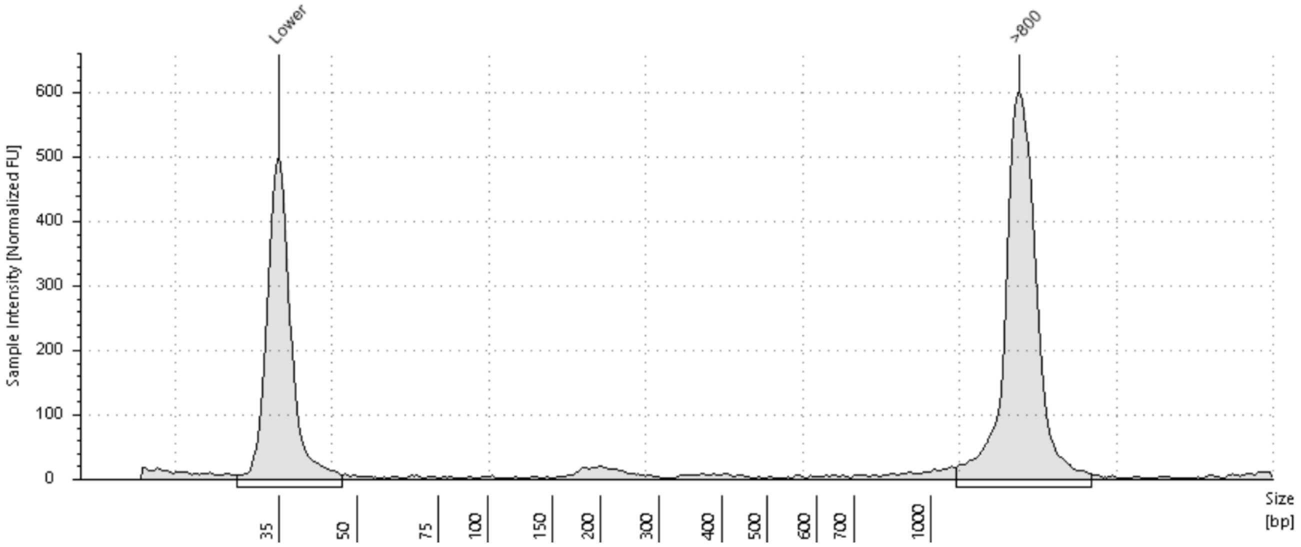

Sample Table

Well	%cfDNA	Conc. [pg/µl]	Sample Description	Alert	Observations
F1	7	494	DNA055013	⚠	Caution! Expired ScreenTape device (used after two weeks of first use)

Peak Table

Size [bp]	Calibrated Conc. [pg/µl]	Assigned Conc. [pg/µl]	Peak Molarity [pmol/l]	% Integrated Area	Height	Peak Comment	Observations
35	275	275	12100	-	69.675		Lower Marker
>800	432	-	-	100.00	83.759		

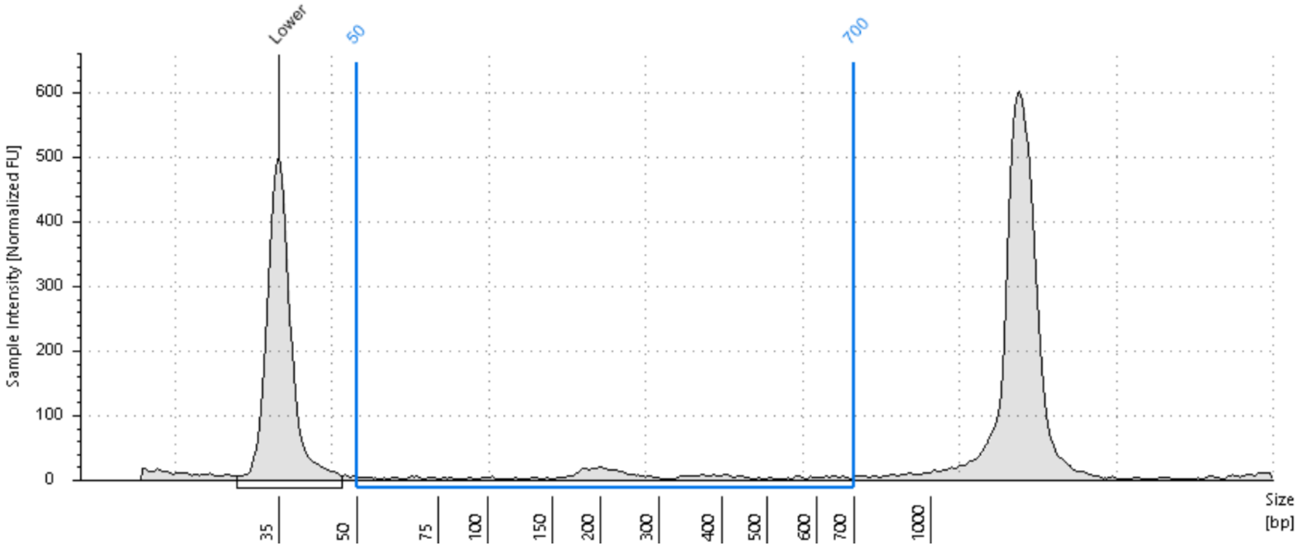

Region Table

From [bp]	To [bp]	Average Size [bp]	Conc. [pg/µl]	Region Molarity [pmol/l]	% of Total	Region Comment	Color
50	700	272	34.3	405	6.95	%cfDNA	

G1: DNA055014 - D6_plastic_centrifugation

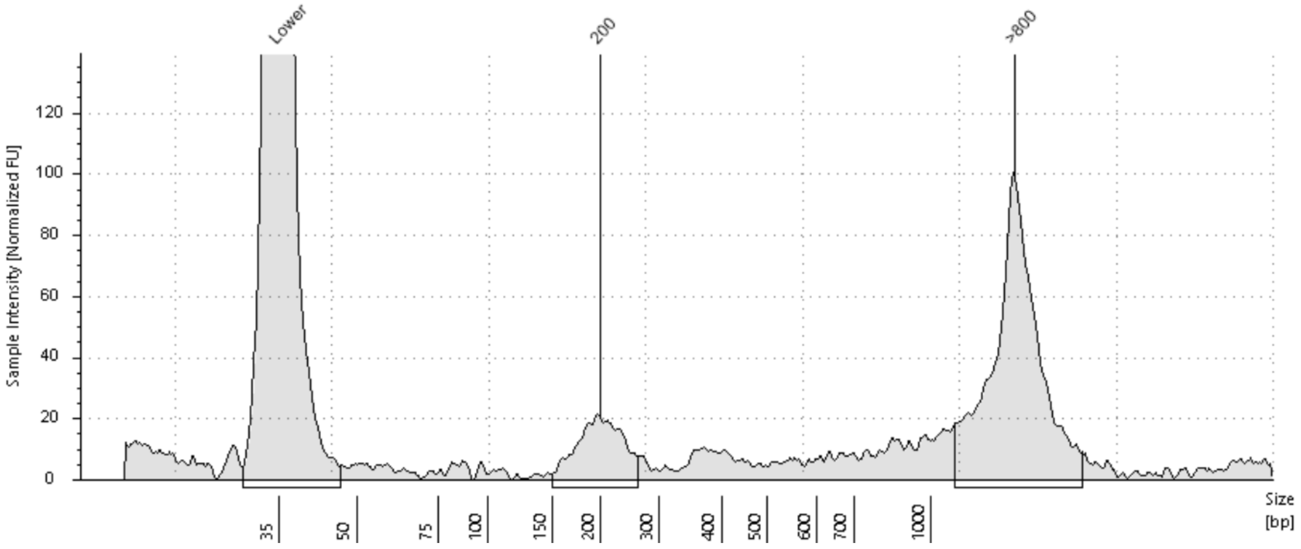

Sample Table

Well	%cfDNA	Conc. [pg/µl]	Sample Description	Alert	Observations
G1	28	182	DNA055014	⚠	Caution! Expired ScreenTape device (used after two weeks of first use)

Peak Table

Size [bp]	Calibrated Conc. [pg/µl]	Assigned Conc. [pg/µl]	Peak Molarity [pmol/l]	% Integrated Area	Height	Peak Comment	Observations
35	275	275	12100	-	94.408		Lower Marker
200	21.1	-	162	17.70	3.976		
>800	98.1	-	-	82.30	18.933		

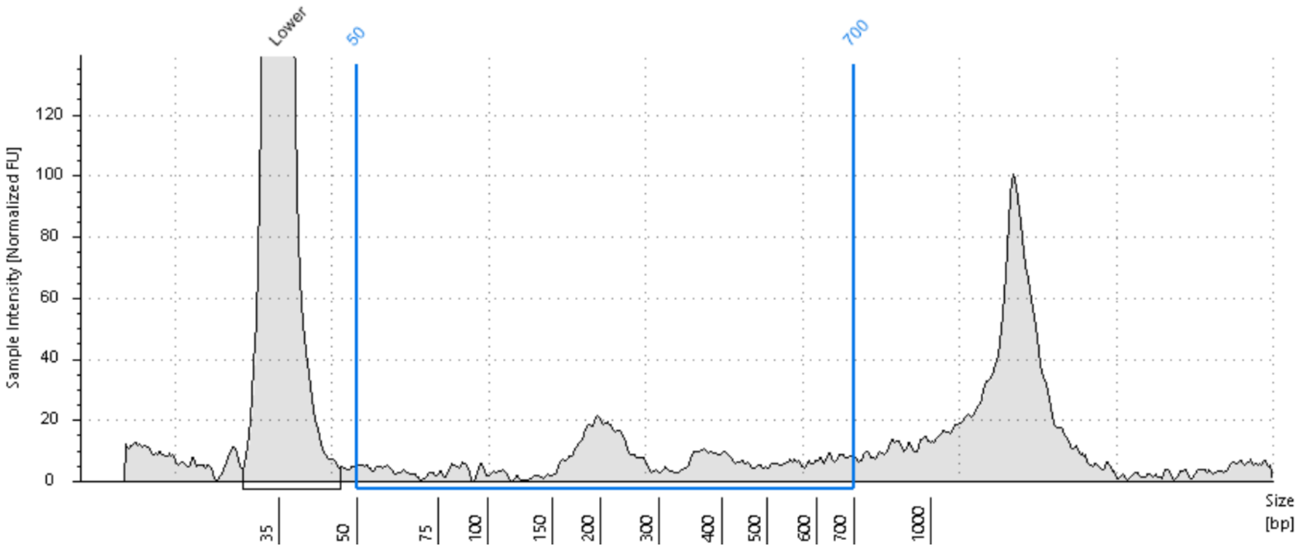

Region Table

From [bp]	To [bp]	Average Size [bp]	Conc. [pg/µl]	Region Molarity [pmol/l]	% of Total	Region Comment	Color
50	700	303	51.6	507	28.28	%cfDNA	■

© Agilent Technologies, Inc. 2019

H1: DNA055013 D6_plastic_no centrifugation Rest alliquot 1

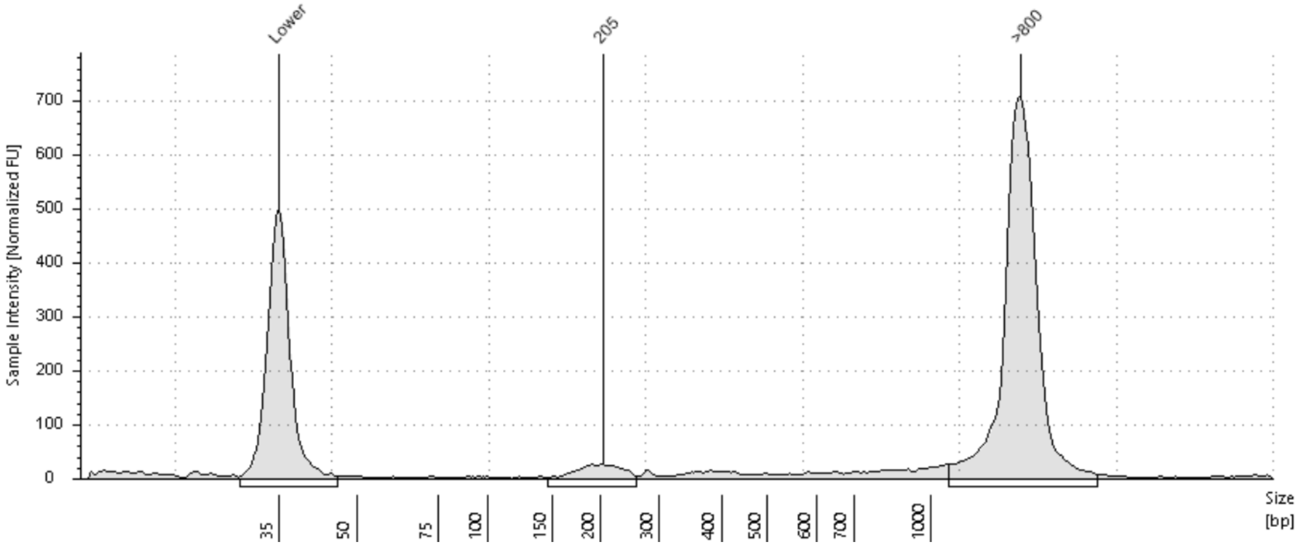

Sample Table

Well	%cfDNA	Conc. [pg/µl]	Sample Description	Alert	Observations
H1	10	655	DNA055013 R1	⚠	Caution! Expired ScreenTape device (used after two weeks of first use)

Peak Table

Size [bp]	Calibrated Conc. [pg/µl]	Assigned Conc. [pg/µl]	Peak Molarity [pmol/l]	% Integrated Area	Height	Peak Comment	Observations
35	275	275	12100	-	78.126		Lower Marker
205	26.7	-	200	4.61	4.133		
>800	552	-	-	95.39	110.802		

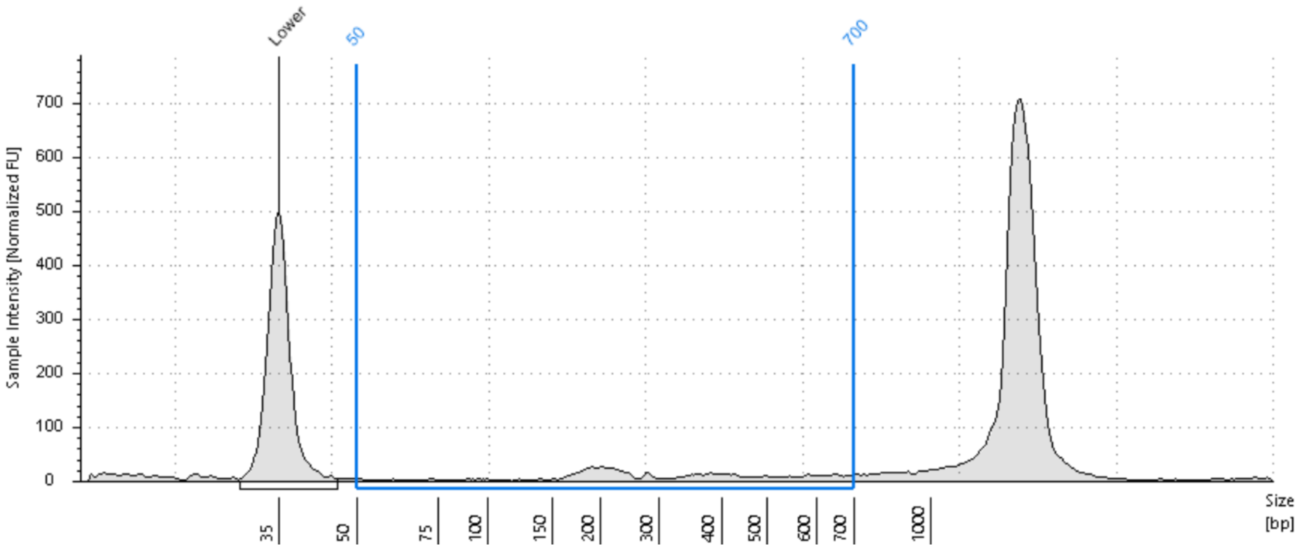

Region Table

From [bp]	To [bp]	Average Size [bp]	Conc. [pg/µl]	Region Molarity [pmol/l]	% of Total	Region Comment	Color
50	700	331	65.0	515	9.93	%cfDNA	■

© Agilent Technologies, Inc. 2019

A2: DNA055013 - D6_plastic_no centrifugation Rest alliquot 2

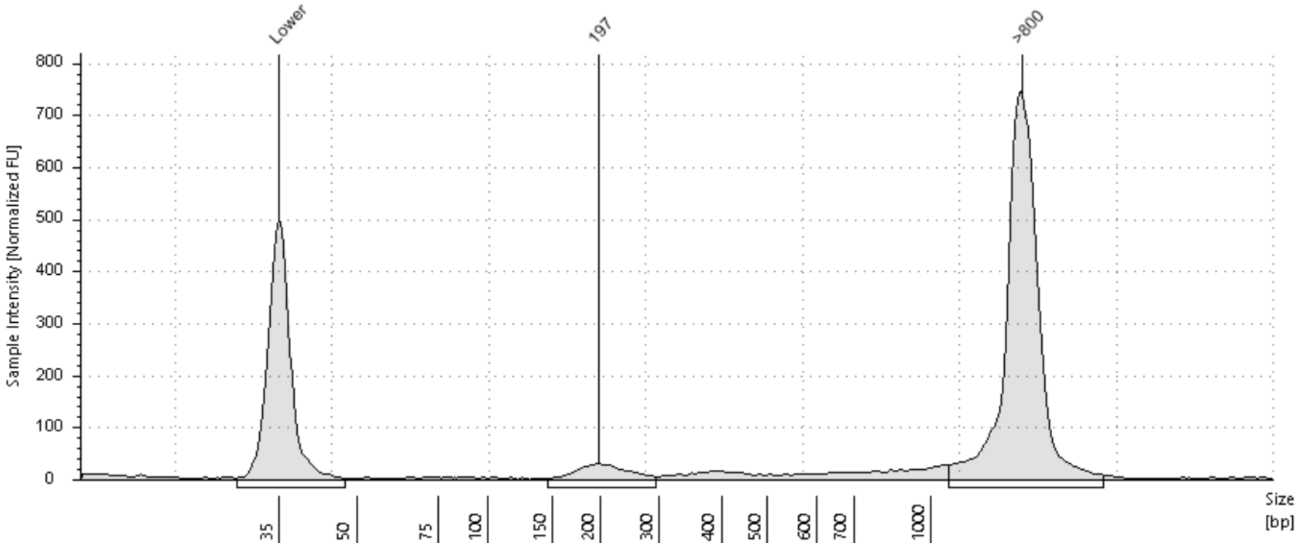

Sample Table

Well	%cfDNA	Conc. [pg/µl]	Sample Description	Alert	Observations
A2	12	699	DNA055013 R2	⚠	Caution! Expired ScreenTape device (used after two weeks of first use)

Peak Table

Size [bp]	Calibrated Conc. [pg/µl]	Assigned Conc. [pg/µl]	Peak Molarity [pmol/l]	% Integrated Area	Height	Peak Comment	Observations
35	275	275	12100	-	97.282		Lower Marker
197	34.8	-	271	5.70	6.014		
>800	575	-	-	94.30	144.891		

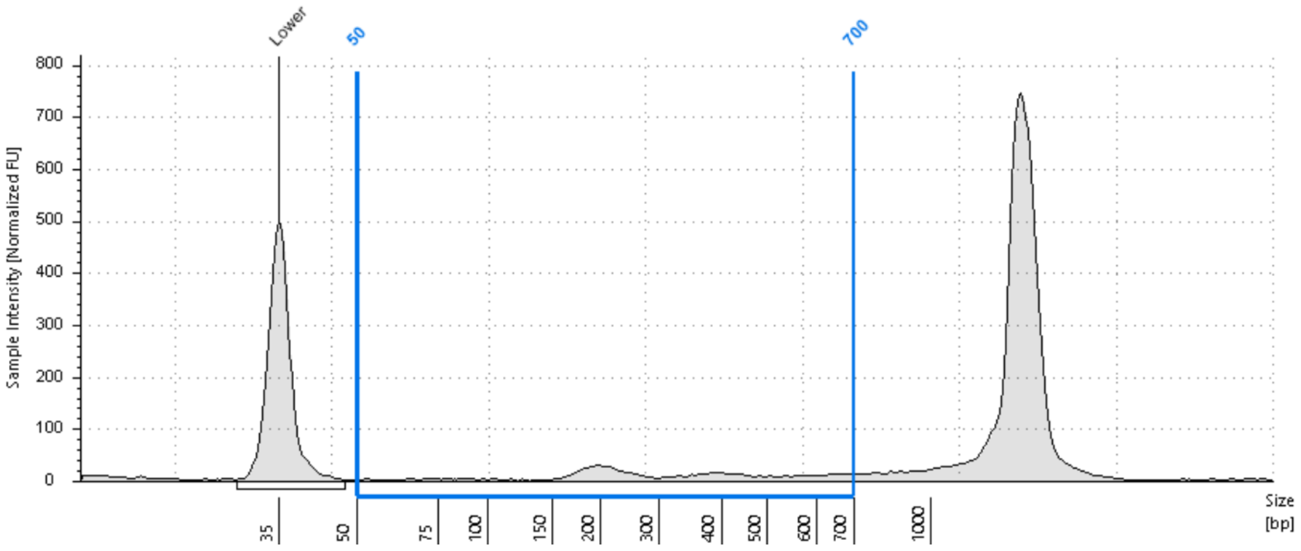

Region Table

From [bp]	To [bp]	Average Size [bp]	Conc. [pg/µl]	Region Molarity [pmol/l]	% of Total	Region Comment	Color
50	700	329	83.0	621	11.88	%cfDNA	■

© Agilent Technologies, Inc. 2019

D1: 3 DNA061695 - D7_plastic_centrifugation

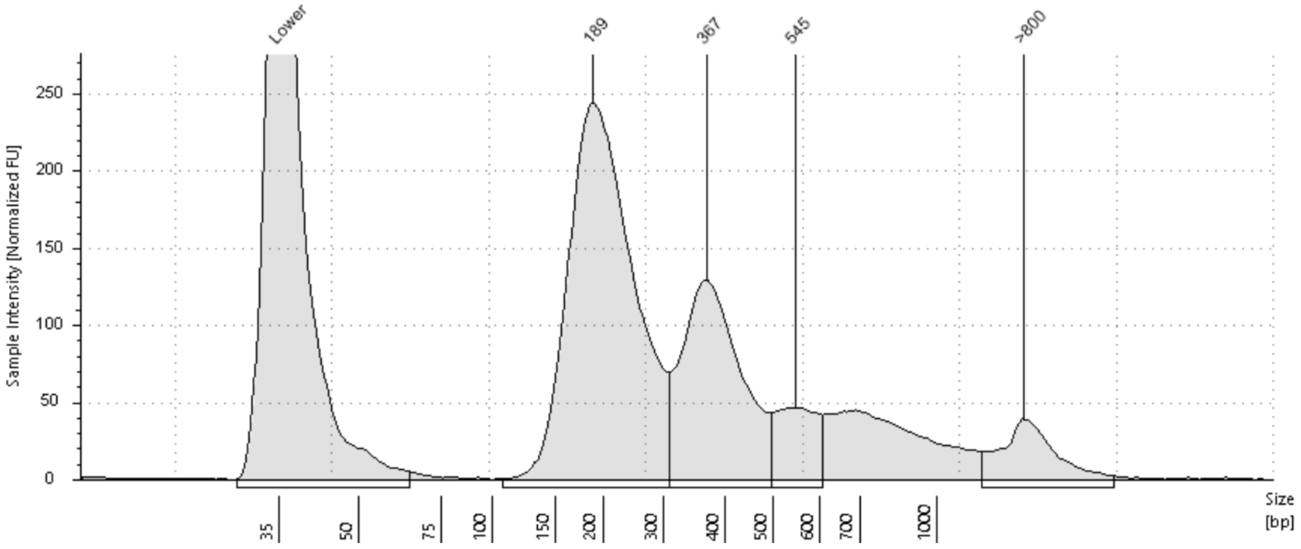

Sample Table

Well	%cfDNA	Conc. [pg/µl]	Sample Description	Alert	Observations
D1	84	509	3		

Peak Table

Size [bp]	Calibrated Conc. [pg/µl]	Assigned Conc. [pg/µl]	Peak Molarity [pmol/l]	% Integrated Area	Height	Peak Comment	Observations
35	275	275	12100	-	311.221		Lower Marker
189	253	-	2060	57.48	152.064		
367	123	-	514	27.92	80.523		
545	32.0	-	90.3	7.29	29.079		
>800	32.1	-	-	7.31	24.363		

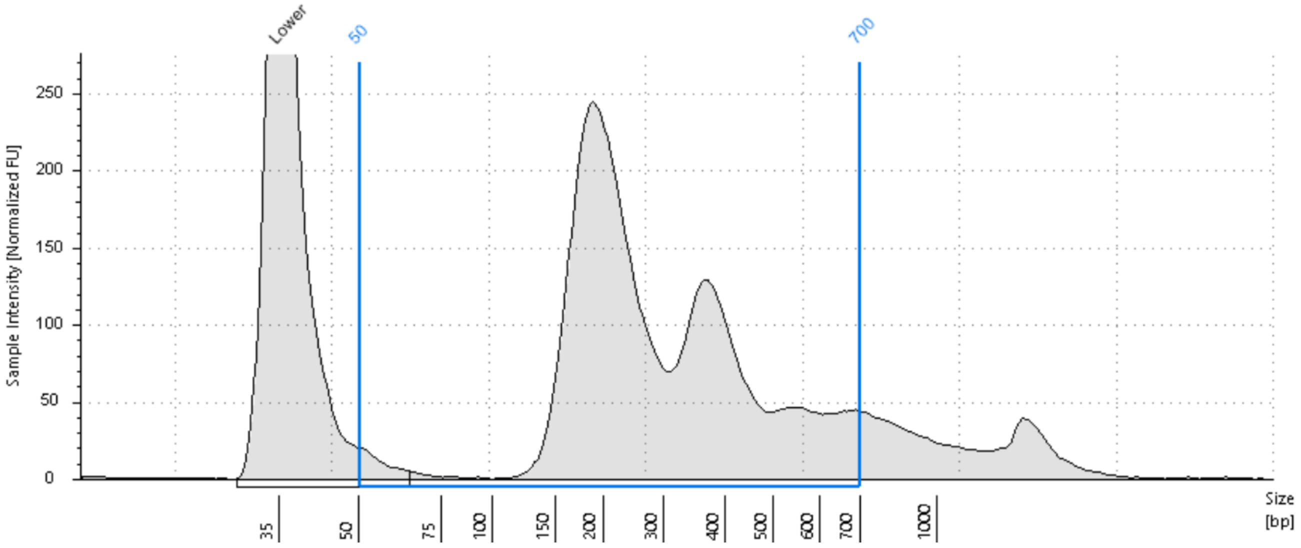

Region Table

From [bp]	To [bp]	Average Size [bp]	Conc. [pg/µl]	Region Molarity [pmol/l]	% of Total	Region Comment	Color
50	700	305	429	2840	84.23	%cfDNA	

© Agilent Technologies, Inc. 2019

E1: 4 DNA061696 - D7_plastic_nocentrifugation

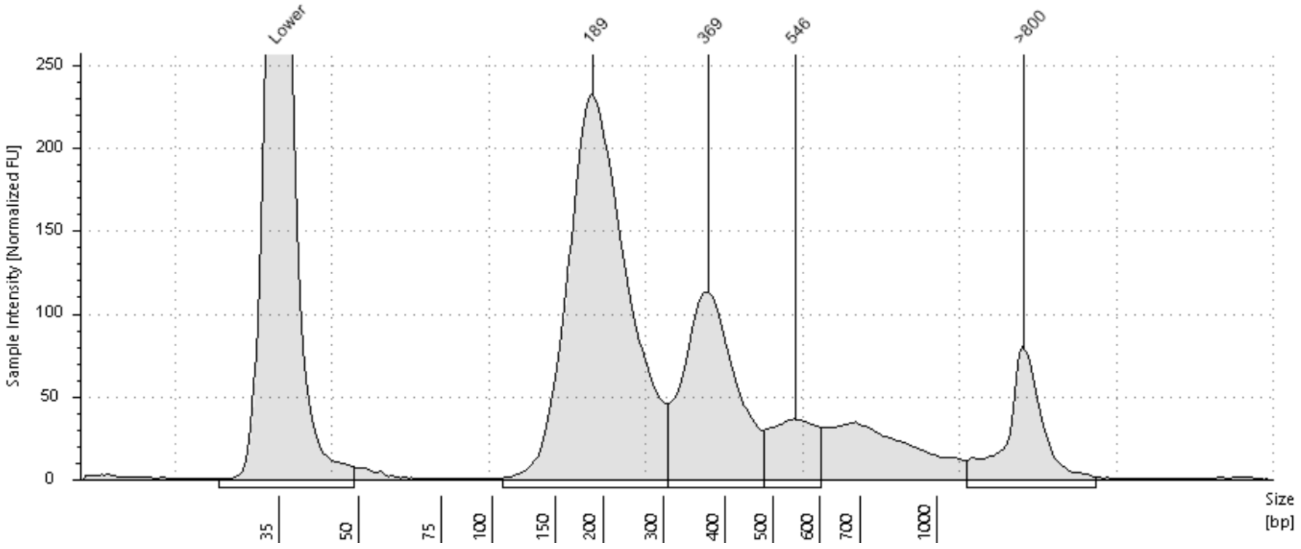

Sample Table

Well	%cfDNA	Conc. [pg/µl]	Sample Description	Alert	Observations
E1	83	551	4		

Peak Table

Size [bp]	Calibrated Conc. [pg/µl]	Assigned Conc. [pg/µl]	Peak Molarity [pmol/l]	% Integrated Area	Height	Peak Comment	Observations
35	275	275	12100	-	308.397		Lower Marker
189	282	-	2300	57.55	143.217		
369	121	-	505	24.69	69.791		
546	34.0	-	95.8	6.94	22.392		
>800	53.1	-	-	10.82	49.269		

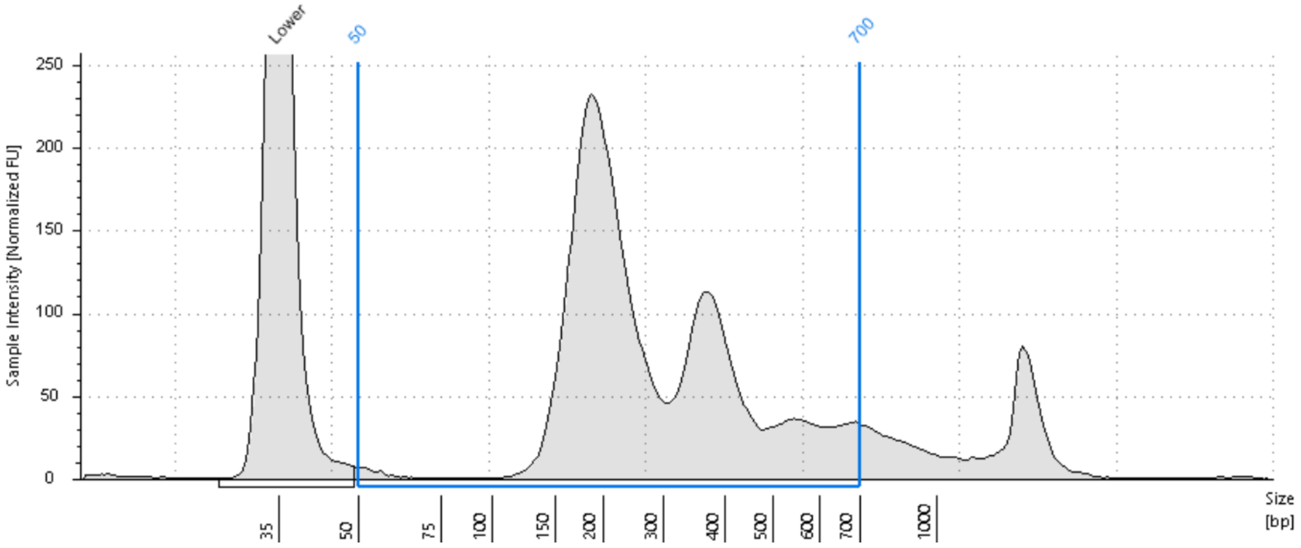

Region Table

From [bp]	To [bp]	Average Size [bp]	Conc. [pg/µl]	Region Molarity [pmol/l]	% of Total	Region Comment	Color
50	700	294	460	2980	83.48	%cfDNA	

 © Agilent Technologies, Inc. 2019

B1: 1 DNA061697 - D8_plastic_centrifugation_supernatant

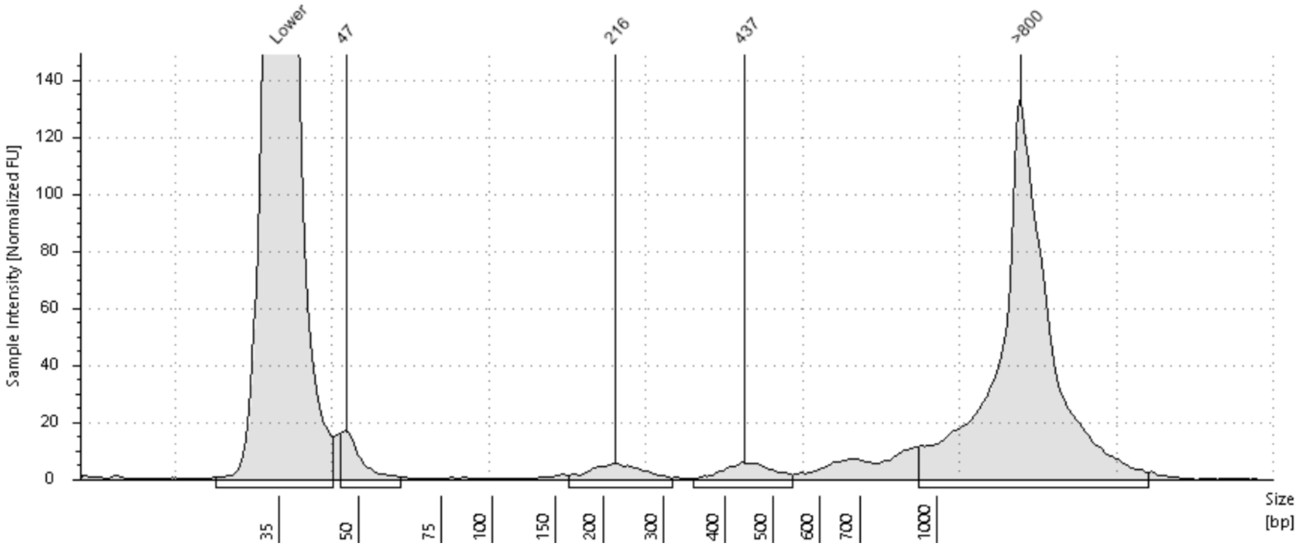

Sample Table

Well	%cfDNA	Conc. [pg/µl]	Sample Description	Alert	Observations
B1	10	161	1		

Peak Table

Size [bp]	Calibrated Conc. [pg/µl]	Assigned Conc. [pg/µl]	Peak Molarity [pmol/l]	% Integrated Area	Height	Peak Comment	Observations
35	275	275	12100	-	471.174		Lower Marker
47	6.54	-	214	4.46	15.904		
216	4.85	-	34.5	3.31	5.313		
437	4.83	-	17.0	3.30	5.472		
>800	130	-	-	88.94	125.228		

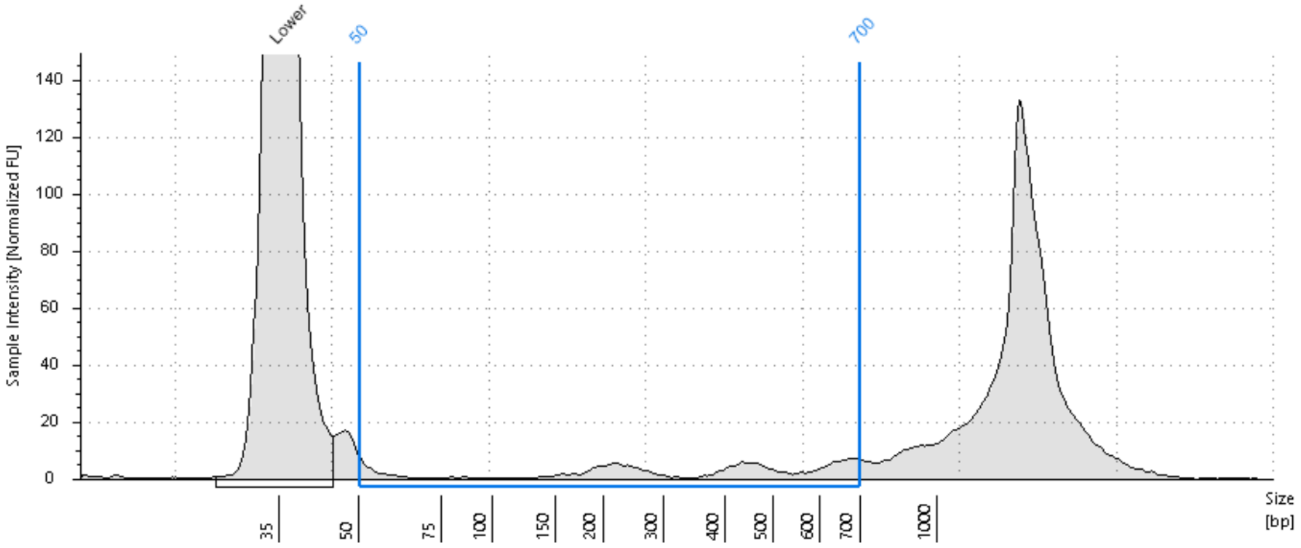

Region Table

From [bp]	To [bp]	Average Size [bp]	Conc. [pg/µl]	Region Molarity [pmol/l]	% of Total	Region Comment	Color
50	700	376	16.3	151	10.13	%cfDNA	

© Agilent Technologies, Inc. 2019

C1: 2 DNA061698 - D8_plastic_nocentrifugation

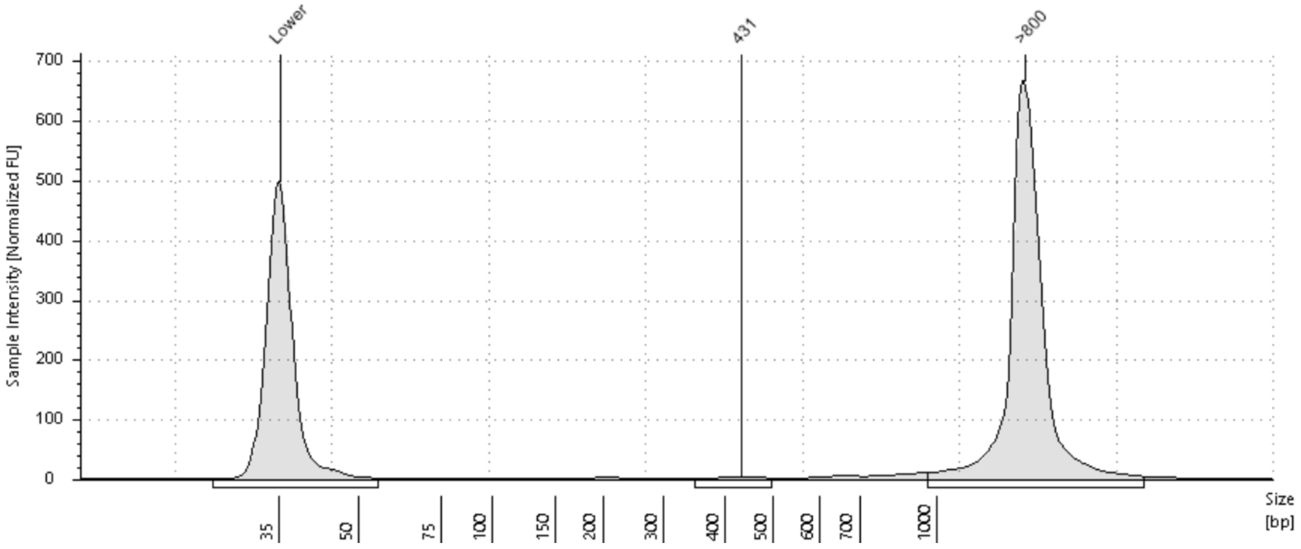

Sample Table

Well	%cfDNA	Conc. [pg/µl]	Sample Description	Alert	Observations
C1	2	455	2		

Peak Table

Size [bp]	Calibrated Conc. [pg/µl]	Assigned Conc. [pg/µl]	Peak Molarity [pmol/l]	% Integrated Area	Height	Peak Comment	Observations
35	275	275	12100	-	452.294		Lower Marker
431	3.10	-	11.1	0.71	3.791		
>800	432	-	-	99.29	604.389		

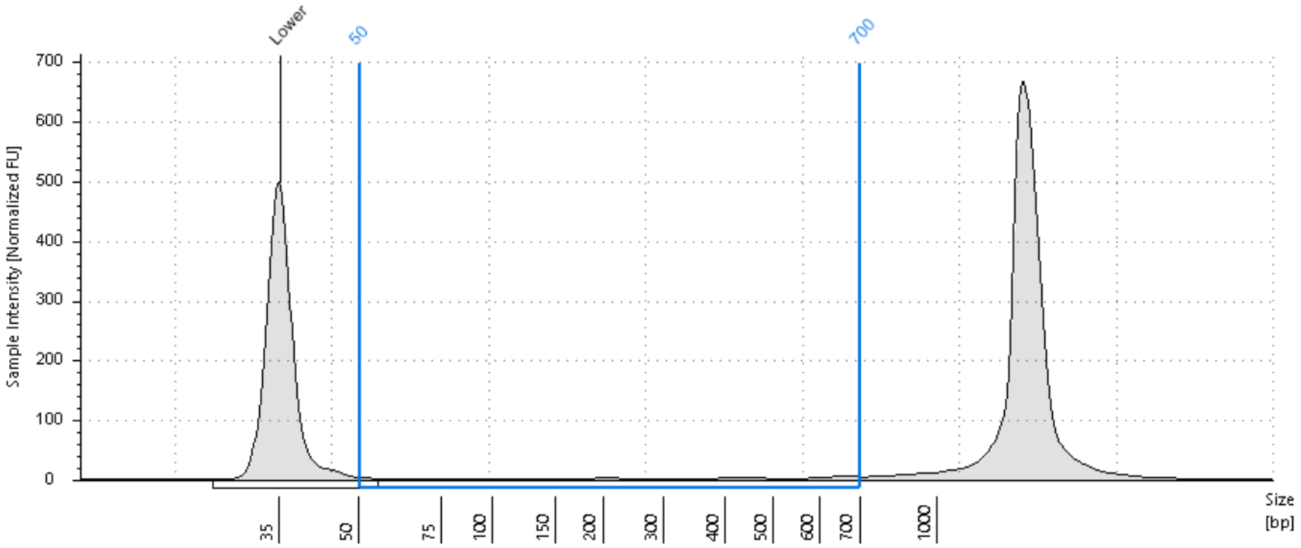

Region Table

From [bp]	To [bp]	Average Size [bp]	Conc. [pg/µl]	Region Molarity [pmol/l]	% of Total	Region Comment	Color
50	700	403	11.1	121	2.44	%cfDNA	

 © Agilent Technologies, Inc. 2019

F1: 5 DNA061699 - D9_plastic_Whole CSF

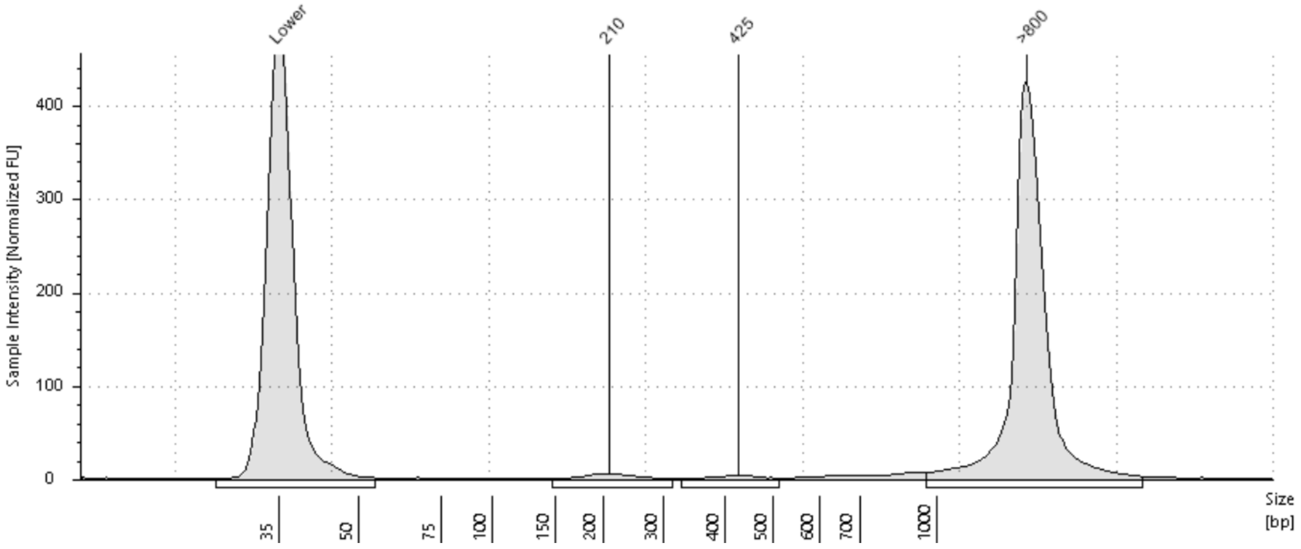

Sample Table

Well	%cfDNA	Conc. [pg/µl]	Sample Description	Alert	Observations
F1	4	293	5		

Peak Table

Size [bp]	Calibrated Conc. [pg/µl]	Assigned Conc. [pg/µl]	Peak Molarity [pmol/l]	% Integrated Area	Height	Peak Comment	Observations
35	275	275	12100	-	489.842		Lower Marker
210	5.32	-	39.1	1.88	6.178		
425	2.88	-	10.4	1.02	3.985		
>800	274	-	-	97.10	416.909		

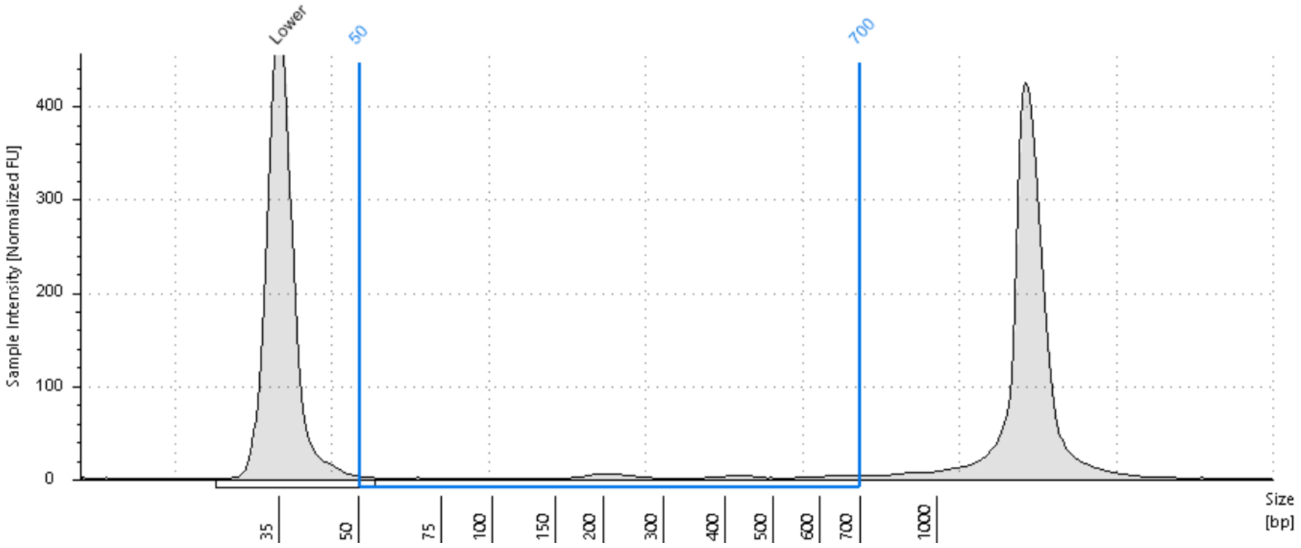

Region Table

From [bp]	To [bp]	Average Size [bp]	Conc. [pg/µl]	Region Molarity [pmol/l]	% of Total	Region Comment	Color
50	700	348	11.7	132	4.00	%cfDNA	

© Agilent Technologies, Inc. 2019

G1: 6 DNA061700 - D9_plastic_centrifugation_supernatant

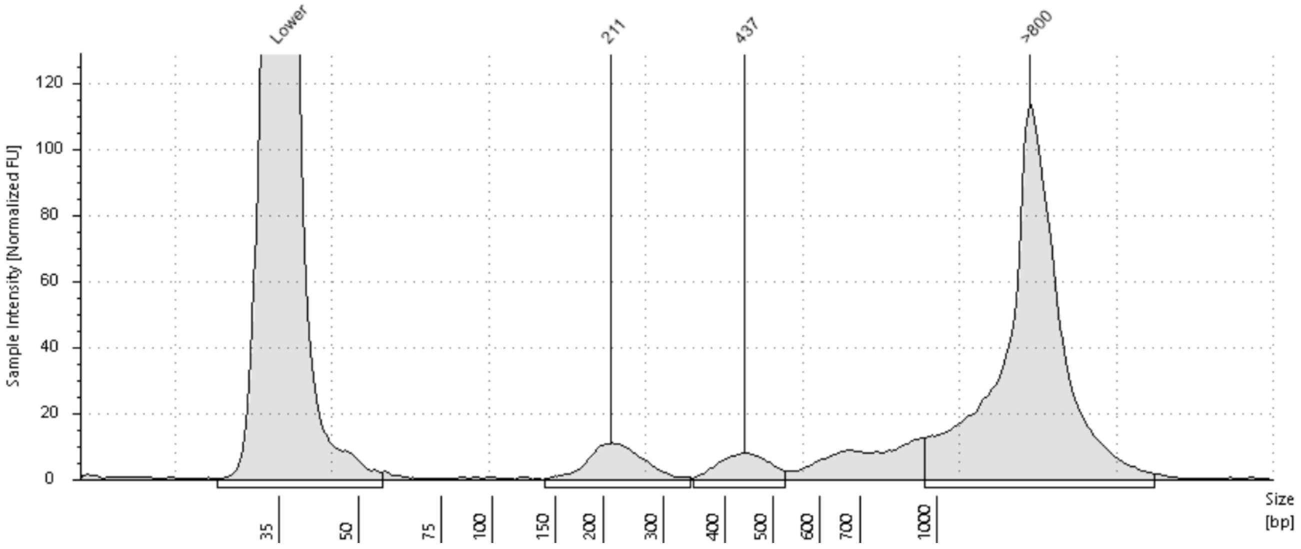

Sample Table

Well	%cfDNA	Conc. [pg/µl]	Sample Description	Alert	Observations
G1	14	151	6		

Peak Table

Size [bp]	Calibrated Conc. [pg/µl]	Assigned Conc. [pg/µl]	Peak Molarity [pmol/l]	% Integrated Area	Height	Peak Comment	Observations
35	275	275	12100	-	487.133		Lower Marker
211	9.31	-	67.8	6.85	10.729		
437	6.04	-	21.3	4.45	7.789		
>800	120	-	-	88.70	110.921		

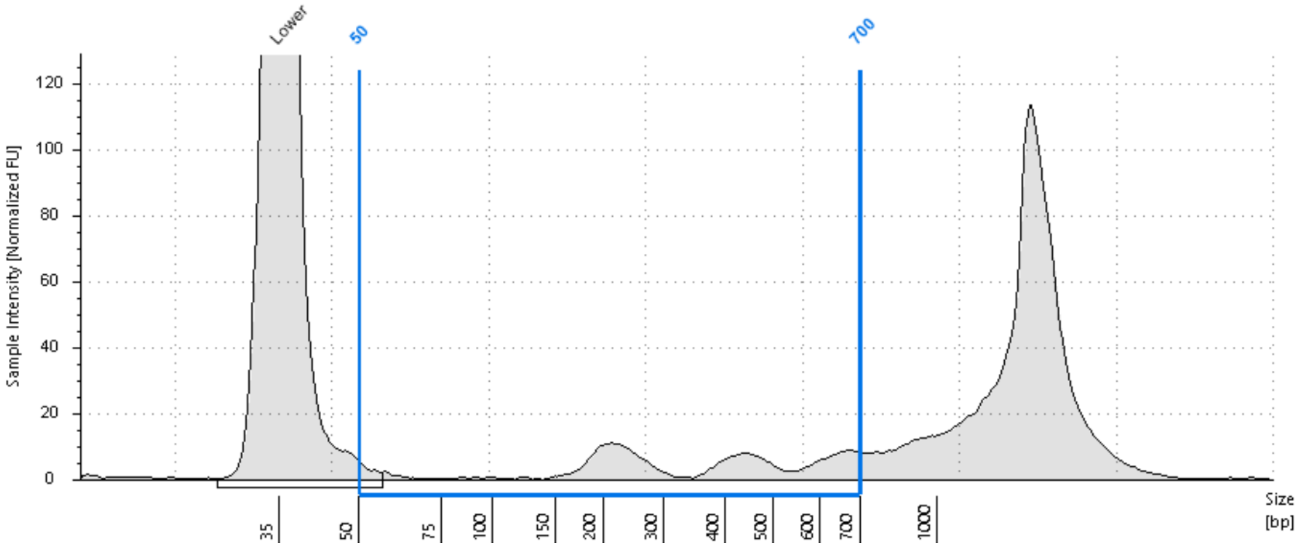

Region Table

From [bp]	To [bp]	Average Size [bp]	Conc. [pg/µl]	Region Molarity [pmol/l]	% of Total	Region Comment	Color
50	700	381	21.3	207	14.15	%cfDNA	

© Agilent Technologies, Inc. 2019

B1: DNA080827 - D10_CSF_Centrifuged

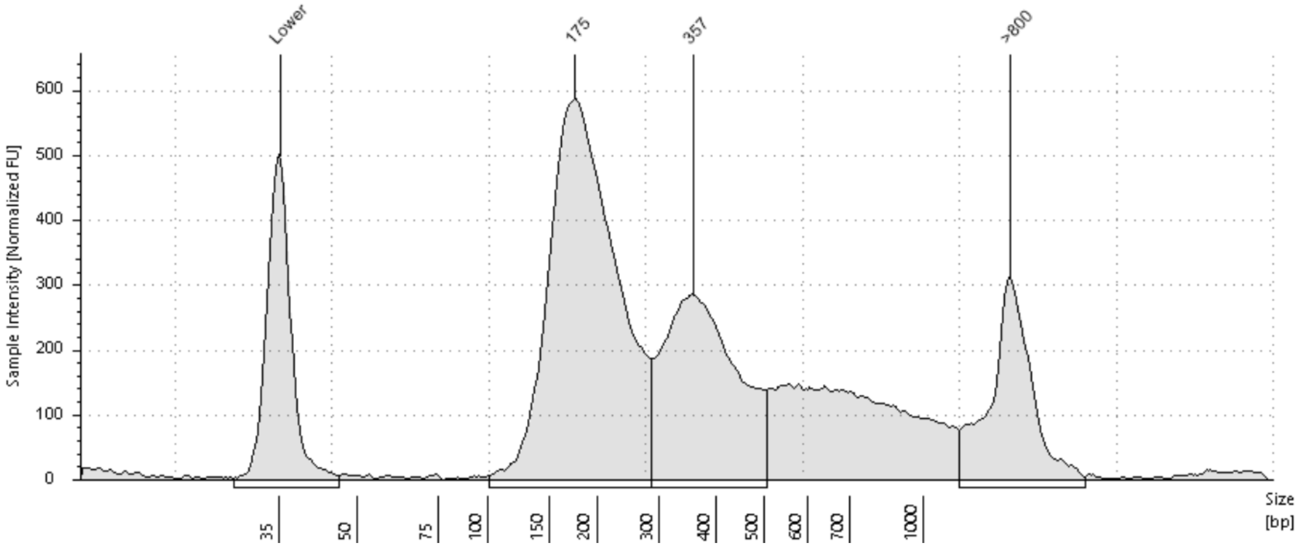

Sample Table

Well	%cfDNA	Conc. [pg/µl]	Sample Description	Alert	Observations
B1	76	2150	CSF78 (Cent)		

Peak Table

Size [bp]	Calibrated Conc. [pg/µl]	Assigned Conc. [pg/µl]	Peak Molarity [pmol/l]	% Integrated Area	Height	Peak Comment	Observations
35	275	275	12100	-	53.599		Lower Marker
175	906	-	7950	53.98	62.923		
357	486	-	2100	28.97	30.628		
>800	286	-	-	17.05	33.329		

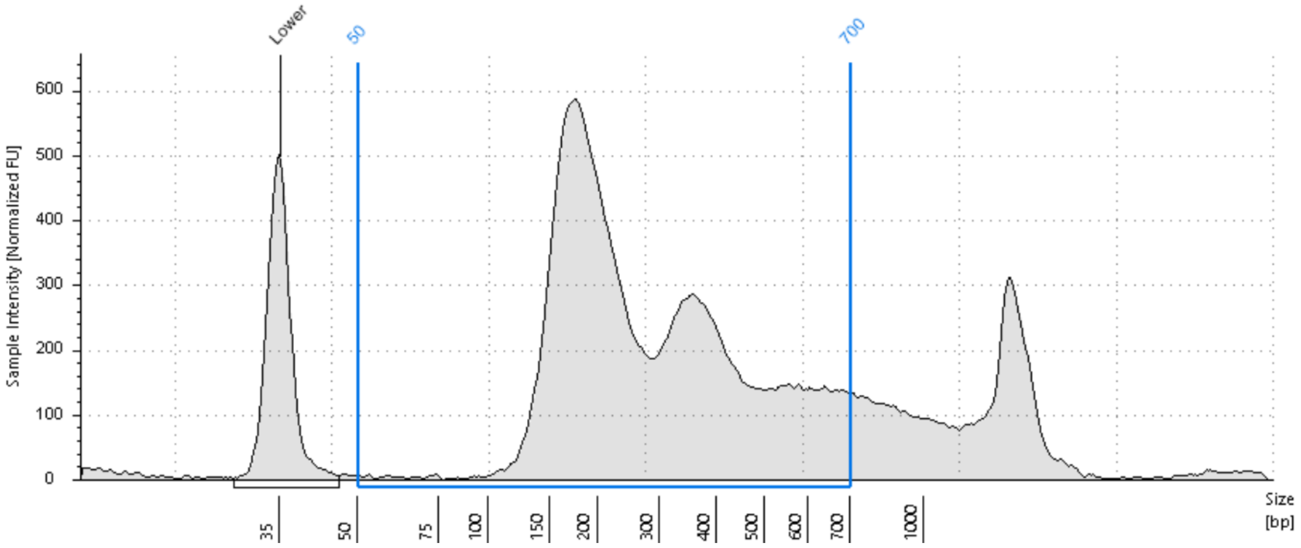

Region Table

From [bp]	To [bp]	Average Size [bp]	Conc. [pg/µl]	Region Molarity [pmol/l]	% of Total	Region Comment	Color
50	700	306	1620	10400	75.59	%cfDNA	

© Agilent Technologies, Inc. 2021

C1: DNA080826 - D10_CSF_Whole

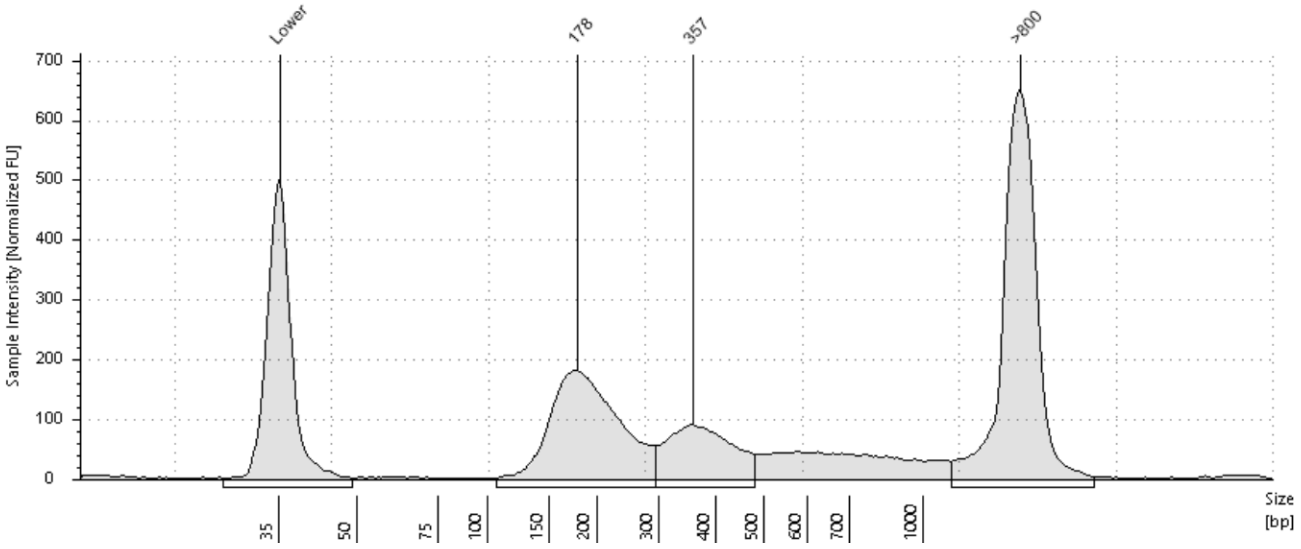

Sample Table

Well	%cfDNA	Conc. [pg/µl]	Sample Description	Alert	Observations
C1	47	1070	CSF78 (Whole)		

Peak Table

Size [bp]	Calibrated Conc. [pg/µl]	Assigned Conc. [pg/µl]	Peak Molarity [pmol/l]	% Integrated Area	Height	Peak Comment	Observations
35	275	275	12100	-	95.157		Lower Marker
178	280	-	2420	30.90	34.718		
357	136	-	584	14.94	17.399		
>800	491	-	-	54.15	123.588		

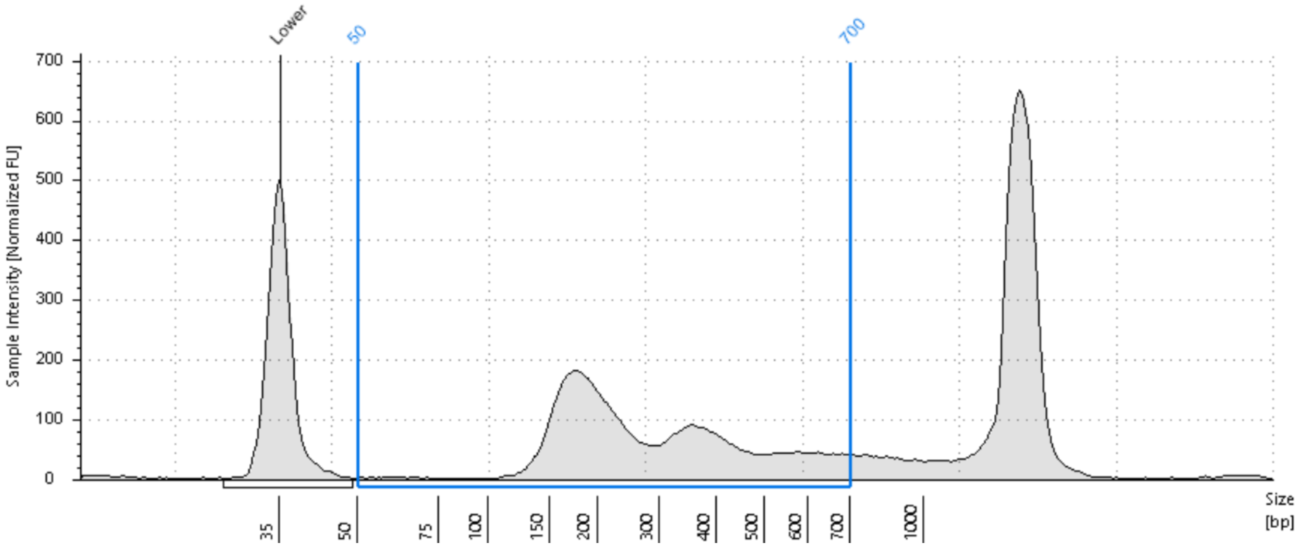

Region Table

From [bp]	To [bp]	Average Size [bp]	Conc. [pg/µl]	Region Molarity [pmol/l]	% of Total	Region Comment	Color
50	700	305	499	3240	46.82	%cfDNA	

© Agilent Technologies, Inc. 2021

D1:DNA080829 - D11_CSF_Centrifuged

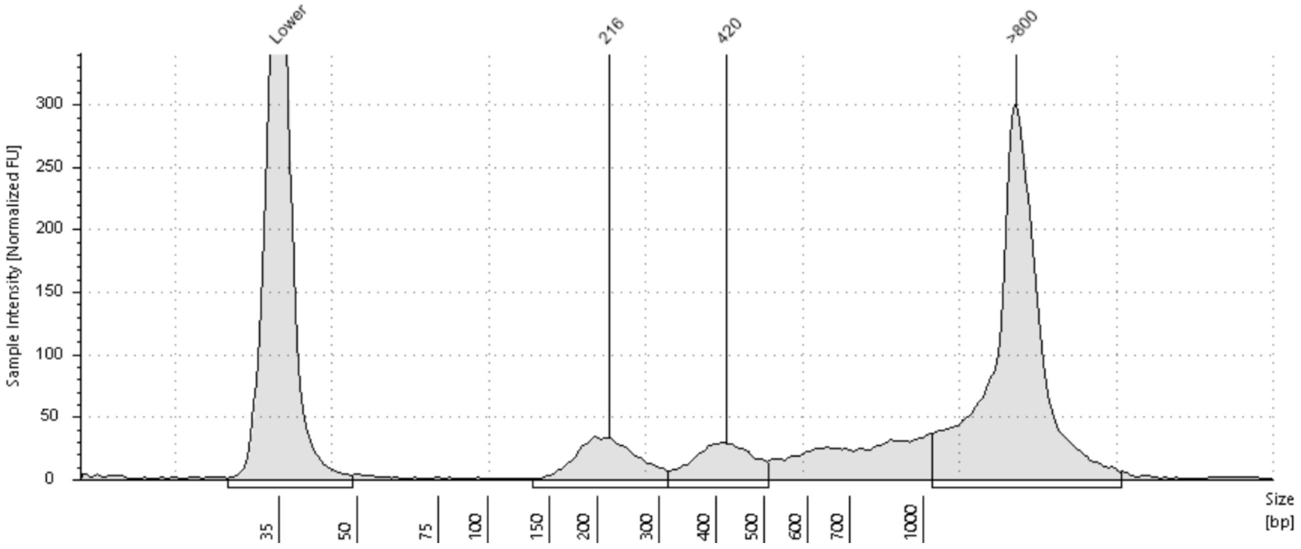

Sample Table

Well	%cfDNA	Conc. [pg/µl]	Sample Description	Alert	Observations
D1	26	463	CSF79 (Cent)		

Peak Table

Size [bp]	Calibrated Conc. [pg/µl]	Assigned Conc. [pg/µl]	Peak Molarity [pmol/l]	% Integrated Area	Height	Peak Comment	Observations
35	275	275	12100	-	147.778		Lower Marker
216	45.2	-	321	11.94	9.767		
420	38.1	-	140	10.07	8.652		
>800	295	-	-	77.99	88.558		

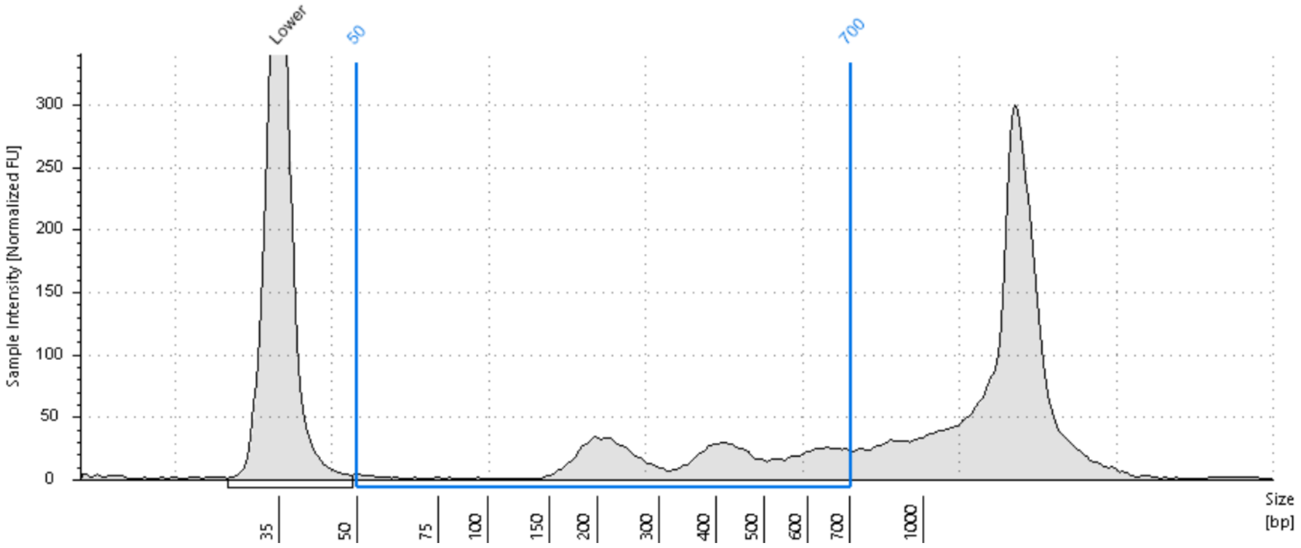

Region Table

From [bp]	To [bp]	Average Size [bp]	Conc. [pg/µl]	Region Molarity [pmol/l]	% of Total	Region Comment	Color
50	700	386	119	647	25.64	%cfDNA	

© Agilent Technologies, Inc. 2021

E1: DNA080828 - D11_CSF_whole

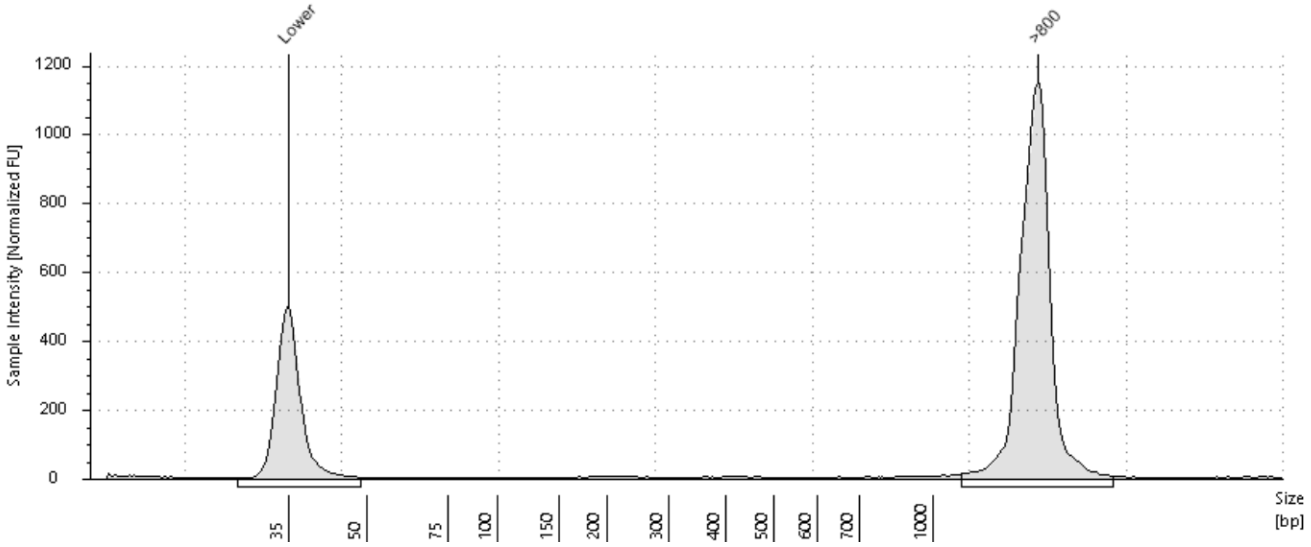

Sample Table

Well	%cfDNA	Conc. [pg/µl]	Sample Description	Alert	Observations
E1	3	808	CSF79 (Whole)		

Peak Table

Size [bp]	Calibrated Conc. [pg/µl]	Assigned Conc. [pg/µl]	Peak Molarity [pmol/l]	% Integrated Area	Height	Peak Comment	Observations
35	275	275	12100	-	100.088		Lower Marker
>800	768	-	-	100.00	231.113		

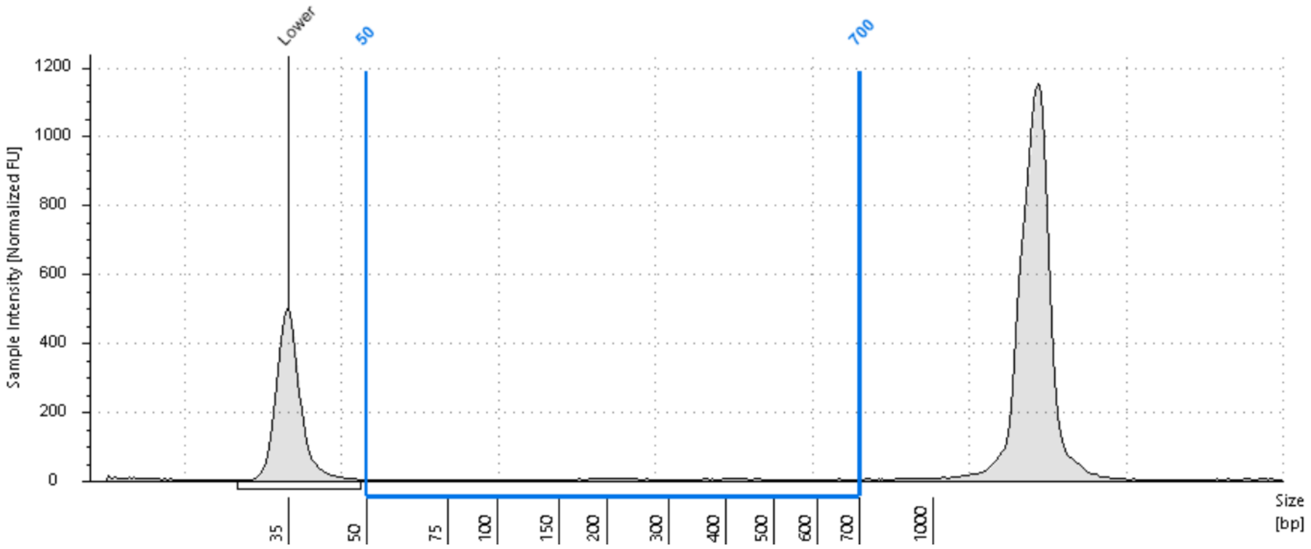

Region Table

From [bp]	To [bp]	Average Size [bp]	Conc. [pg/µl]	Region Molarity [pmol/l]	% of Total	Region Comment	Color
50	700	311	20.7	209	2.56	%cfDNA	

D1: DNA086728 - D13_CSF_Centrifuged

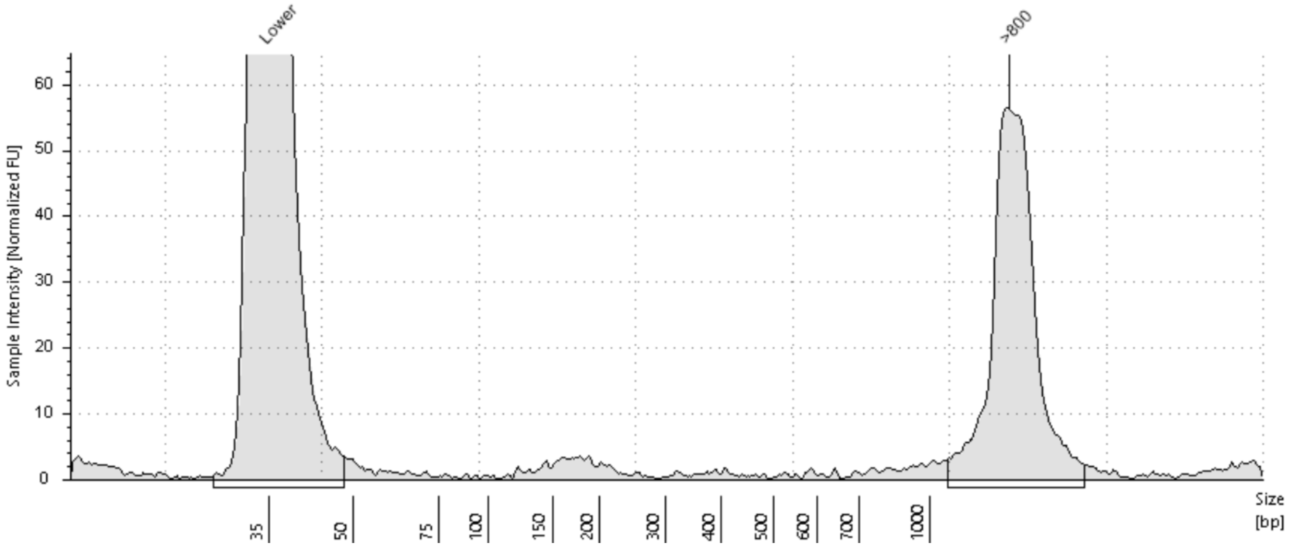

Sample Table

Well	%cfDNA	Conc. [pg/µl]	Sample Description	Alert	Observations
D1	12	67.7	D13_CSF_Centr	⚠	Sample concentration outside functional range for %cfDNA and the assay

Peak Table

Size [bp]	Calibrated Conc. [pg/µl]	Assigned Conc. [pg/µl]	Peak Molarity [pmol/l]	% Integrated Area	Height	Peak Comment	Observations
35	275	275	12100	-	253.573		Lower Marker
>800	53.3	-	-	100.00	28.549		

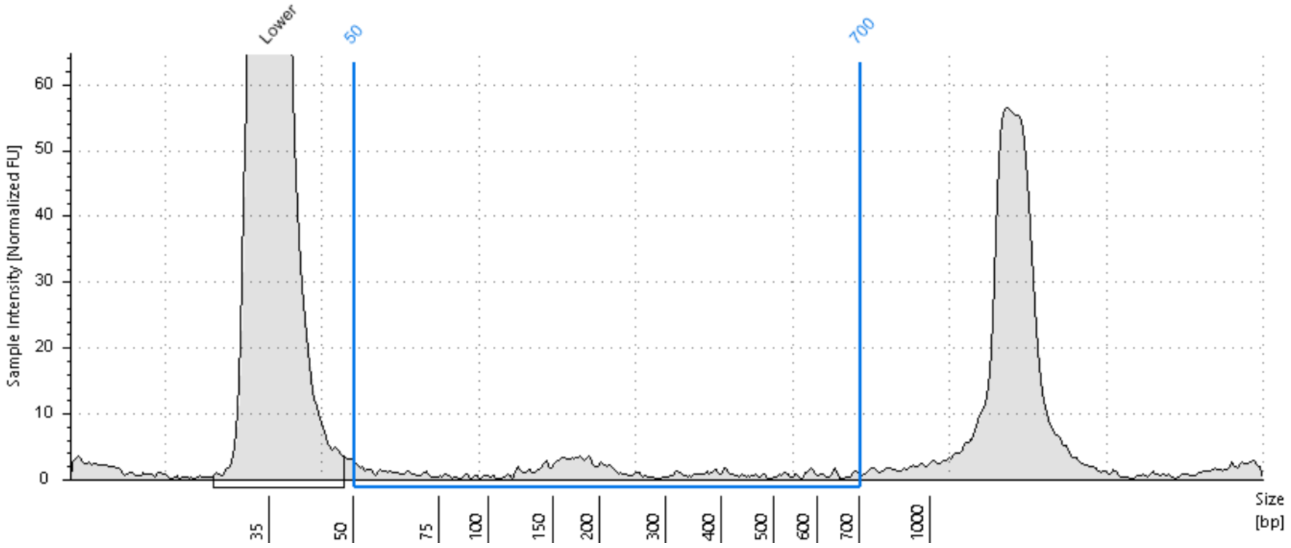

Region Table

From [bp]	To [bp]	Average Size [bp]	Conc. [pg/µl]	Region Molarity [pmol/l]	% of Total	Region Comment	Color
50	700	236	7.89	112	11.66	%cfDNA	■

© Agilent Technologies, Inc. 2021

E1: DNA086728 - D13_CSF_Whole

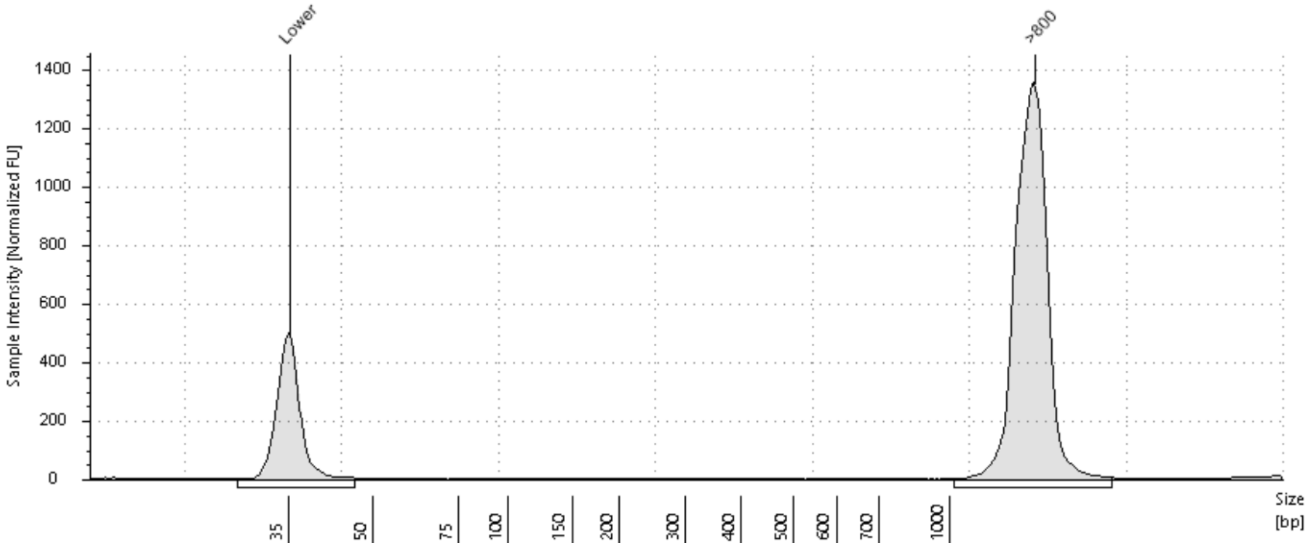

Sample Table

Well	%cfDNA	Conc. [pg/µl]	Sample Description	Alert	Observations
E1	1	1050	D13_CF_whole		

Peak Table

Size [bp]	Calibrated Conc. [pg/µl]	Assigned Conc. [pg/µl]	Peak Molarity [pmol/l]	% Integrated Area	Height	Peak Comment	Observations
35	275	275	12100	-	121.152		Lower Marker
>800	1030	-	-	100.00	329.375		

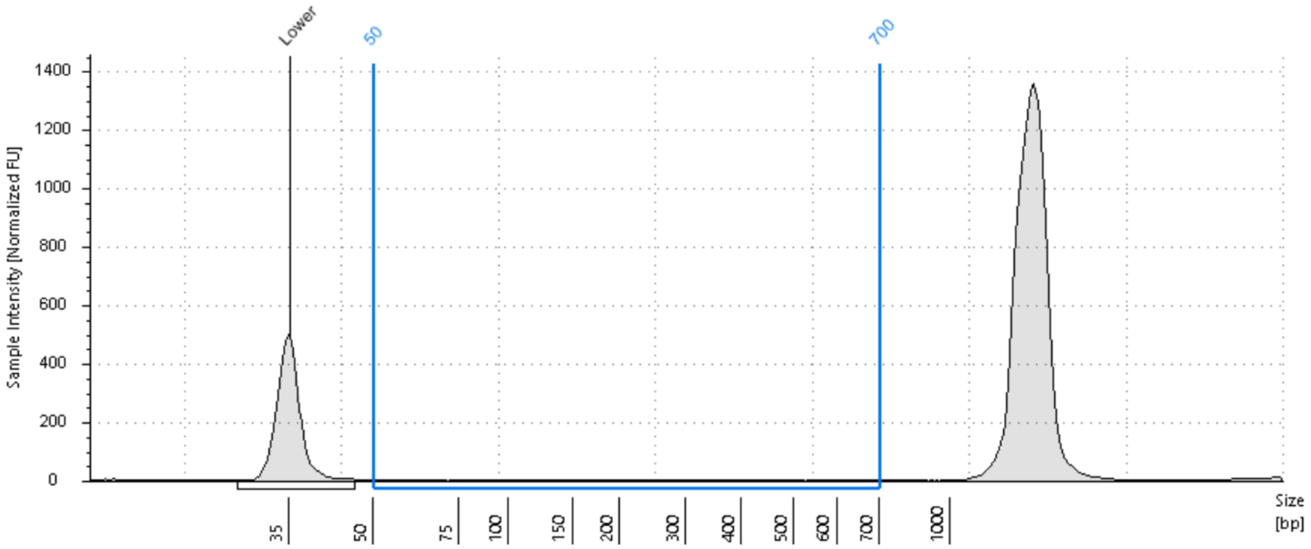

Region Table

From [bp]	To [bp]	Average Size [bp]	Conc. [pg/µl]	Region Molarity [pmol/l]	% of Total	Region Comment	Color
50	700	257	12.8	172	1.22	%cfDNA	

© Agilent Technologies, Inc. 2021

C1: DNA086728 - 13_plastic_Centrifuged_CSF

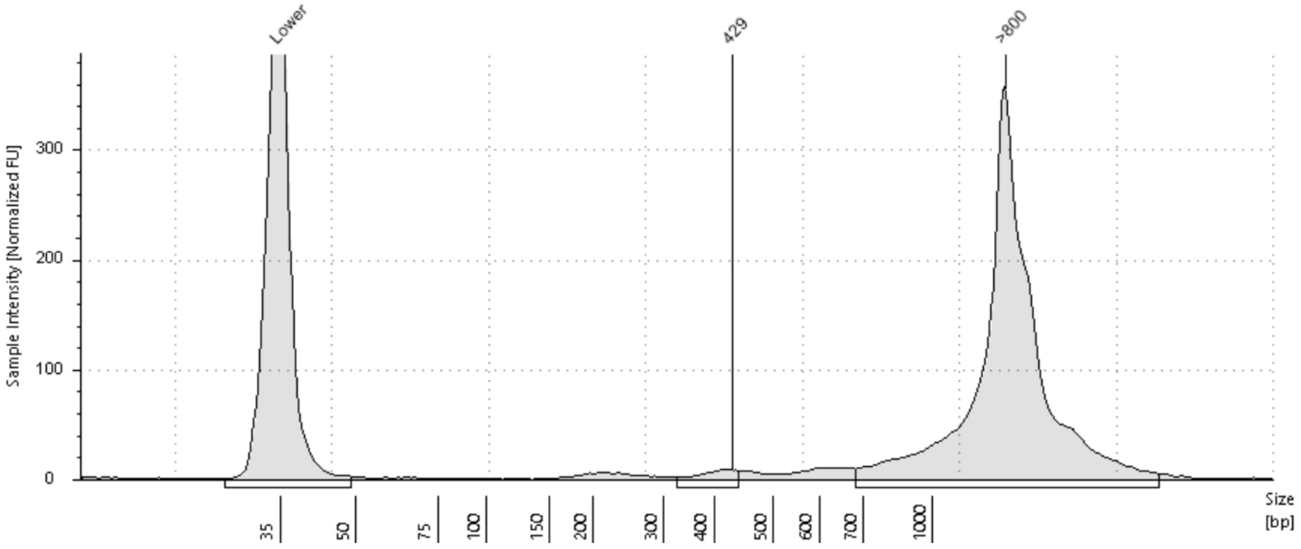

Sample Table

Well	%cfDNA	Conc. [pg/µl]	Sample Description	Alert	Observations
C1	8	452	DNA086728CSF		

Peak Table

Size [bp]	Calibrated Conc. [pg/µl]	Assigned Conc. [pg/µl]	Peak Molarity [pmol/l]	% Integrated Area	Height	Peak Comment	Observations
35	275	275	12100	-	250.188		Lower Marker
429	6.88	-	24.7	1.64	4.433		
>800	414	-	-	98.36	178.549		

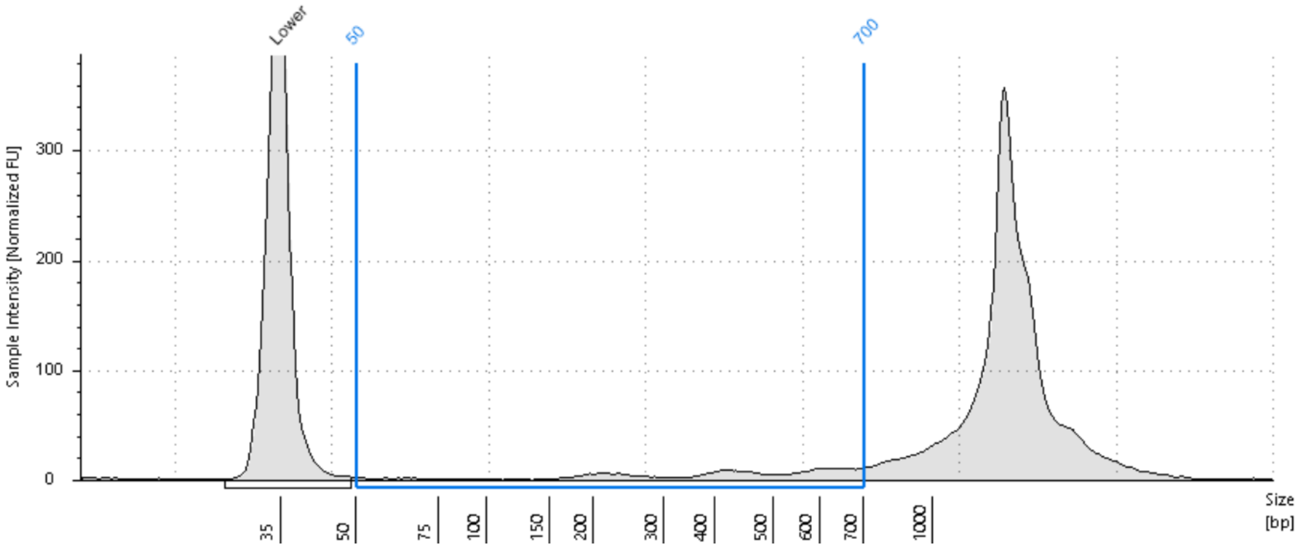

Region Table

From [bp]	To [bp]	Average Size [bp]	Conc. [pg/µl]	Region Molarity [pmol/l]	% of Total	Region Comment	Color
50	700	430	37.9	215	8.38	%cfDNA	

© Agilent Technologies, Inc. 2021

D1: DNA086729 - 14_plastic_Centrifuged_CSF

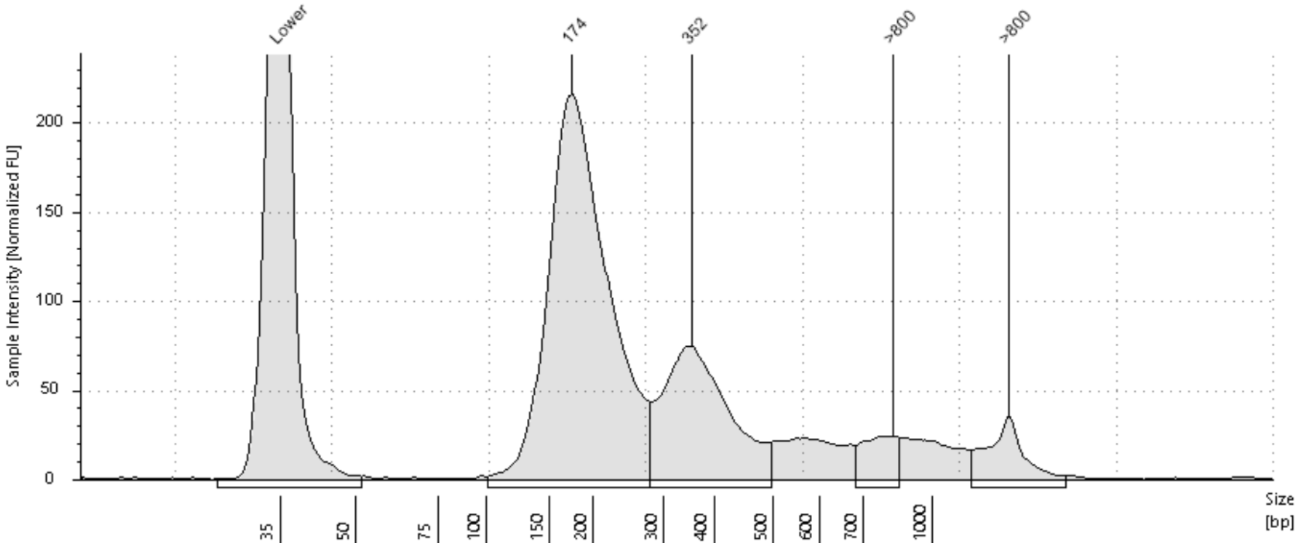

Sample Table

Well	%cfDNA	Conc. [pg/µl]	Sample Description	Alert	Observations
D1	86	556	DNA086729CSF		

Peak Table

Size [bp]	Calibrated Conc. [pg/µl]	Assigned Conc. [pg/µl]	Peak Molarity [pmol/l]	% Integrated Area	Height	Peak Comment	Observations
35	275	275	12100	-	301.706		Lower Marker
174	314	-	2780	64.51	130.548		
352	122	-	531	25.01	45.189		
>800	21.3	-	-	4.38	14.538		
>800	29.6	-	-	6.10	21.495		

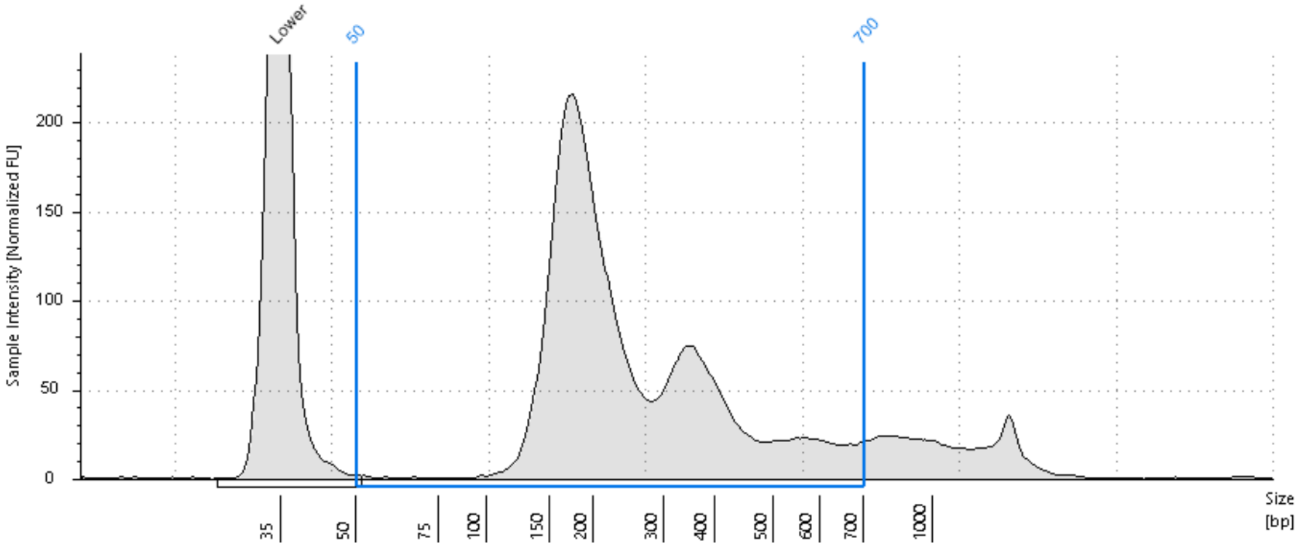

Region Table

From [bp]	To [bp]	Average Size [bp]	Conc. [pg/µl]	Region Molarity [pmol/l]	% of Total	Region Comment	Color
50	700	269	477	3320	85.83	%cfDNA	

 © Agilent Technologies, Inc. 2021

F1: DNA086731 - 15_plastic_Centrifuged_CSF

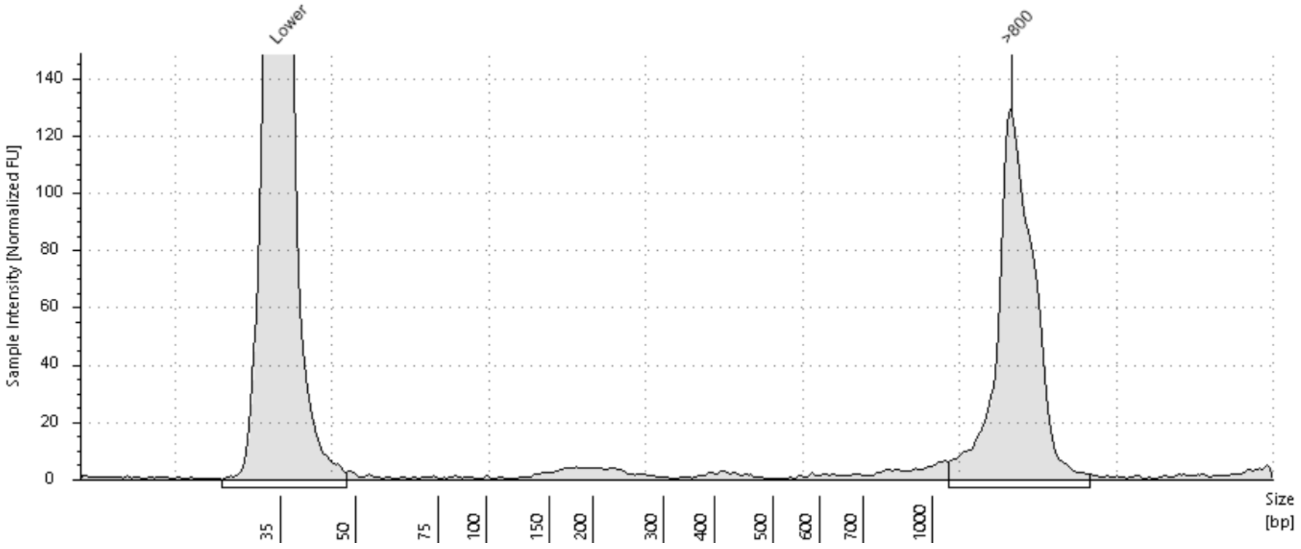

Sample Table

Well	%cfDNA	Conc. [pg/µl]	Sample Description	Alert	Observations
F1	10	142	DNA086731CSF		

Peak Table

Size [bp]	Calibrated Conc. [pg/µl]	Assigned Conc. [pg/µl]	Peak Molarity [pmol/l]	% Integrated Area	Height	Peak Comment	Observations
35	275	275	12100	-	257.567		Lower Marker
>800	116	-	-	100.00	66.436		

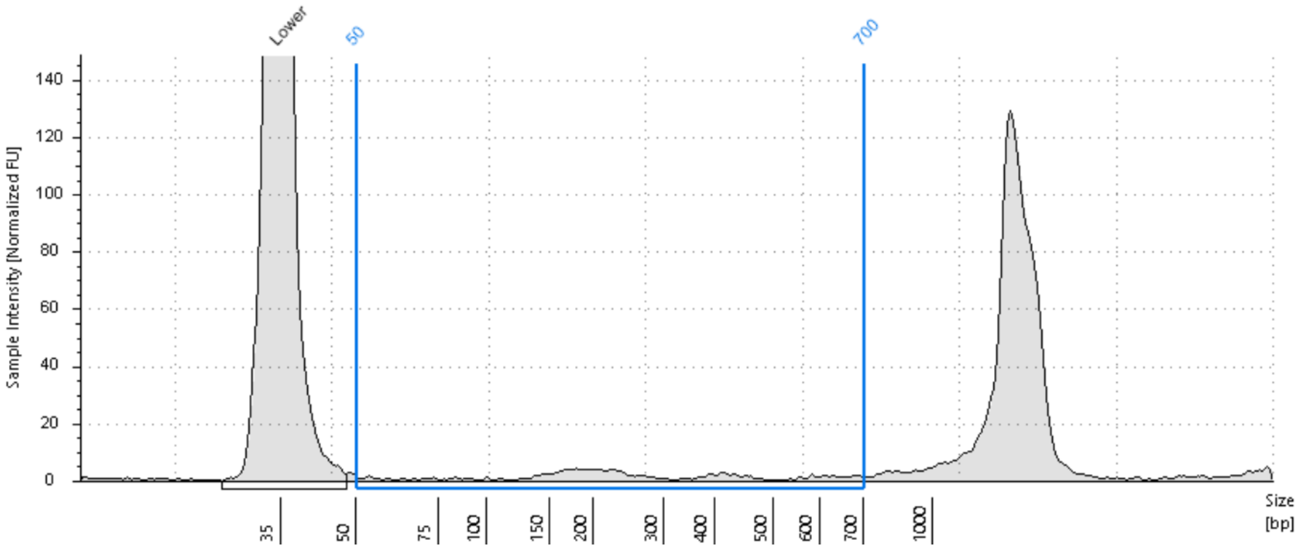

Region Table

From [bp]	To [bp]	Average Size [bp]	Conc. [pg/µl]	Region Molarity [pmol/l]	% of Total	Region Comment	Color
50	700	283	14.0	132	9.86	%cfDNA	

© Agilent Technologies, Inc. 2021

A1: DNA089977 - 16_plastic_centrifuged

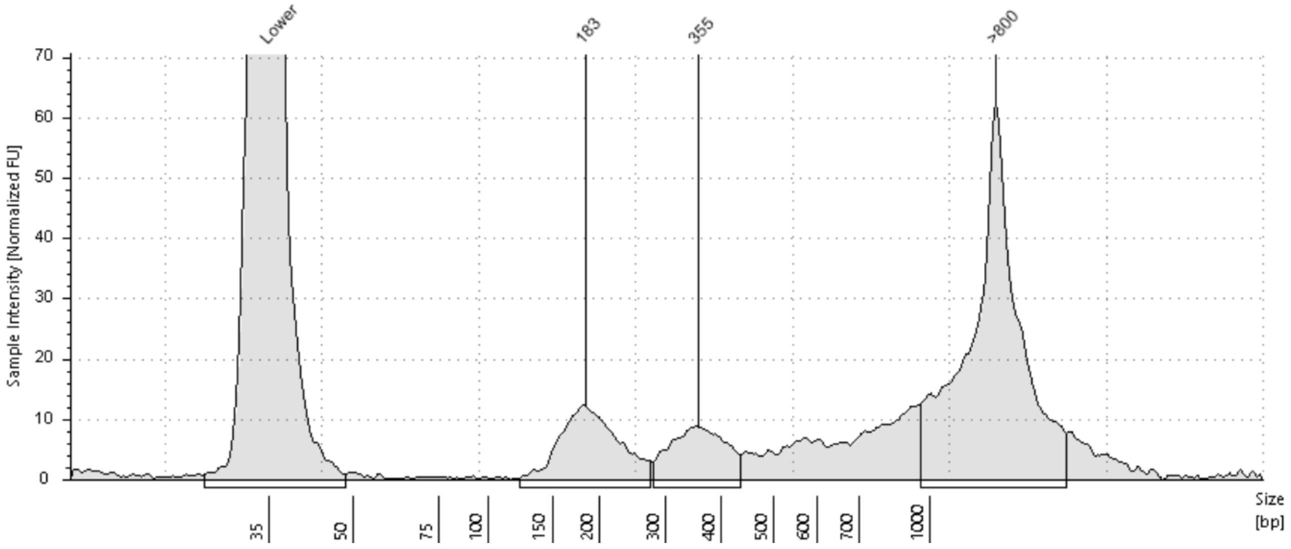

Sample Table

Well	%cfDNA	Conc. [pg/µl]	Sample Description	Alert	Observations
A1	32	144	DNA089977		

Peak Table

Size [bp]	Calibrated Conc. [pg/µl]	Assigned Conc. [pg/µl]	Peak Molarity [pmol/l]	% Integrated Area	Height	Peak Comment	Observations
35	275	275	12100	-	310.140		Lower Marker
183	18.0	-	152	16.74	7.671		
355	12.8	-	55.5	11.88	5.411		
>800	76.9	-	-	71.38	38.657		

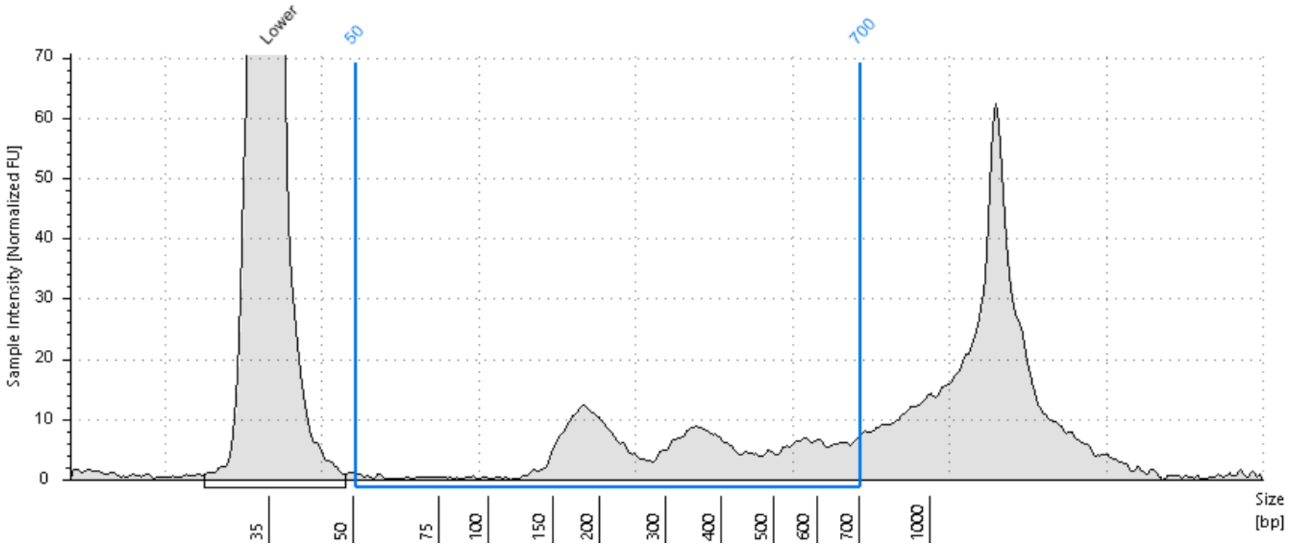

Region Table

From [bp]	To [bp]	Average Size [bp]	Conc. [pg/µl]	Region Molarity [pmol/l]	% of Total	Region Comment	Color
50	700	353	45.9	283	31.82	%cfDNA	

B1: DNA089978 - 17_plastic_centrifuged

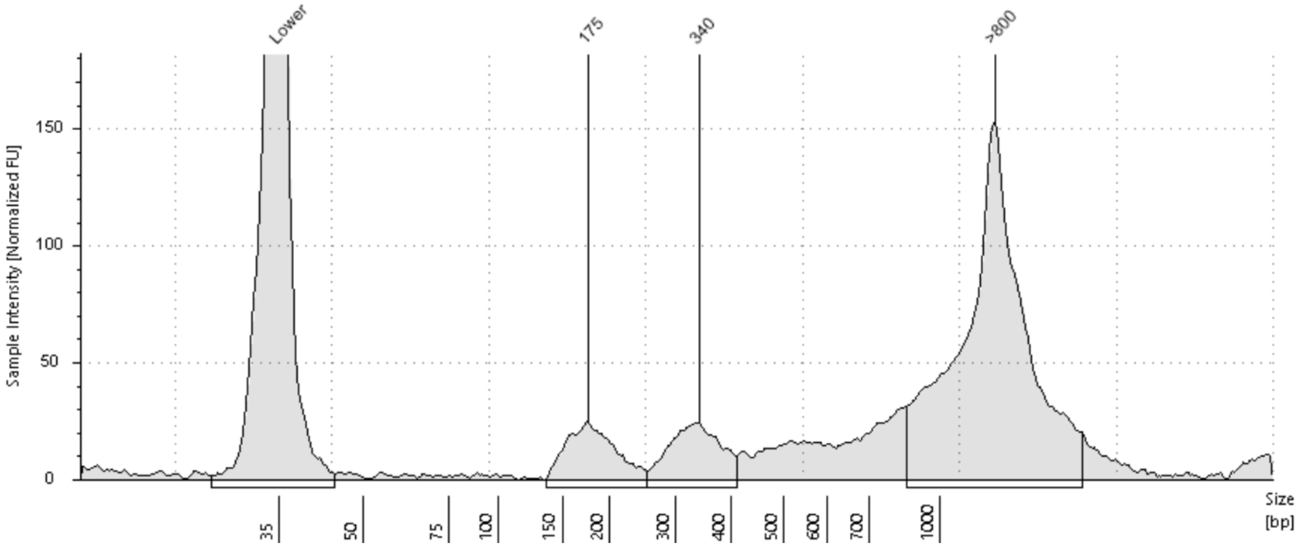

Sample Table

Well	%cfDNA	Conc. [pg/µl]	Sample Description	Alert	Observations
B1	27	390	DNA089978		

Peak Table

Size [bp]	Calibrated Conc. [pg/µl]	Assigned Conc. [pg/µl]	Peak Molarity [pmol/l]	% Integrated Area	Height	Peak Comment	Observations
35	275	275	12100	-	102.292		Lower Marker
175	29.9	-	263	9.93	5.037		
340	31.7	-	143	10.50	5.027		
>800	240	-	-	79.57	31.298		

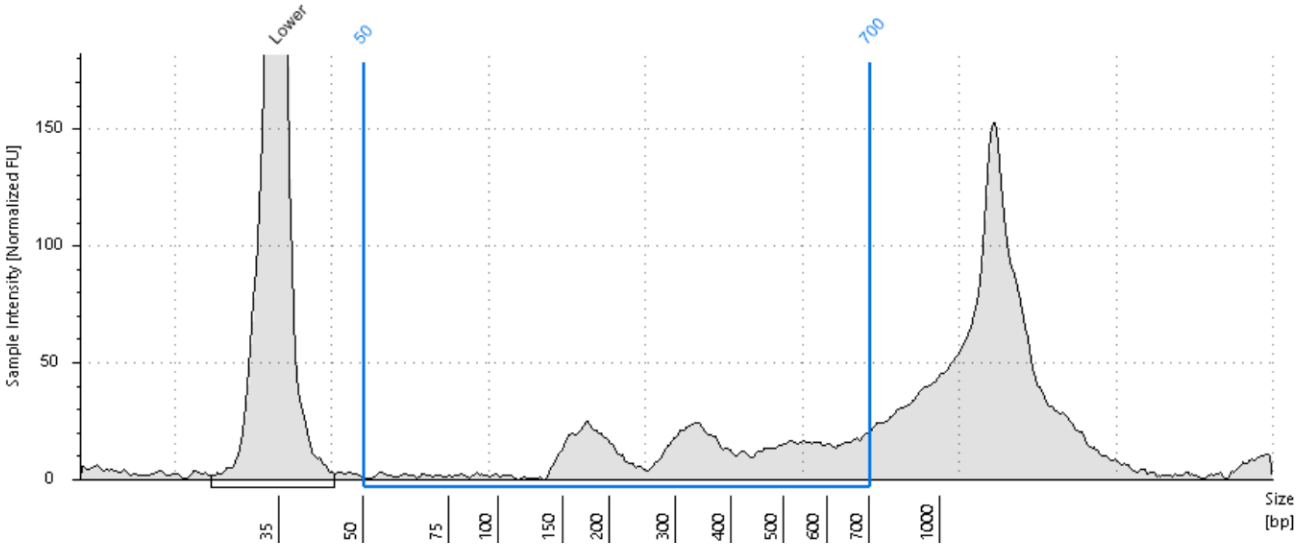

Region Table

From [bp]	To [bp]	Average Size [bp]	Conc. [pg/µl]	Region Molarity [pmol/l]	% of Total	Region Comment	Color
50	700	368	107	673	27.46	%cfDNA	

© Agilent Technologies, Inc. 2021

B2: DNA102082 - D18_plastic_centrifuged

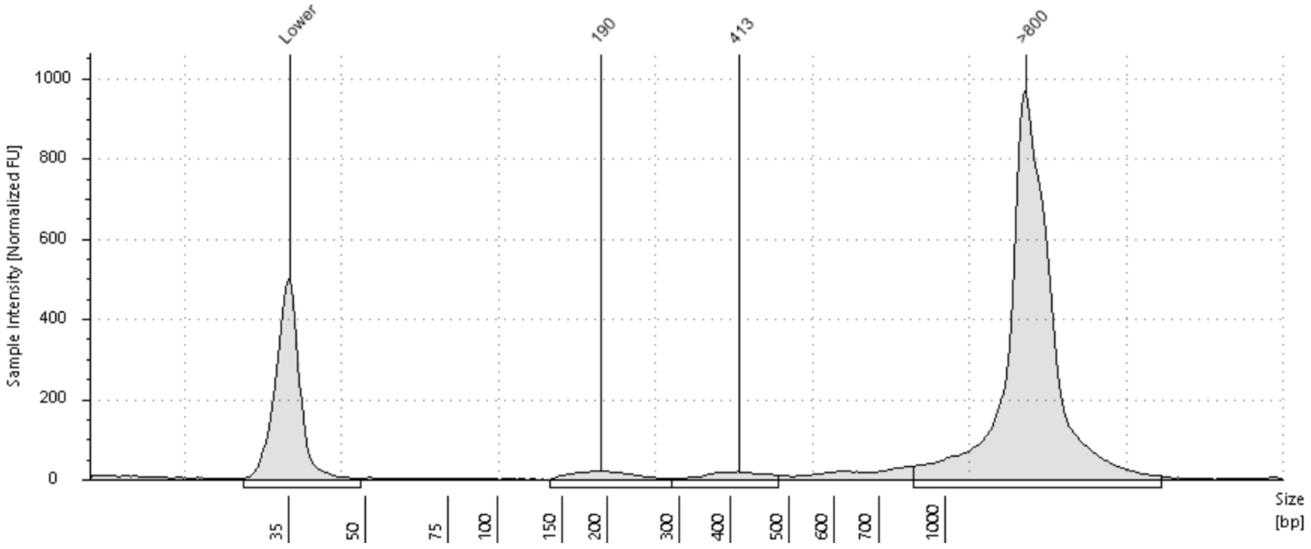

Sample Table

Well	%cfDNA	Conc. [pg/µl]	Sample Description	Alert	Observations
B2	8	1040	DNA102082	⚠	Caution! Expired ScreenTape device (used after two weeks of first use)

Peak Table

Size [bp]	Calibrated Conc. [pg/µl]	Assigned Conc. [pg/µl]	Peak Molarity [pmol/l]	% Integrated Area	Height	Peak Comment	Observations
35	275	275	12100	-	176.748		Lower Marker
190	26.6	-	215	2.70	7.211		
413	23.2	-	86.3	2.35	6.747		
>800	938	-	-	94.96	342.158		

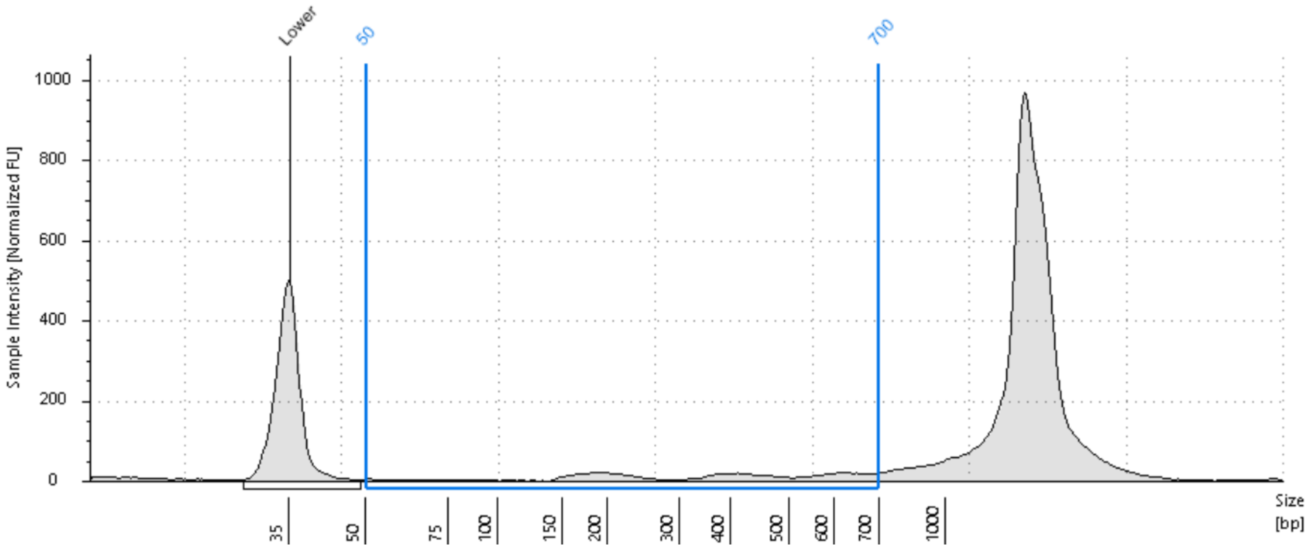

Region Table

From [bp]	To [bp]	Average Size [bp]	Conc. [pg/µl]	Region Molarity [pmol/l]	% of Total	Region Comment	Color
50	700	382	81.8	510	7.86	%cfDNA	■

© Agilent Technologies, Inc. 2021

C2: DNA102083 - D19_plastic_centrifuged

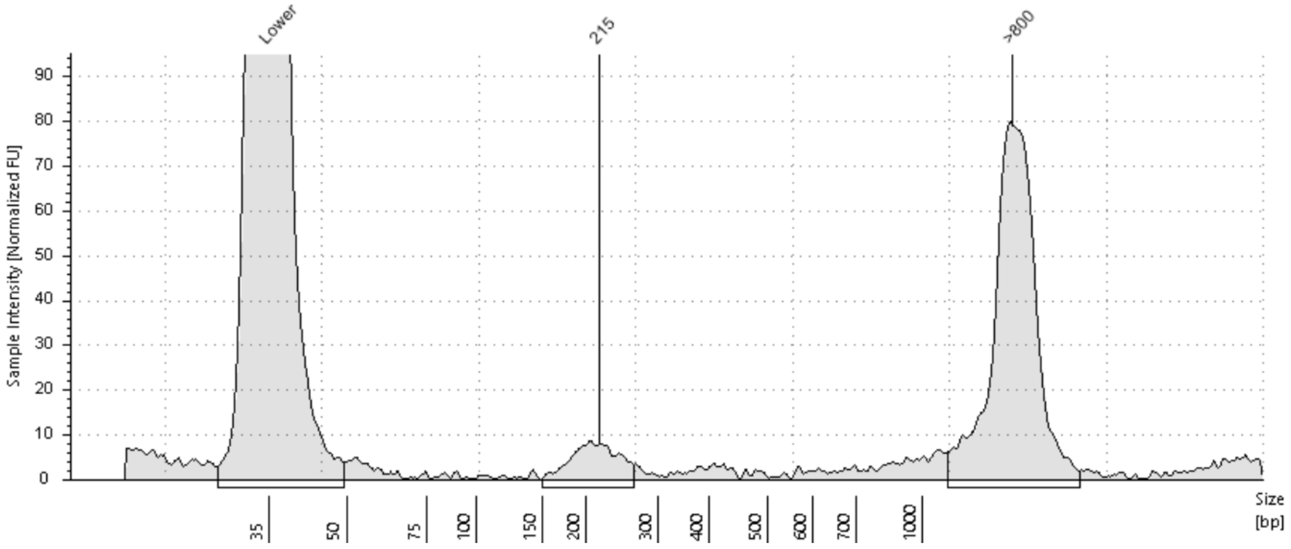

Sample Table

Well	%cfDNA	Conc. [pg/µl]	Sample Description	Alert	Observations
C2	17	92.2	DNA102083	⚠	Caution! Expired ScreenTape device; Sample concentration outside functional range for %cfDNA and the assay

Peak Table

Size [bp]	Calibrated Conc. [pg/µl]	Assigned Conc. [pg/µl]	Peak Molarity [pmol/l]	% Integrated Area	Height	Peak Comment	Observations
35	275	275	12100	-	230.622		Lower Marker
215	7.35	-	52.5	10.11	3.634		
>800	65.4	-	-	89.89	36.748		

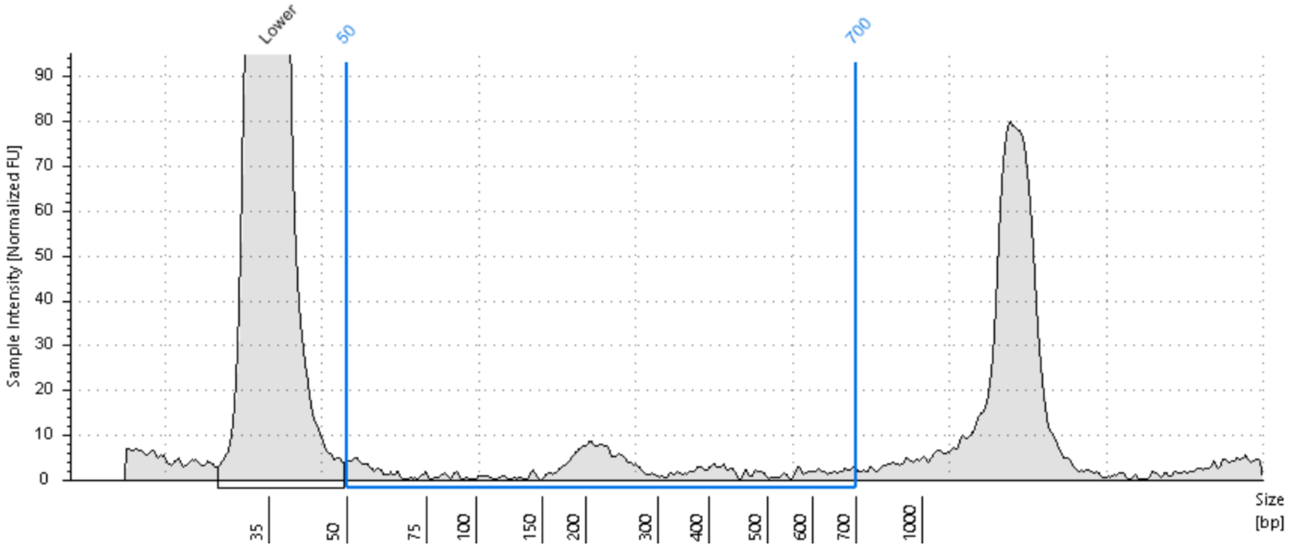

Region Table

From [bp]	To [bp]	Average Size [bp]	Conc. [pg/µl]	Region Molarity [pmol/l]	% of Total	Region Comment	Color
50	700	273	15.7	177	16.98	%cfDNA	■

© Agilent Technologies, Inc. 2021

D2: DNA102084 - D20_plastic_centrifuged

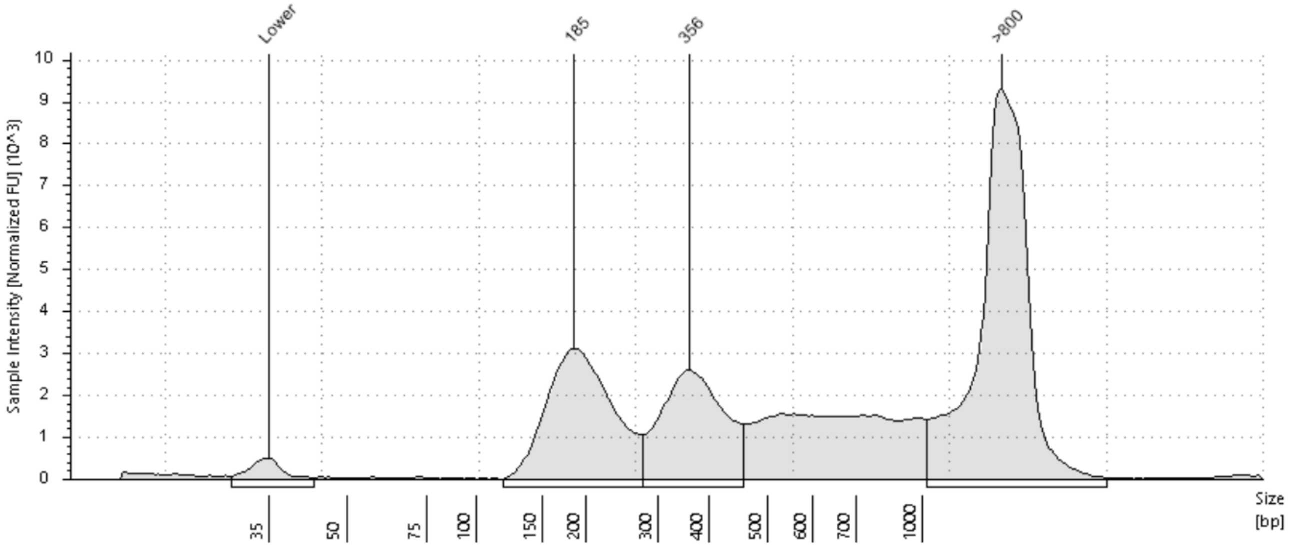

Sample Table

Well	%cfDNA	Conc. [pg/µl]	Sample Description	Alert	Observations
D2	49	19300	DNA102084	⚠	Caution! Expired ScreenTape device; Sample concentration outside functional range for %cfDNA and the assay

Peak Table

Size [bp]	Calibrated Conc. [pg/µl]	Assigned Conc. [pg/µl]	Peak Molarity [pmol/l]	% Integrated Area	Height	Peak Comment	Observations
35	275	275	12100	-	14.319		Lower Marker
185	3780	-	31400	25.14	89.058		
356	3010	-	13000	20.01	74.076		
>800	8250	-	-	54.86	266.301		

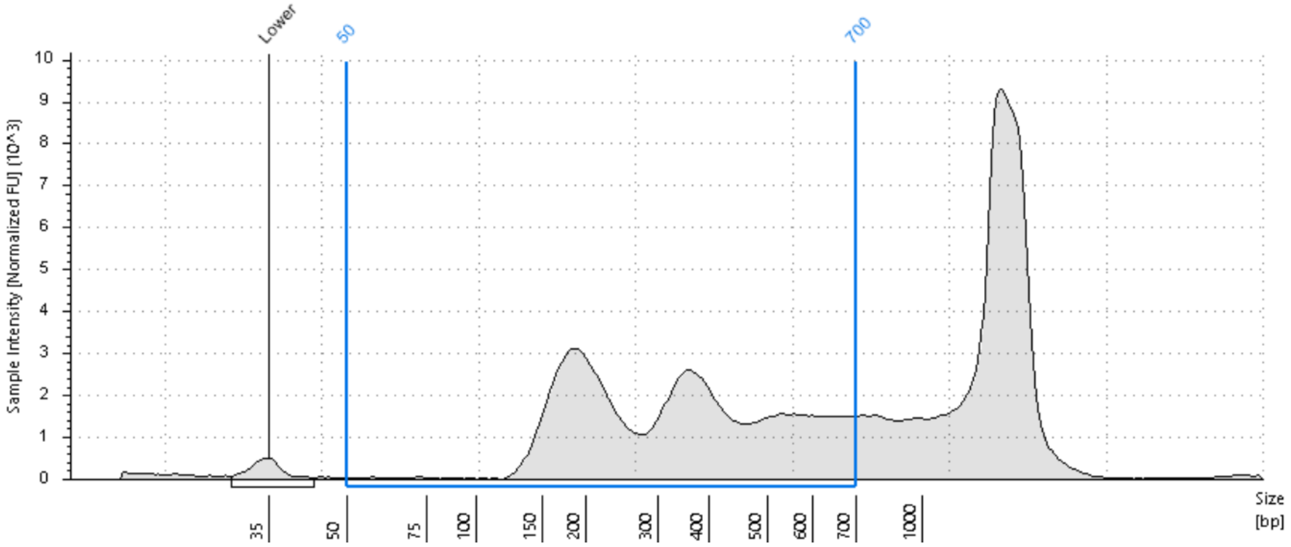

Region Table

From [bp]	To [bp]	Average Size [bp]	Conc. [pg/µl]	Region Molarity [pmol/l]	% of Total	Region Comment	Color
50	700	356	9440	51700	48.82	%cfDNA	■

© Agilent Technologies, Inc. 2021
