# Supplementary material for: Diagnosis of pediatric central nervous system tumors using methylation profiling of cfDNA from cerebrospinal fluid
Source: Clin Epigenetics. 2024 Jul 5;16:87. doi: 10.1186/s13148-024-01696-w (PMC11225235; doi:10.1186/s13148-024-01696-w)
Supplement: Supplementary file 2 — Additional file 2. [file 13148_2024_1696_MOESM2_ESM.docx]

Supplementary file 2


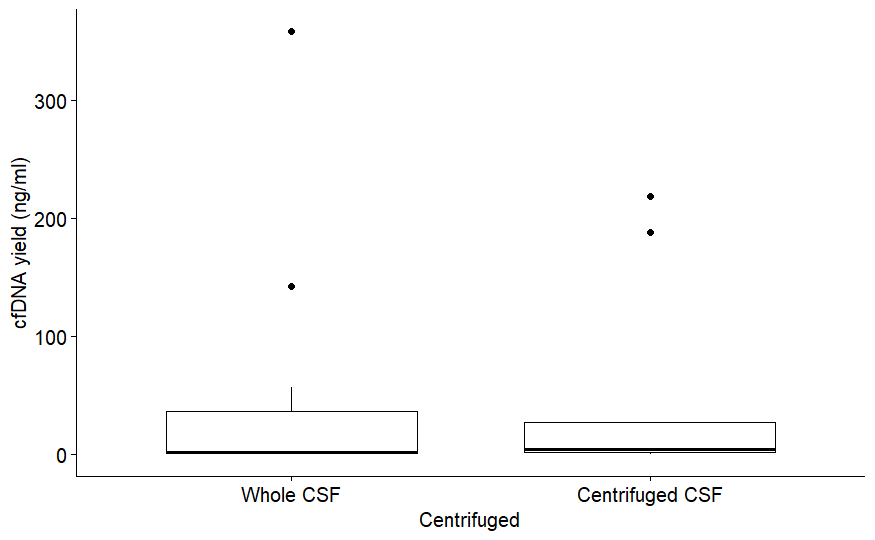


Figure 1: Total cfDNA yield in whole CSF (n=12) versus centrifuged CSF samples (n=20).


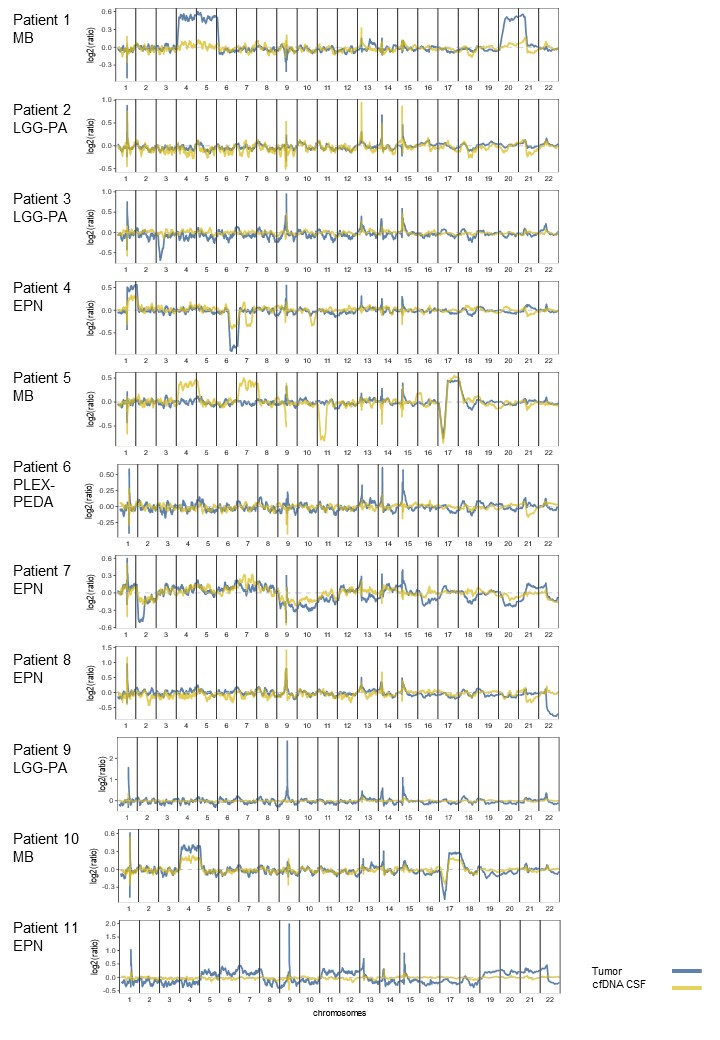


Figure 2: DNA copy number profiles of all patients with matching CSF-cfDNA and FFPE-DNA samples. Overlapping profiles of the tumor formalin fixed paraffin embedded (FFPE) material in BLUE and CSF material in YELLOW show both corresponding aberrations as well as some heterogeneity.
