# Supplementary material for: Diagnosis of pediatric central nervous system tumors using methylation profiling of cfDNA from cerebrospinal fluid
Source: Clin Epigenetics. 2024 Jul 5;16:87. doi: 10.1186/s13148-024-01696-w (PMC11225235; doi:10.1186/s13148-024-01696-w)
Supplement: Supplementary file 3 — Additional file 3. [file 13148_2024_1696_MOESM3_ESM.pdf]

Filename: 2024-06-04 - 14.56.15.cfDNA

Sample Info

Well	%cfDNA	Sample Description	Alert	Observations
A1	-	Ladder		Ladder
B1	87	Pool NVQ_185		
C1	90	Pool NVQ_264		
D1	95	Pool NVQ_526_CSF_FFPE		
E1	96	Pool NVQ_593		
F1	94	Pool NVQ_631		
G1	94	Pool NVQ_815		
H1	93	Pool NVQ_340_FFPE		

© Agilent Technologies, Inc. 2021

A1: Ladder

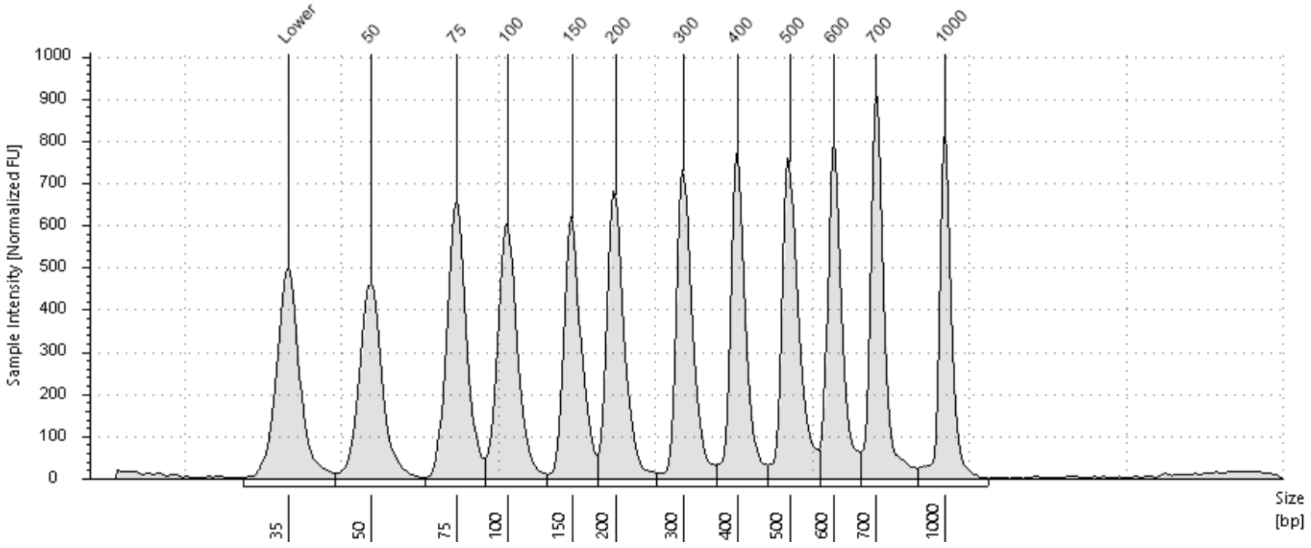

Sample Table

Well	%cfDNA	Sample Description	Alert	Observations
A1	-	Ladder		Ladder

Peak Table

Size [bp]	Calibrated Conc. [pg/µl]	Assigned Conc. [pg/µl]	Peak Molarity [pmol/l]	% Integrated Area	Height	Peak Comment	Observations
35	275	275	12100	-	64.555		Lower Marker
50	248	-	7630	8.21	59.666		
75	310	-	6360	10.27	85.234		
100	280	-	4300	9.25	78.093		
150	255	-	2610	8.43	79.907		
200	274	-	2110	9.07	87.882		
300	271	-	1390	8.98	94.505		
400	280	-	1080	9.27	100.423		
500	296	-	910	9.79	97.880		
600	269	-	690	8.91	103.497		
700	300	-	659	9.92	118.872		
1000	239	-	367	7.90	106.263		

 © Agilent Technologies, Inc. 2021

B1: Pool NVQ_185

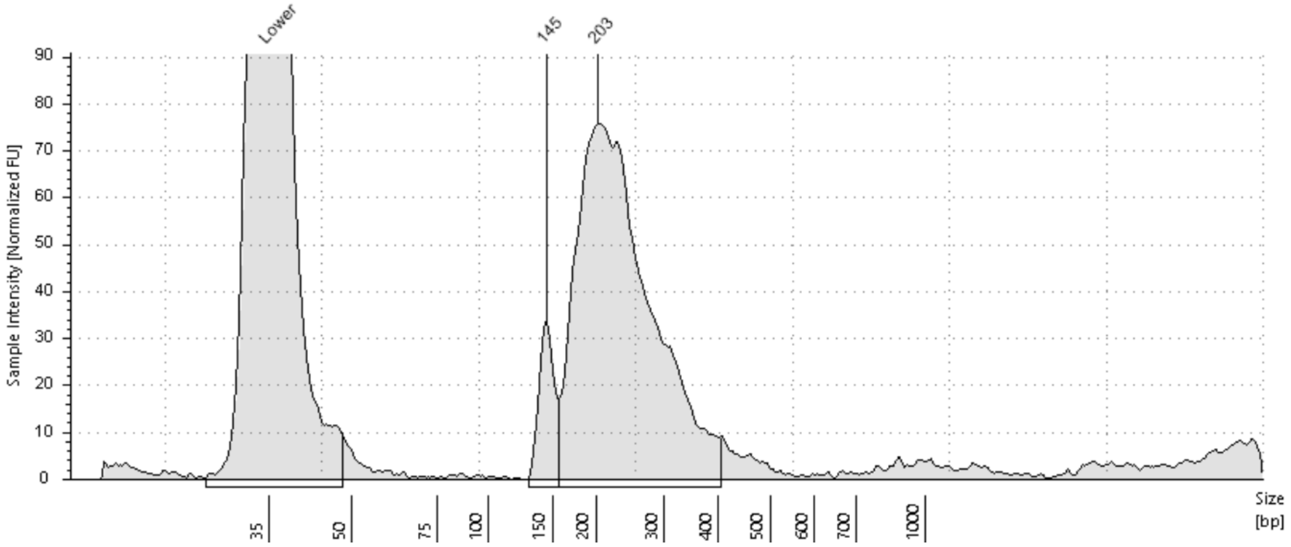

Sample Table

Well	%cfDNA	Sample Description	Alert	Observations
B1	87	Pool NVQ_185		

Peak Table

Size [bp]	Calibrated Conc. [pg/µl]	Assigned Conc. [pg/µl]	Peak Molarity [pmol/l]	% Integrated Area	Height	Peak Comment	Observations
35	275	275	12100	-	302.894		Lower Marker
145	10.4	-	111	8.54	20.424		
203	112	-	846	91.46	45.928		

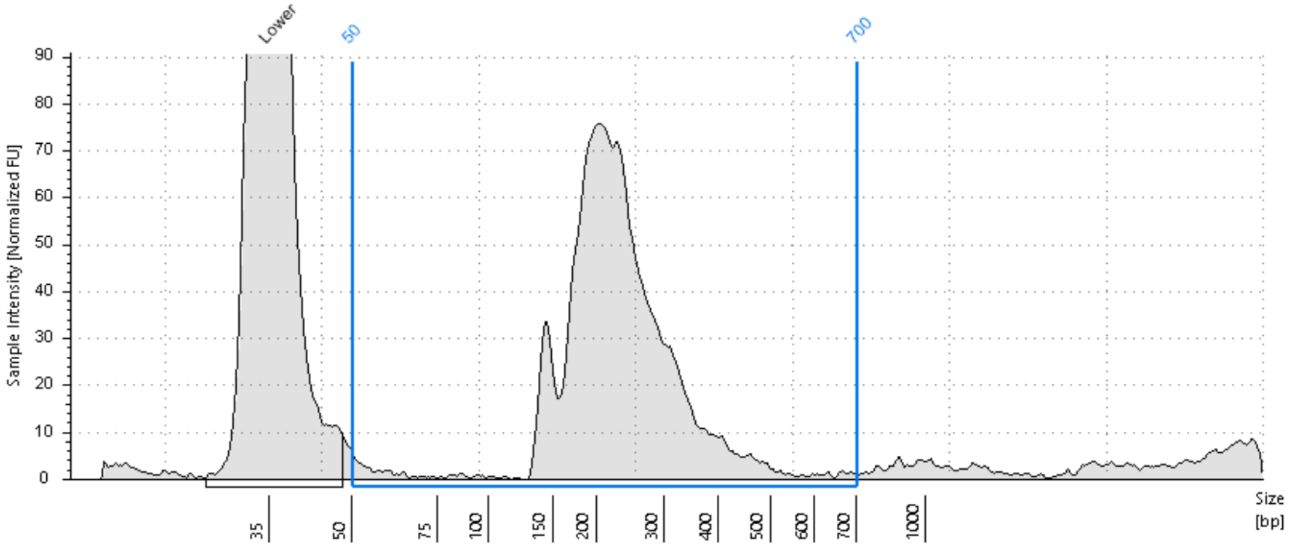

Region Table

From [bp]	To [bp]	Average Size [bp]	Conc. [pg/µl]	Region Molarity [pmol/l]	% of Total	Region Comment	Color
50	700	238	128	966	87.10	%cfDNA	

© Agilent Technologies, Inc. 2021

C1: Pool NVQ_264

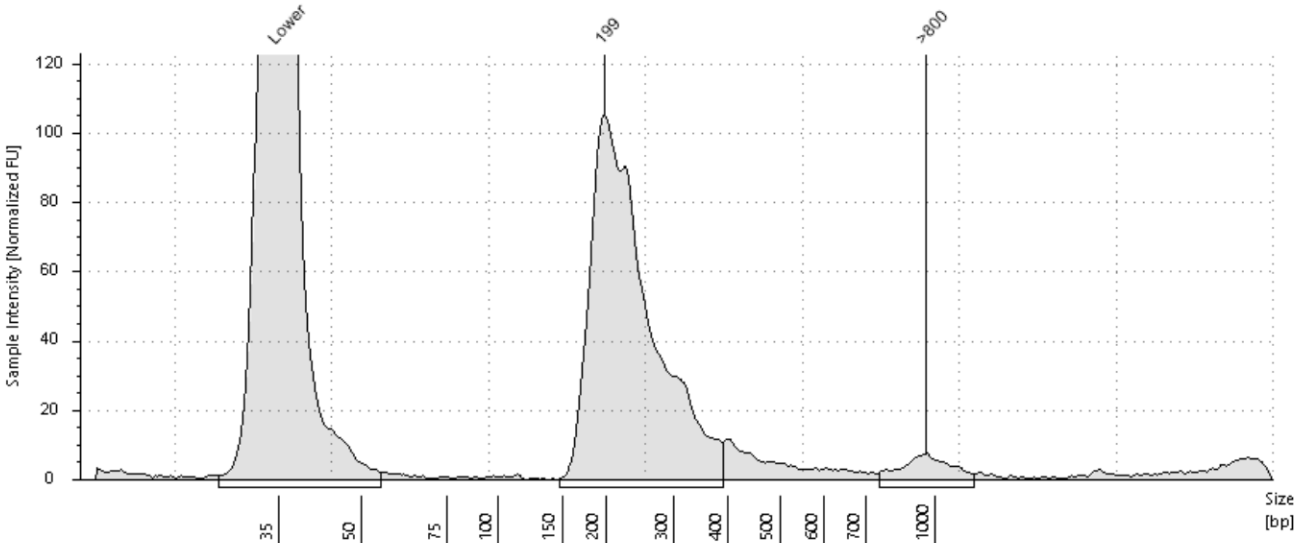

Sample Table

Well	%cfDNA	Sample Description	Alert	Observations
C1	90	Pool NVQ_264		

Peak Table

Size [bp]	Calibrated Conc. [pg/µl]	Assigned Conc. [pg/µl]	Peak Molarity [pmol/l]	% Integrated Area	Height	Peak Comment	Observations
35	275	275	12100	-	382.775		Lower Marker
199	123	-	951	95.17	80.617		
>800	6.26	-	-	4.83	5.731		

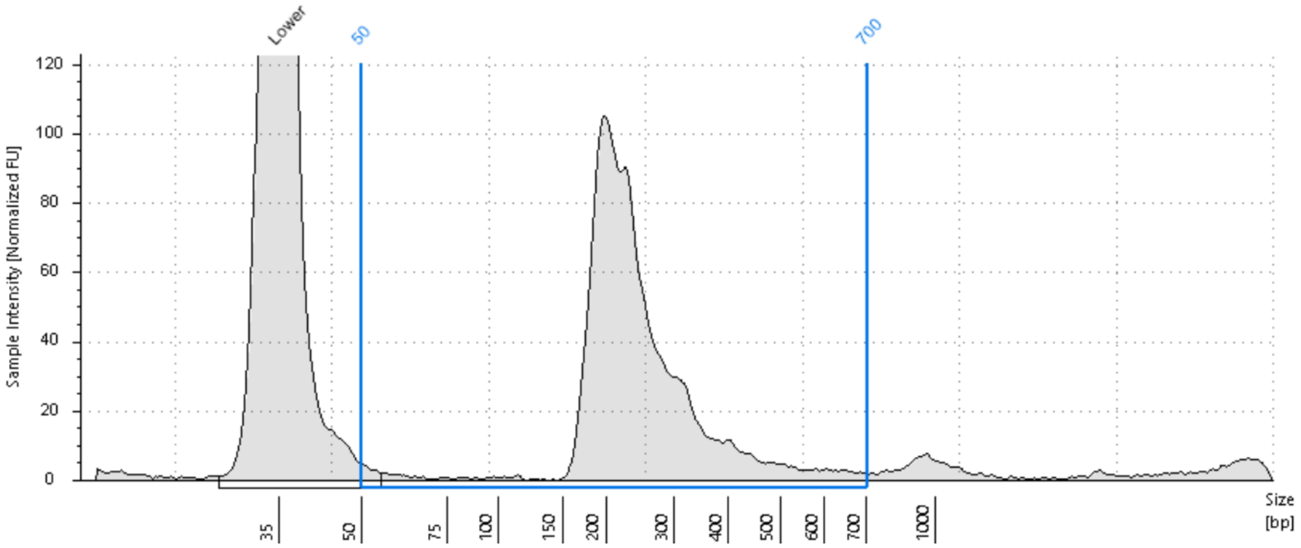

Region Table

From [bp]	To [bp]	Average Size [bp]	Conc. [pg/µl]	Region Molarity [pmol/l]	% of Total	Region Comment	Color
50	700	255	135	950	90.04	%cfDNA	

© Agilent Technologies, Inc. 2021

D1: Pool NVQ_526_CSF_FFPE

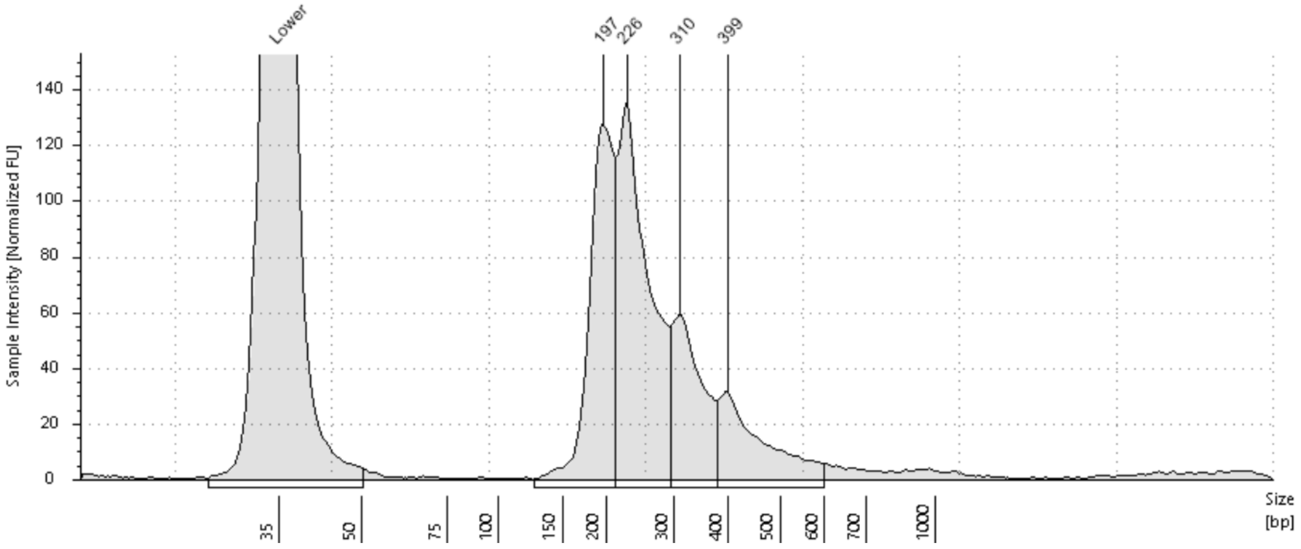

Sample Table

Well	%cfDNA	Sample Description	Alert	Observations
D1	95	Pool NVQ_526_CSF_FFPE		

Peak Table

Size [bp]	Calibrated Conc. [pg/µl]	Assigned Conc. [pg/µl]	Peak Molarity [pmol/l]	% Integrated Area	Height	Peak Comment	Observations
35	275	275	12100	-	438.156		Lower Marker
197	62.2	-	486	29.27	111.934		
226	87.3	-	593	41.09	118.659		
310	35.4	-	176	16.65	52.056		
399	27.6	-	106	12.99	27.608		

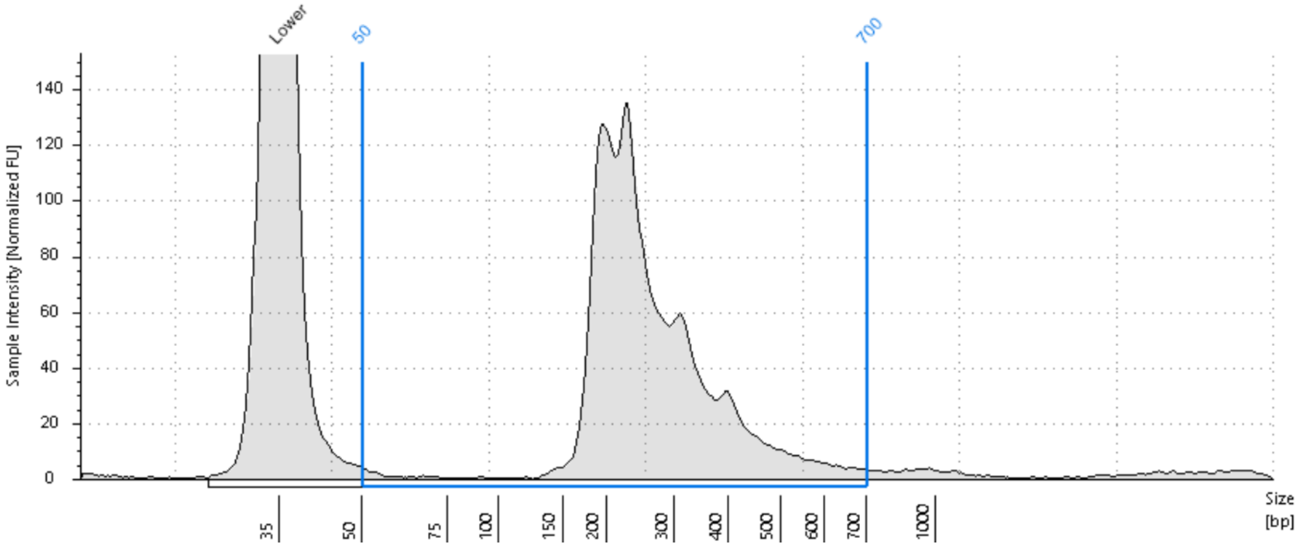

Region Table

From [bp]	To [bp]	Average Size [bp]	Conc. [pg/µl]	Region Molarity [pmol/l]	% of Total	Region Comment	Color
50	700	274	213	1360	95.23	%cfDNA	

© Agilent Technologies, Inc. 2021

E1: Pool NVQ_593

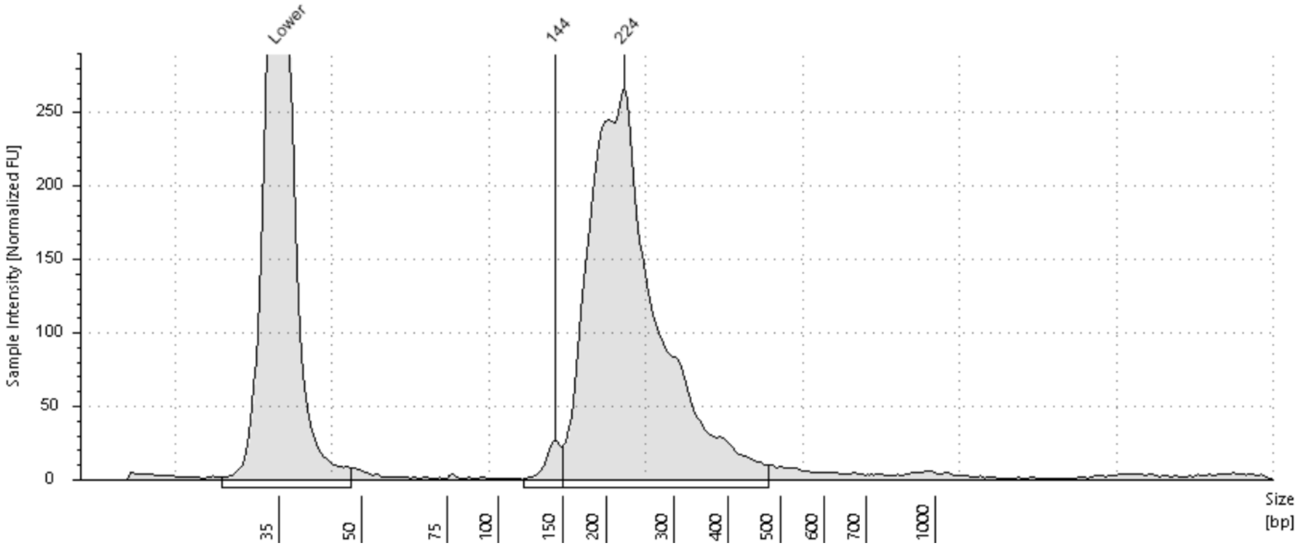

Sample Table

Well	%cfDNA	Sample Description	Alert	Observations
E1	96	Pool NVQ_593		

Peak Table

Size [bp]	Calibrated Conc. [pg/µl]	Assigned Conc. [pg/µl]	Peak Molarity [pmol/l]	% Integrated Area	Height	Peak Comment	Observations
35	275	275	12100	-	286.166		Lower Marker
144	8.08	-	86.4	2.15	15.273		
224	368	-	2530	97.85	152.560		

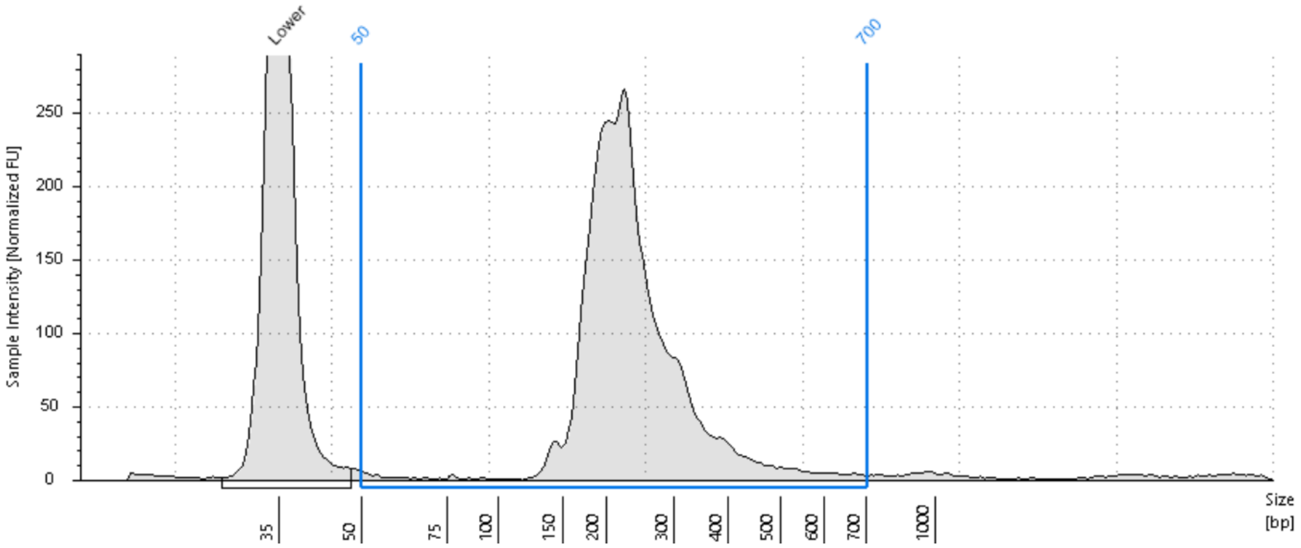

Region Table

From [bp]	To [bp]	Average Size [bp]	Conc. [pg/µl]	Region Molarity [pmol/l]	% of Total	Region Comment	Color
50	700	245	387	2710	96.41	%cfDNA	

© Agilent Technologies, Inc. 2021

F1: Pool NVQ_631

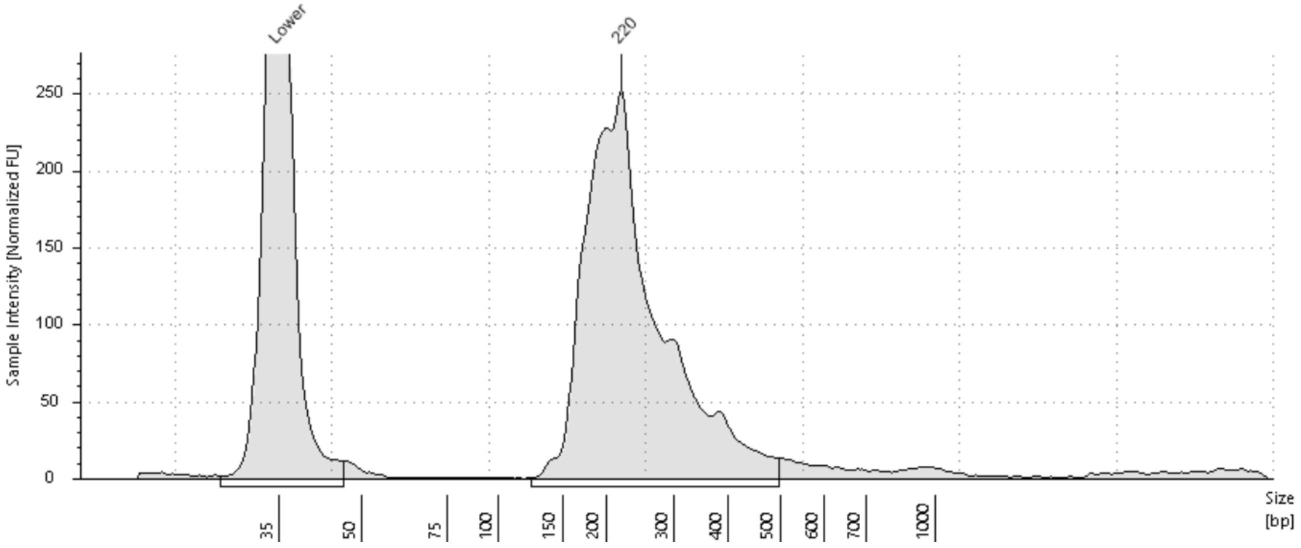

Sample Table

Well	%cfDNA	Sample Description	Alert	Observations
F1	94	Pool NVQ_631		

Peak Table

Size [bp]	Calibrated Conc. [pg/µl]	Assigned Conc. [pg/µl]	Peak Molarity [pmol/l]	% Integrated Area	Height	Peak Comment	Observations
35	275	275	12100	-	302.205		Lower Marker
220	385	-	2690	100.00	152.796		

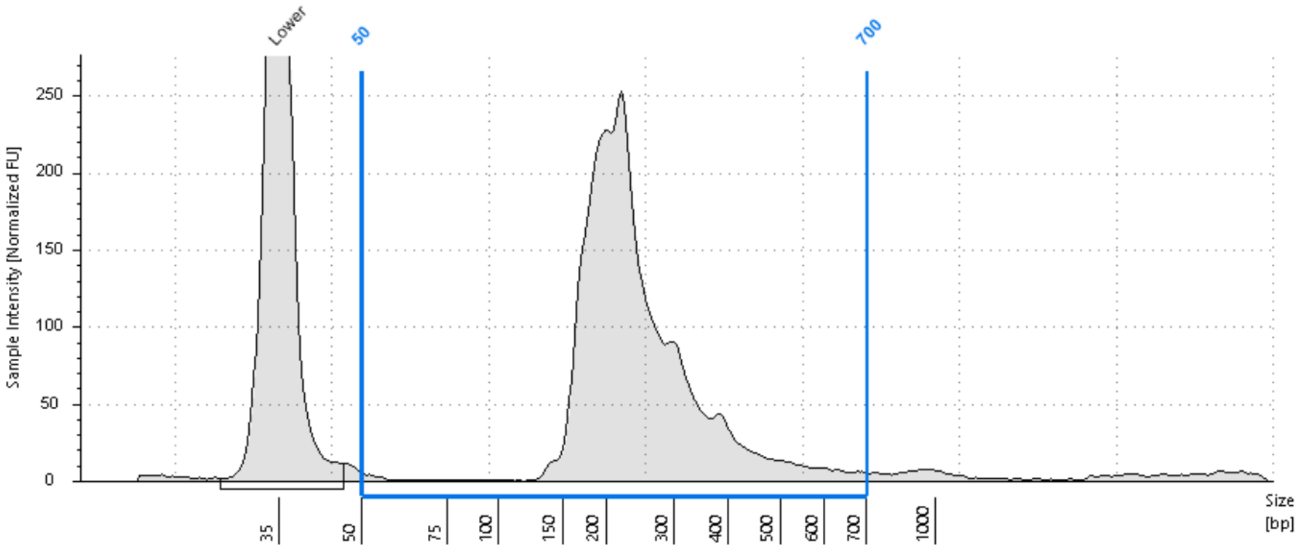

Region Table

From [bp]	To [bp]	Average Size [bp]	Conc. [pg/µl]	Region Molarity [pmol/l]	% of Total	Region Comment	Color
50	700	254	399	2710	93.86	%cfDNA	

© Agilent Technologies, Inc. 2021

G1: Pool NVQ_815

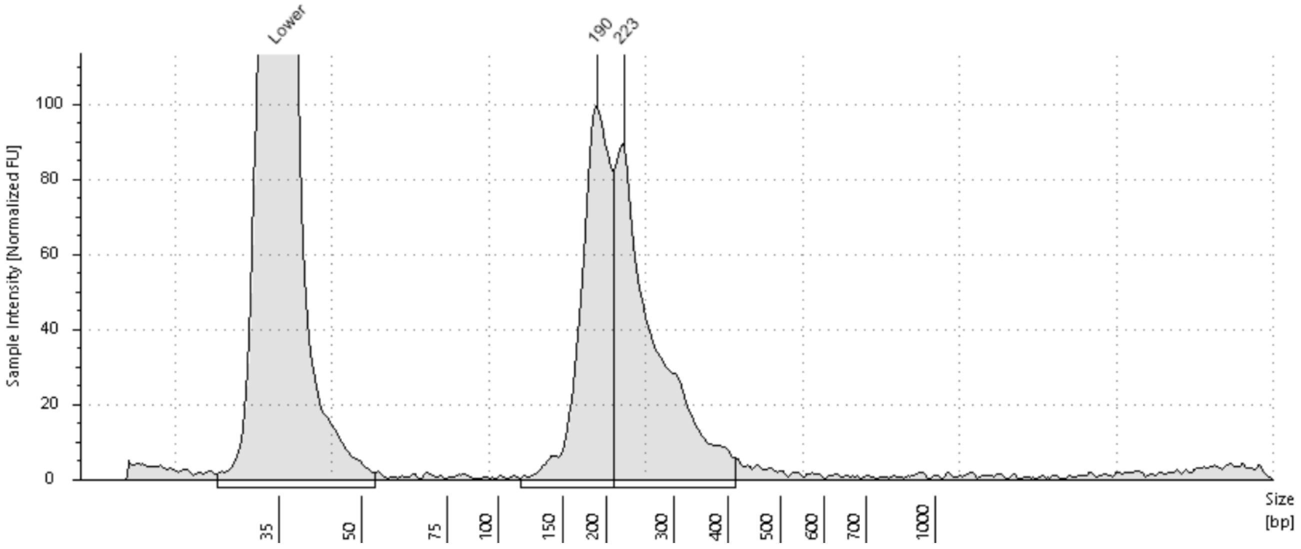

Sample Table

Well	%cfDNA	Sample Description	Alert	Observations
G1	94	Pool NVQ_815		

Peak Table

Size [bp]	Calibrated Conc. [pg/µl]	Assigned Conc. [pg/µl]	Peak Molarity [pmol/l]	% Integrated Area	Height	Peak Comment	Observations
35	275	275	12100	-	297.052		Lower Marker
190	58.1	-	471	44.34	59.030		
223	73.0	-	504	55.66	52.992		

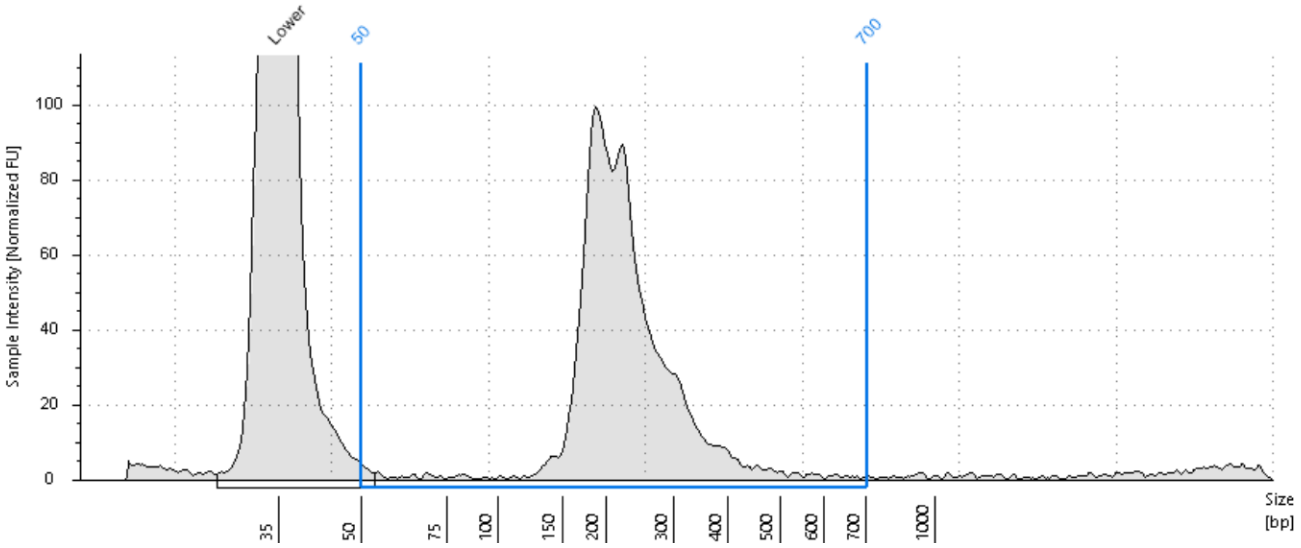

Region Table

From [bp]	To [bp]	Average Size [bp]	Conc. [pg/µl]	Region Molarity [pmol/l]	% of Total	Region Comment	Color
50	700	235	134	999	94.34	%cfDNA	

© Agilent Technologies, Inc. 2021

H1: Pool NVQ_340_FFPE

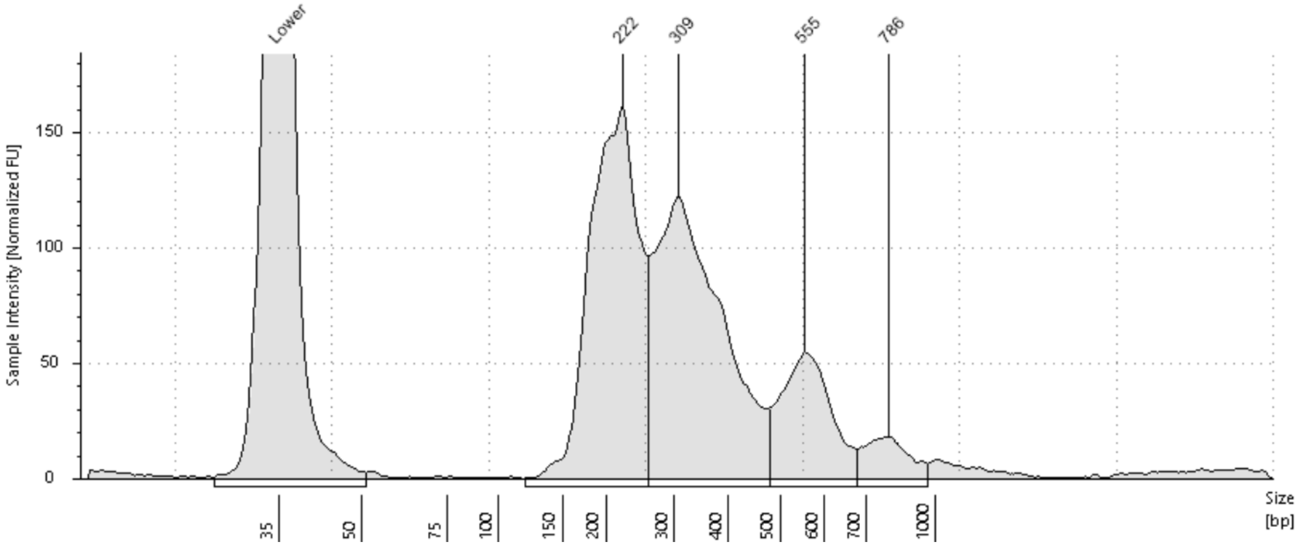

Sample Table

Well	%cfDNA	Sample Description	Alert	Observations
H1	93	Pool NVQ_340_FFPE		

Peak Table

Size [bp]	Calibrated Conc. [pg/µl]	Assigned Conc. [pg/µl]	Peak Molarity [pmol/l]	% Integrated Area	Height	Peak Comment	Observations
35	275	275	12100	-	331.942		Lower Marker
222	162	-	1130	39.70	107.155		
309	173	-	862	42.34	81.139		
555	56.8	-	158	13.91	36.441		
786	16.6	-	32.4	4.05	12.095		

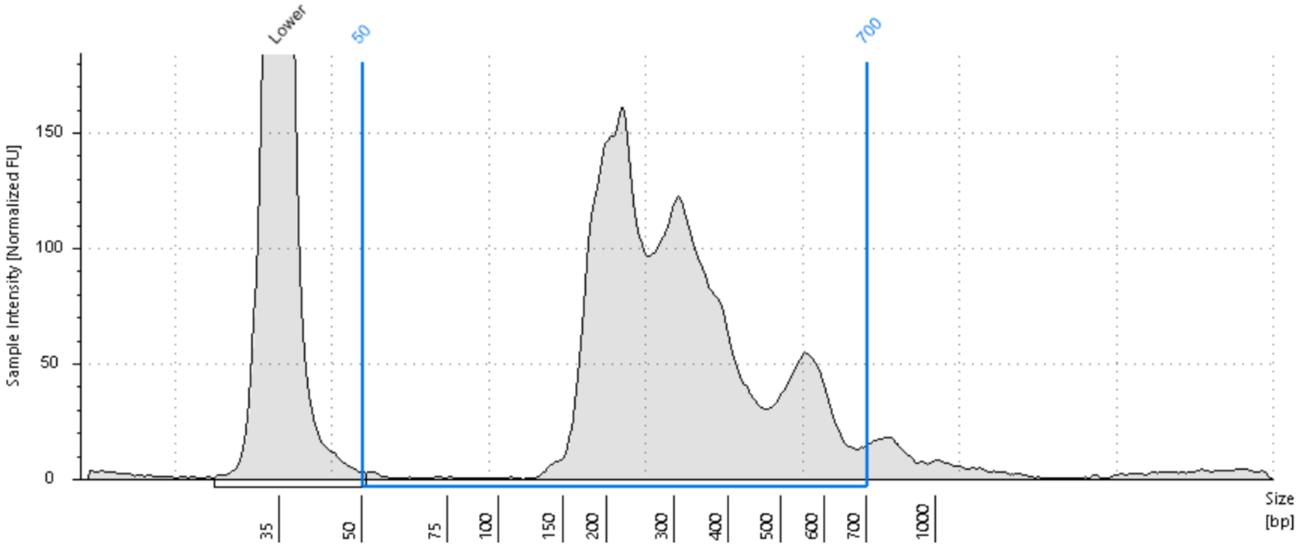

Region Table

From [bp]	To [bp]	Average Size [bp]	Conc. [pg/µl]	Region Molarity [pmol/l]	% of Total	Region Comment	Color
50	700	320	393	2220	93.19	%cfDNA	

© Agilent Technologies, Inc. 2021

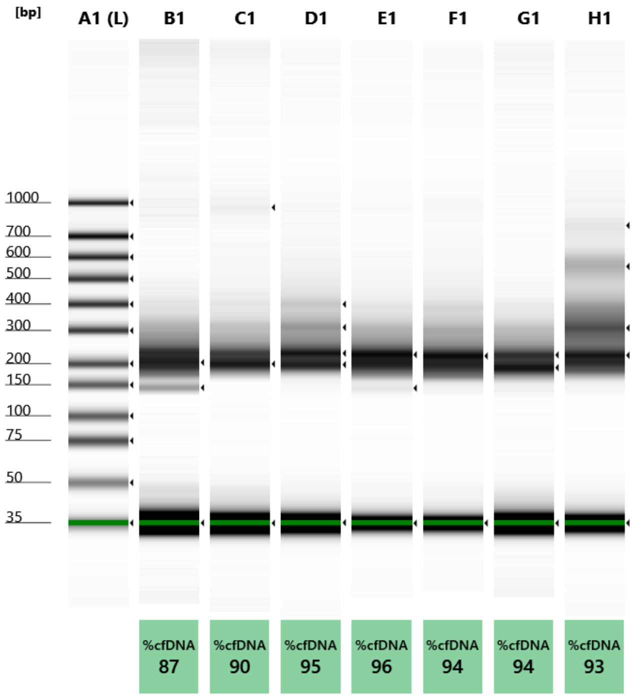

Default image (Contrast 50%), Image is Scaled to Sample

© Agilent Technologies, Inc. 2021
